# A Very Readily Prepared Ligand for Rhodium Catalyzed Propene Hydroformylation

**DOI:** 10.1002/chem.202500917

**Published:** 2025-06-03

**Authors:** José A. Fuentes, David B. Cordes, Aidan P. McKay, Mesfin E. Janka, Matthew L. Clarke

**Affiliations:** ^1^ EaSTCHEM School of Chemistry University of St Andrews St Andrews Fife UK; ^2^ Eastman Chemical Company 200 South Wilcox Drive Kingsport Tennessee 37660 USA

**Keywords:** high pressure infra red spectroscopy, homogeneous catalysis, hydroformylation, ligand design, regioselective

## Abstract

In the search for an easy‐to‐make ligand that would enable Rh catalyzed hydroformylation of propene to favor the branched aldehydes, *iso*‐butanal, *bis*‐phosphonites and *bis*‐phosphoramidites were designed. Catalysts derived from *bis*‐phosphonites gave the desired low *n:iso* ratios, but were unstable to hydrolysis. A *bis*‐phosphoramidite ligand (EasyDiPhos) that can be made in just one synthetic step has been discovered that enables Rh‐catalyzed hydroformylations to proceed with unusually low *n:iso* ratios. They were also found to have significant stability to moisture, air, and to relatively high temperatures during hydroformylation conditions. The latter was studied using a combination of high pressure infra‐red (HPIR) spectroscopy and NMR analysis of the catalyst resting state after 1 week under relevant conditions. Propene hydroformylation with *n:iso* ratios well below 1 was carried out and with turnover numbers around 1000 mol/mol in 1 hour reaction time. One of the ligands, whilst not being the most *iso*‐selective example, had a partially flexible *n:iso* ratio depending on temperature and pressure. Two Pt(II) complexes were prepared and their X‐ray crystal structures determined. The most *iso*‐selective catalyst was examined at low temperature (prioritizing *iso*‐selectivity over rates) in hydroformylation of some other terminal alkenes with significant branched selectivity.

## Graphical abstract

Recent developments aimed at switching the selectivity of rhodium catalyzed hydroformylation from a linear‐selective to a branched selective reaction feature ligands that are relatively complicated to prepare. A new ligand, EasyDiPhos, can be made in just one synthetic step and gives unusually low *n:iso* ratios, i.e., favoring the branched aldehyde, *iso*‐butanal, in propene hydroformylation.

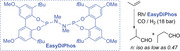

## 1 Introduction

**Figure 1:**
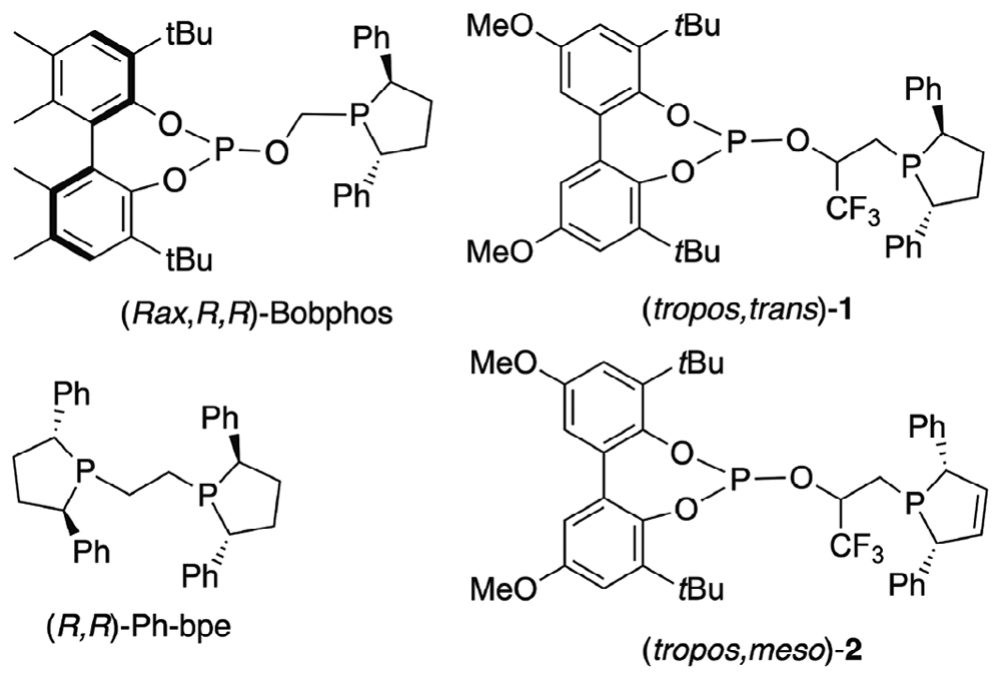
Structure of various phosphine‐phosphites that give unusually low *n:iso* ratios in hydroformylation of unbiased terminal alkenes.

The hydroformylation of alkenes is one of the most industrially important reactions in the entire field of homogeneous catalysis.^[^ ^1^, ^2^, ^3^ ^]^ Hydroformylation of electronically unbiased terminal alkenes, such as propene, either modestly favors the linear, *n*‐isomer for most catalysts, or strongly favors this isomer using wide bite angle *bis*‐phosphorous ligands.^[^ ^1^, ^2^, ^3^, ^4^, ^5^, ^6^, ^7^, ^8^ ^]^ The last few years have seen some interest and developments in forming the more branched (*iso*) aldehydes.^[^ ^9^, ^10^, ^11^, ^12^, ^13^, ^14^, ^15^, ^16^, ^17^, ^18^, ^19^, ^20^, ^21^, ^22^, ^23^, ^24^, ^25^, ^26^, ^27^, ^28^, ^29^ ^]^ For applications in organic synthesis, the highest possible regioselectivity is sought after‐ with rates, catalyst loadings, and stability of secondary importance. Some of these applications also require enantioselectivity or diastereoselectivity.^[^ ^22^, ^23^, ^24^, ^25^, ^26^, ^27^, ^28^, ^29^ ^]^ In the case of industrial propene hydroformylation,^[^ ^9^, ^10^, ^11^, ^12^, ^13^, ^14^, ^15^, ^16^, ^17^, ^18^, ^19^, ^20^, ^21^ ^]^ since a totally selective process with good rates is seen as being quite far away, and the isomers are separable, the approach is to produce a mixture of both isomers with as much *iso*‐butanal made per hour as possible (TOFs of at least several hundred mol/mol/h is a reasonable threshold for possible consideration for scale‐up).^[^ ^9^, ^10^, ^11^, ^12^, ^13^, ^14^, ^15^, ^16^, ^17^, ^18^, ^19^, ^20^, ^21^ ^]^ In this sub‐field, *n:iso* ratios are generally quoted due to the importance of industrial propene hydroformylation and this being the universally used term for the selectivity output of *n‐*butanal and *iso*‐butanal. In this article, we will quote both *n:iso* and % branched for ease of understanding. In the hydroformylation of propene, the maximum branched selectivity peaks at around 82% (*n:iso* = 0.22), but more relevantly *n:iso* ratios of around 0.43 (70% branched) can be realized at 90–105 °C with a TOF around 670 mols product/mol catalyst/h. One example of this catalyst class also completed a 4‐day stability test using real feedstocks with no change in performance over time giving a total TON of over 250,000 mols product/mol catalyst.^[^ ^15^ ^]^ Whilst, we streamlined this ligand class further with ligand **2** (Figure 1),^[^ ^19^ ^]^ the relatively long synthesis of these ligands means that there would most likely be a cost premium to using these more selective catalysts in a large‐scale industrial process.

Propene hydroformylation catalysts that give an *n:iso* ratio below 0.66 (i.e ∼60% *iso*‐butanal or more) with good rates are very hard to find. In parallel with developing the Bobphos ligand family, we undertook a study to see if there were any easy‐to‐make catalysts that give relatively low *n:iso* ratio in propene hydroformylation. Over the course of over a decade of experiments, our own testing of many different Rh catalysts did not uncover easily made catalysts that offer *n:iso* ratio below 0.66 whilst giving TOF above a few hundred mol prod/mol catalyst/h—excepting what is reported here. Highlights from the literature in this time have not delivered this either. For example, Ref [17] describes *n:iso* ratio below 0.1 but with very low TOF only around 1 mol prod/mol catalyst/h, Ref [14] reports *n:iso* of 1.1 with TOF > 500 and *n:iso* of 0.74 but with TOF of 75 mol prod/mol catalyst/h. Ref [18] reports *n:iso* of 0.74 with TOF> 500 mol prod/mol catalyst/h. Ref [20] reports *n:iso* of 1.1 with TOF > 20,000. Here, we show how ligands made in just one synthetic step can form rhodium catalysts that give unusually low *n:iso* ratio in propene hydroformylation at industrially relevant temperatures (and rates).

## 2 Results and Discussion

**Scheme 1:**
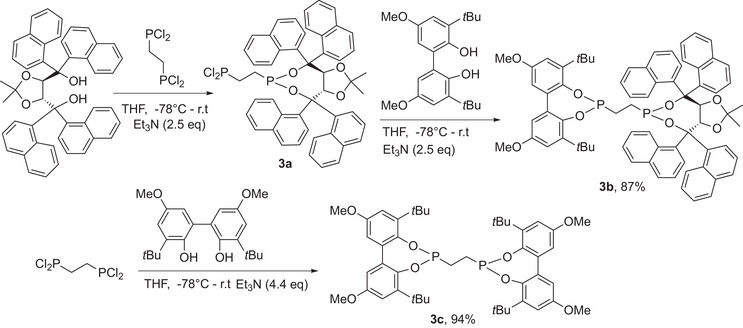
Synthesis of an asymmetrically substituted *bis*‐phosphonite ligand.

Our design strategy was to consider what might be the key features in the Bobphos family of ligands. Whilst, we had previously found no strong correlation with the tendency to form equatorial–axial Rh complexes rather than *bis*‐equatorial Rh complexes,^[^ ^9^ ^]^ it seemed reasonable to keep a broadly similar bite angle to the Rh/Bobphos catalysts. Additionally, our mechanistic studies suggested the bulky phosphite moiety in Rh/Bobphos catalysts closes off some pathways for hydroformylation, so part of the structure should be analagous to a bulky phosphite. Thirdly, these studies suggests that the Rh‐linear alkyl chain that would eventually form linear aldehydes becomes trapped by the phenyl–phospholane unit. The novel *bis*‐phosphonite, **3b** (Scheme 1) was therefore designed with the thought that part of the naphthyl ring system might be in a similar relative location as the phospholano phenyl groups in Bobphos. The other end of the ligand is very similar to ligand **1**. Asymmetrically substituted *bis*‐phosphonites of this type were not known, but we hoped that introducing the bulky naphthyl‐TADDOL diol first might make it possible to form each phosphonite terminus selectively in a step‐wise fashion.

The two stage synthesis of **3b** can indeed be conducted in one synthetic operation to give crude **3b** with very few side products or starting materials remaining. Conducting the synthesis in inverse order with the biphenol first gives an intermediate that is contaminated with starting material and the symmetric *bis*‐phosphonite, **3c**. The percentage impurity of **3c** in the desired dichlorophosphine (not pictured) was between 8% and 65%, whereas when the bulkier TADDOL diol was added first the desired dichlorophosphine, **3a** had very little impurity. The symmetric *bis*‐phosphonite, **3c** was then also prepared deliberately for comparison. Both ligands were then subjected to a standard protocol we have used many times for assessing catalysts for propene hydroformylation. The protocol uses a catalyst preactivation step, and then measures TON after 1 hour reaction time as an approximate initial TOF for these reactions, although for fast catalysts at higher temperatures the reactions are well beyond 50% conversion at this point and hence, may be an underestimate of an initial TOF. Consequently, a shorter reaction time of 30 minutes is used in some examples.

We were delighted to find that the novel catalyst derived from ligand **3b** (crude, ∼97%–99% purity) and [Rh(acac)(CO)_2_] gives very unusually low *n:iso* ratio in propene hydroformylation (Table 1). All attempts at purifying or any manipulations on ligand **3b** or **3c** resulted in total decomposition. Even exposure to air resulted in decomposition of this ligand, with NMR analysis showing this is a hydrolysis reaction based on large ^1^ *J*_P‐H_ coupling constants that P─OH/P(═O)H tautomers exhibit. We could obtain HPIR spectroscopic evidence (see ESI, section 15) that clean catalyst formation does take place, and hence the catalysis results are derived from an Rh complex of ligand **3b**. While it has been shown that the stability of P‐O bonds can be engineered by structural modifications,^[^ ^30^ ^]^ the extreme hydrolytic sensitivity was not going to be suitable for consideration for industry.

**Table 1:** Rh‐catalyzed hydroformylation of propene using *bis*‐phosphonite ligands.^[^ ^a^ ^]^

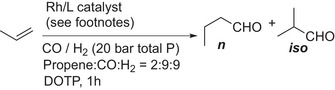					
Entry^[^ ^a^ ^]^	Ligand	T [°C]	Solvent	TON 1hour	% Branched [*n:iso*]
1	**3b** ^[^ ^b^ ^]^	50	*n*‐dodecane	92	72.0 [0.39]
2	**3b** ^[^ ^b^ ^]^	75	*n*‐dodecane	558	66.4 [0.51]
3	**3b** ^[^ ^b^ ^]^	90	*n*‐dodecane	1099	62.2 [0.61]
4	**3c**	50	*n*‐dodecane	73	63.9 [0.56]
5	**3c**	75	*n*‐dodecane	518	60.3 [0.66]
6	**3c**	75	DOTP	554	60.6 [0.65]
7	**3c**	80	DOTP(90%)	689	59.8 [0.67]
8	**3c**	90	*n*‐dodecane	993	59.3 [0.68]
9	**3c**	95	DOTP(90%)	973	58.4 [0.71]
10	**3c**	110	DOTP(90%)	1199	57.5 [0.74]

^[a]^ Catalyst preformed from [Rh(acac)(CO)_2_] (5.12 × 10^−3^ mmol) and ligand (10.24 × 10^−3^ mmol (L:Rh 2:1)) by stirring at 20 bar CO/H_2_ at 75 °C (3b, 3c, 50 min), in n‐dodecane, DOTP (DOTP = Dioctyl‐terephthalate) or DOTP (90%) (18 mL + 2 mL toluene) and then increasing or decreasing the temperature to the required temperature prior to running reaction at time specified using propene/CO/H_2_ in 1:4.5:4.5 ratio (20 bar initial pressure). Rh concentration = 2.52 × 10^−4^ mol dm^−3^. Product determined by GC using 1‐methylnaphthalene as an internal standard (full conversion TON ∼1450);

^[b]^ 97% purity, impurity being ligand 3c.

Surprisingly, the symmetric ligand, **3c**, which is structurally more distinct from Bobphos and its derivatives, also gave unusually low *n:iso* ratios in propene hydroformylation. Whilst this ligand does not have the naphthyl rings from taddol to mimic the phenylphospholane group, the level of % *iso*‐butanal is very unusual. Unfortunately, this ligand was also very moisture sensitive, so we do not recommend these *bis*‐phosphonites for general application. The *bis*‐phosphoramidite analogues of **3b/3c** were considered since they might be more stable catalysts that also deliver an unusually low *n:iso* ratio in propene hydroformylation.

**Scheme 2:**
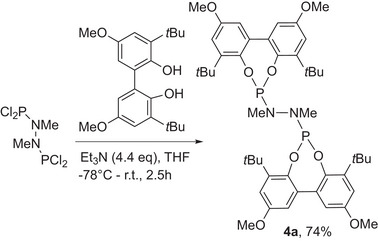
Synthesis of symmetrically substituted *bis*‐phosphoramidite, **4a**.

We elected to prepare the simplest symmetrical *bis*‐phosphoramidite **4a**. This synthesis proceeds cleanly from the commercially available *tropos* diol, 3,3′di‐tert‐butyl‐5,5′‐dimethoxy‐[1,1′biphenyl]‐2,2′diol and 1,2‐*bis*(dichlorophosphino)‐1,2‐dimethylhydrazine. Ligand **4a** can be isolated in 74% yield in one easy synthetic manipulation (Scheme 2). In complete contrast to **3b** and **3c**, **4a** can be purified using silica gel chromatography. In addition, solutions of **4a** are unchanged after being treated with a drop of water and left in air for one week, at which point the test was discontinued [ESI, Figure S7 (**3c**), Figure S8 (**4a**)].

Our theory that *bis*‐phosphoramidites would be more stable was therefore confirmed, but more pleasingly, Rh catalysts derived from this ligand display very unusually low *n:iso* ratio in propene hydroformylation: *n:iso* down to 0.47 at lower temperature and 0.57 at 80 °C Activity is also good with over 1000 turnovers in a 1‐hour reaction at just 95 °C. (Table 2). Running the reaction in a well‐stirred vessel where pressure was kept constant and syngas topped up as it was consumed, with the drop in pressure in the ballast tank recorded over time enables a curve showing gas uptake for essentially all the reaction to be obtained (ESI, section 8), showing the reactions do run to completion.

**Table 2:** Hydroformylation of propene using a Rh catalyst derived from *bis*‐phosphoramidite **4a**.^[^ ^a^ ^]^

Entry^[^ ^a^ ^]^	T [°C]	Solvent	TON [time/hour]	% Branched [*n:iso*]
1^[^ ^b^ ^]^	50	*n*‐dodecane	140 [3]	67.8 [0.47]
2^[^ ^c^ ^]^	50	C_7_F_8_	218 [3]	66.9 [0.49]
3	80	DOTP(90%)	711 [1]	63.3 [0.58]
4^[^ ^d^ ^]^	80	DOTP(90%)	501 [1]	63.8 [0.57]
5^[^ ^e^ ^]^	80	DOTP(90%)	447 [1]	61.3 [0.63]
6	95	DOTP(90%)	1085 [1]	60.0 [0.67]
7^[^ ^f^ ^]^	110	DOTP(90%)	687 [0.5]	57.7 [0.73]

^[a]^ (10.24 × 10^−3^ mmol (L:Rh 2:1)) by stirring at 20 bar CO/H_2_ at 105 °C (1.5 hours), in the reaction solvent (DOTP (DOTP = Dioctyl‐terephthalate) (90%) = 18 mL DOTP + 2 mL toluene) and then increasing or decreasing the temperature to the required temperature prior to running reaction at time specified using propene/CO/H_2_ in 1:4.5:4.5 ratio (20 bar initial pressure). Rh concentration = 2.52 × 10^−4^ mol dm^−3^. Product determined by GC using 1‐methylnaphthalene as an internal standard;

^[b]^ Reaction time 3 hours. Average TOF over 3 h = 47;

^[c]^ Reaction time 3 hours. Average TOF over 3 h = 73;

^[d]^ L:Rh 10:1;

^[e]^ 10 bar N_2_ and 10 bar propene/CO/H_2_ in 1:4.5:4.5 (half amount of alkene present and lower initial partial pressure of CO);

^[f]^ Reaction time 0.5 hours. Average TOF = 1374.

**Scheme 3:**
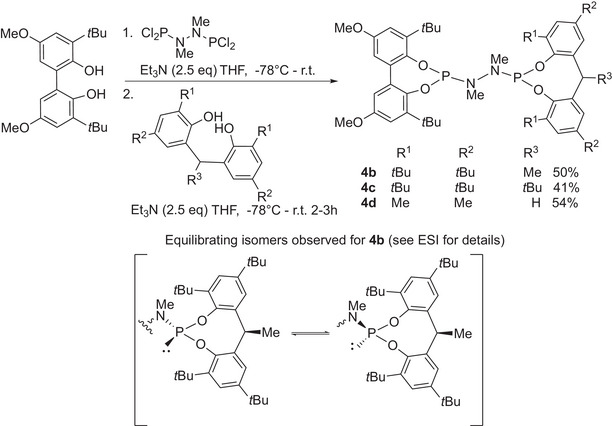
Synthesis of asymmetrically substituted *bis*‐phosphoramidites.

Two asymmetrically substituted analogues of **4a** were also prepared, as shown in Scheme 3. Ligand **4b** and bulkier **4c** were both also stable molecules and exist as two isomers. This isomerization concerns the relative orientation of the C(H)‐methyl or *t*Bu groups and the phosphorus lone pair in the eight‐membered phosphacyclic ring. The spectrum of ligand **4b** in solution (C_6_D_6_) showed a marked broadness of the ^31^P{^1^H} NMR. There are four ^31^P{^1^H} resonances in total for the two isomers. In the ^1^H NMR, the CH that bridges the aromatics is broad, consistent with a mixture of isomers that exchange under equilibrium conditions. Variable temperature NMR experiments were conducted (discussed in the ESI, section 4), that are consistent with the explanation above, but with a further isomer, assigned as being due to *E/Z* isomerism in the N─N bond just about becoming detectable at low temperatures.

In contrast ligand **4c** (with a *t*Bu group in the carbon bridge instead of a methyl group as in the case of **4b**) was prepared as a 20:1 mixture of isomers that gave much sharper resonances and, in fact, the major isomer could be separated by chromatography on SiO_2_. The minor isomer was not isolated.

Heating the major isomer of **4c** at 110 °C in toluene for 2 hours did not show the formation of any amount of the minor isomer (as monitored by ^31^P{^1^H} NMR), showing there is no exchange between **4c** isomers in solution. To further confirm that the isomers were a result of the substitution on the methylene bridge, one more ligand, **4d** was produced in which the methylene in the diol was unsubstituted; As expected this ligand shows only one signal for each P atom in the ^31^P{^1^H} NMR spectrum, confirming the source of the isomerism in **4b** as that described above.

Testing in the hydroformylation of propene was carried in the same way as was done for **4a** (Table 3). This shows that these ligands are not able to deliver such low *n:iso* ratio in Rh catalyzed hydroformylation of propene. However, there is an interesting feature of *iso* selectivity improving at higher temperature with the catalysts formed from **4b**. The dogma in the literature on bidentate ligands, is that, if any effect is observed, higher temperatures should lead to small increases in *n*‐selectivity. This is ascribed to the temperature for the branched Rh‐*iso‐*alkyl to undergo *beta*‐hydride elimination being lower than that of the linear Rh‐*n*‐alkyl intermediate.^[^ ^30^, ^31^ ^]^ Catalyst systems that have flexible *n:iso* ratio are actually of some industrial importance, since market needs change all the time.^[^ ^18^ ^]^ The % *iso‐*butanal varies between 40.5% (Table 3, entry 3) and 55.6% (Table 3, entry 9), and hence includes some relatively low *n:iso* ratio at high temperatures. Rh catalysts derived from ligand **4c** (major isomer) in contrast, do not show this phenomenon of decreasing *n:iso* ratio at higher temperature. The Rh/**4c** catalyst gives results indicating stability issues in that the average TOF over 1 hour does not increase when temperature is increased from 95 °C to 110 °C, but does increase when a large excess of ligand is employed. Little flexibility in the *n:iso* ratio with temperature changes was observed with ligand **4d**, lacking substitution in the methylene bridge (Table 3, entry 15–17). These observations, where only the ligand containing an interconverting mixture of isomers exhibits flexibility in *n:iso* ratio, suggest the presence of an isomeric catalyst might be relevant to this effect. Catalysts derived from **4a** are the most noteworthy overall due to the very unusually low *n:iso* ratio, but the phenomena observed with Rh/**4b** is unusual.

**Table 3:** Hydroformylation of propene using Rh catalysts derived from *bis*‐phosphoramidite ligands **4b‐4d**.

Entry^[^ ^a^ ^]^	Ligand	T [°C]	P_N2_^[^ ^c^ ^]^	TON 1hour	% Branched [*n:iso*]
1	**4b**	80	0	205	41.0 [1.43]
2^[^ ^b^ ^]^	**4b**	80	0	163	42.6 [1.35]
3	**4b**	80	10	192	40.5 [1.47]
4	**4b**	95	0	690	43.1 [1.32]
5	**4b**	95	10	469	50.8 [0.97]
6	**4b**	110	0	1197	49.2 [1.03]
7^[^ ^b^ ^]^	**4b**	110	0	788	49.4 [1.02]
8	**4b**	110	10	651	51.0 [0.96]
9	**4b**	110	15	290	55.6 [0.80]
10	**4c**	80	0	601	52.8 [0.89]
11^[^ ^b^ ^]^	**4c**	80	0	1405	49.4 [1.02]
12	**4c**	80	10	76	55.6 [0.80]
13	**4c**	95	0	1190	54.2 [0.85]
14	**4c**	110	0	884	54.5 [0.83]
15	**4d**	80	0	111	52.9 [0.89]
16	**4d**	95	0	374	53.8 [0.86]
17	**4d**	110	0	926	55.4 [0.81]

^[a]^ Catalyst preformed from [Rh(acac)(CO)_2_] (5.12 × 10^−3^ mmol) and ligand (10.24 × 10^−3^ mmol (L:Rh 2:1)) by stirring at 20 bar CO/H_2_ at 105 °C, 15 minutes (**4b**) and 80 °C, 50 minutes (**4c**) in DOTP (90%) (18 mL + 2 mL toluene) and then increasing or decreasing the temperature to the required temperature prior to running reaction at time specified using propene/CO/H_2_ in 1:4.5:4.5 ratio (20 bar initial pressure). Rh concentration = 2.52 × 10^−4^ mol dm^−3^. Product determined by GC using 1‐methylnaphthalene as an internal standard;

^[b]^ L:Rh 10:1;

^[c]^ The autoclave was filled with the corresponding pressure of N_2_ and then top up to total initial pressure of 20 bar with propene/CO/H_2_ in 1:4.5:4.5 (reduced amount of alkene present and lower initial partial pressure of CO).

**Figure 2:**
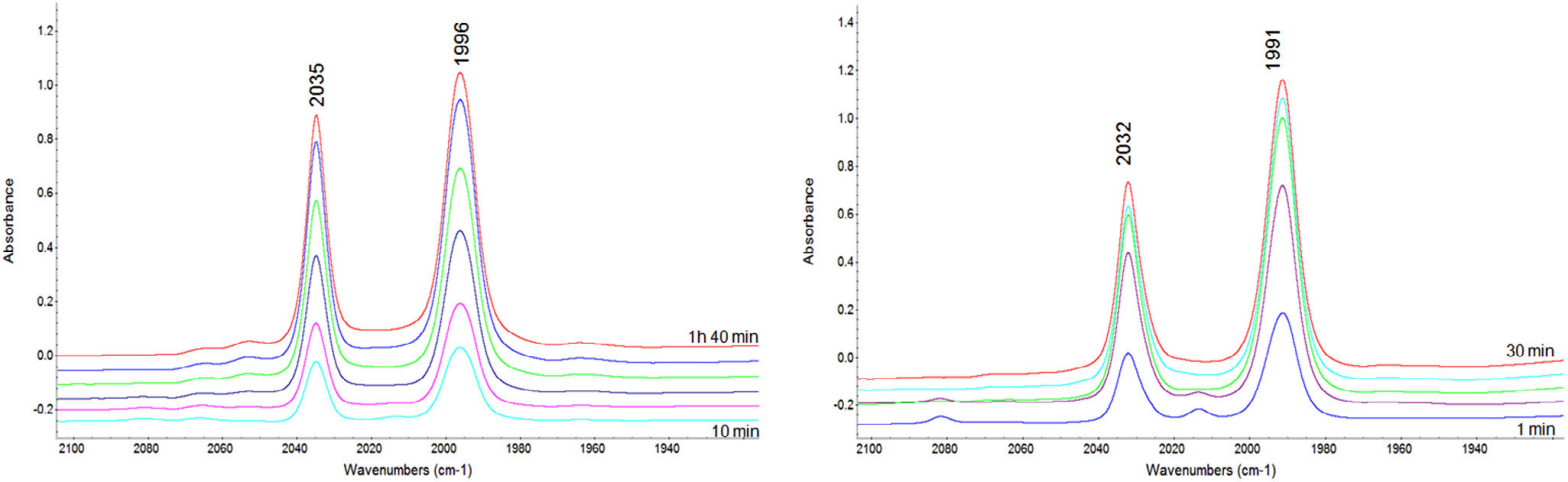
Left: HPIR spectra of [Rh(**4a**)(CO)_2_H]. Conditions: Rh:L = 1:1.25 (CRh 1 mM in dodecane), T = 105 °C, P = 20 bar, CO:H_2_ 1:1. Full activation achieved after 1.5 hours. Right: HPIR spectra of [Rh(**4b**)(CO)_2_H]. Conditions: Rh:L = 1:1.25 (CRh 1 mM in dodecane), T = 105 °C, P = 20 bar, CO:H_2_ 1:1. Full activation achieved after 20 minutes.

To understand more about these catalysts, studies on the metal complexes of these ligands were carried out. First, the time taken to form the catalyst and whether this was a single species was investigated using in‐situ HPIR spectroscopy. We have previously found that Bobphos, for example, forms an equatorial–axial coordinated catalyst, [Rh(Bobphos)(CO)_2_H] within 5 minutes at 90 °C.^[^ ^32^ ^]^ This estimate of formation time has been applied for dozens of ligands in our equipment and is the point at which the intensity of the IR bands reaches its maximum level. The spectra observed for [Rh(**4a**)(CO)_2_H] and for [Rh(**4b**)(CO)_2_H] as they form are shown in Figure 2. In both cases, two main bands of an asymmetric nature are observed at 2035 and 1996 cm^−1^ for [Rh(**4a**)(CO)_2_H] and 2032 and 1991 cm^−1^ for [Rh(**4b**)(CO)_2_H]. The nature of these bands was consistent with an equatorial–axial coordination mode for both ligands. The time needed to achieve full precatalyst formation, measured as the time needed to obtain a HPIR spectrum where the intensity of the bands observed for the complex formed remain unchanged, is especially long for the complex formed from ligand **4a**, measured at around 1.5 hours, which is amongst the longest activation time observed by us for any bidentate ligand. In contrast, precatalyst formation for **4b** was deemed achieved after 30 minutes. The same study at 105 °C was performed for ligand **4c** (see ESI, section 15, Figure S23). The inclusion of the even bulkier diol in the ligand led to the formation of Rh clusters after only 5 minutes, that is, strong bands observed at 2073 and 1821 cm^−1^ that can be assigned to the presence of [Rh_6_(CO)_16_].^[^ ^10^ ^]^ Together with the formation of Rh clusters, two bands at 2031 and 1992 cm^−1^ were also observed, that could be assigned to the formation of the expected [Rh(**4c**)(CO)_2_H]complex coordinating in an equatorial–axial fashion in the same way as ligands **4a** and **4b**. Another two bands are formed over‐time at 2039 and 2009 cm^−1^. Such behavior suggests overall that catalysts derived from **4c** are not especially stable, although we note the relatively clean spectra seemingly consisting of several discrete species differs from what is generally observed when ligands fall apart (many IR bands).

For **4a**, NMR spectroscopy was also used to structurally characterize the catalyst resting state. Precatalyst [Rh(**4a**)(CO)_2_H] was formed under syngas and analysed by NMR spectroscopy at ambient temperature (see ESI, section 9). The spectra consist of a single set of signals, in agreement with the presence of a single complex in solution or a fast equilibrium among several species. The magnitude of ^2^ *J*_P‐H_ (phosphoramidite) coupling of 109 Hz is smaller compared to that of [Rh(*S*,*S*‐Ph‐BPE)(CO)_2_H)] (^2^ *J*_P‐H_ = 132 Hz at low temperature),^[^ ^33^ ^]^ but is much larger than the coupling expected if both phosphorous atoms were in a *cis* position relative to the hydride, implying the formation of an equatorial‐axial complex where both phosphoramidite atoms can interconvert.^[^ ^9^, ^15^ ^]^

Further experiments using NMR spectroscopy were also used to assess the stability of resting state [Rh(**4a**)(CO)_2_H] to heat. We prepared the complex and heated in *n*‐dodecane at 90 °C and 20 bar of syngas for seven days (ESI, section 10). After 1 week, a precipitate was found. A sample of the solution was taken via syringe, introduced in a NMR tube previously purged with syngas and analysed by ^1^H NMR and ^31^P NMR using a benzene‐d6 capillary. In the solution, no complex or any other major P‐containing species was detected. Remaining *n*‐dodecane was then removed from the precipitate via syringe and then the precipitate was dissolved in CD_2_Cl_2_ (previously degassed with syngas). The ^1^H and ^31^P NMR showed the presence of the desired complex [Rh(**4a**)(CO)_2_H] in excellent purity even after a whole week (See ESI, section 10, Figure S14–S17).

**Scheme 4:**
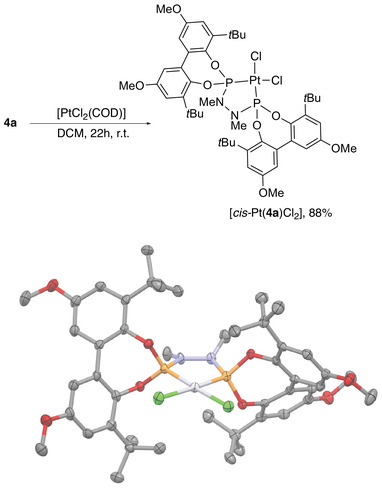
Synthesis and X‐ray crystal structure of [cis‐Pt(4a)Cl_2_] with thermal ellipsoids shown at 50% probability. Carbon atoms shown grey, oxygen red, phosphorus orange, chlorine green, and platinum light grey). One independent complex shown with hydrogen atoms, dichloromethane solvates and minor component of disorder omitted for clarity.

Due to difficulty growing crystals suitable for crystallography of the active precatalysts, platinum complexes were synthesized with an aim to obtain crystals for X‐ray crystal structure determination. For ligand **4a**, the reaction with [PtCl_2_(COD)] occurred cleanly and the expected Pt complex, [*cis*‐Pt(**4a**)Cl_2_] was isolated in 88% yield (Scheme 4). The magnitude of ^1^ *J*_P‐Pt_ varies depending on the type of phosphorous ligand employed with phosphites and phosphoramidites showing larger coupling constants than phosphines. For any given ligand type, the coupling constant is around 80% larger if P is *trans* to chloride as opposed to another phosphorous ligand due to their relative positions in the spectrochemical series. The value shown here of 5582 Hz can be considered diagnostic for the expected complex with *cis* geometry. It is also a similar value of ^1^ *J*_P‐Pt_ to another hydrazine‐*bis*‐phosphoramidite in the literature (5408 Hz).^[^ ^34^, ^35^ ^]^

Crystals of [*cis*‐Pt(**4a**)Cl_2_]·2CHCl_3_ suitable for X‐ray crystallography were obtained from the diffusion of hexane into a chloroform solution of [*cis*‐Pt(**4a**)Cl_2_]. The structure (Scheme 4) contains two independent molecules of the complex in the asymmetric unit, which show near identical conformation. The platinum(II) centres are square planar (τ and τ’ <0.07) and **4a** coordinates in the expected chelating *P,P’* manner, with the Pt‐P bond lengths (Pt‐P 2.1707(14) – 2.1832(13) Å) similar to a previous structure of a *bis*‐phosphoramidite of this type.^[^ ^34^, ^36^ ^]^ The nitrogen atoms of the hydrazine backbone are planar (sum of angles at N 356.2(11) – 359.3(11)°), as are most of the many compounds with P─N bonds, since they exhibit sp^2^ hybridization. However, X‐ray structures of some ligands with hydrazine backbones have been found to exhibit one planar and one more tetrahedral N atom.^[^ ^34^, ^35^, ^36^ ^]^ The relative energy gains from different conformation or N geometries are likely to be quite finely balanced. The torsion angles across the hydrazine backbones (C─N─N─C 53.6(6) and 42.6(6)°, respectively) show a slightly more eclipsed angle than a gauche conformation, and as such, it can be envisaged there is some strain in the PtPNNP ring system.

**Scheme 5:**
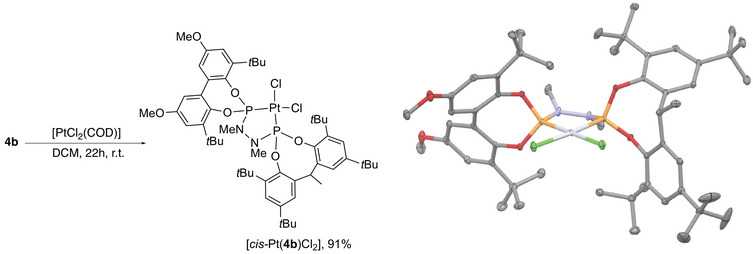
Synthesis and X‐ray crystal structure of [*cis*‐Pt(**4b**)Cl_2_] with thermal ellipsoids shown at 50% probability. Carbon atoms shown grey, oxygen red, phosphorus orange, chlorine green, and platinum light grey). Hydrogen atoms and dichloromethane solvates omitted for clarity.

When the mixture of isomers of **4b** was reacted with [PtCl_2_(COD)], the formation of what appears to be an isomerically pure *cis*‐complex of platinum, [*cis*‐Pt(**4b**)Cl_2_] was observed with 91% yield (Scheme 5). The spectroscopic features are broadly similar to [*cis*‐Pt(**4a**)Cl_2_]. Additionally, there is only one reasonably sharp resonance in the ^1^H NMR spectrum for the C─H and C‐methyl groups in the methylene‐bridged diol unit, in contrast to the free ligand where a broad resonance is observed for each isomer. These reactions were repeated with sampling of the reaction after 5 and 75 minutes to see if preferential coordination of one isomer of the ligand can be observed (see ESI, section 4.1 and 6.3). A similar experiment was also carried out using a two‐fold excess of the ligand. These experiments showed a single isomer of Pt complex forming, but no change in the isomeric ratio of the starting ligand, consistent with the NMR evidence that the isomers of free ligand interconvert rapidly (see ESI, section 6.3). Heating [*cis*‐Pt(**4b**)Cl_2_] at 110 °C in toluene for two hours did not lead to any changes in the NMR spectra of the complex; it appears that no equilibration of ligand isomers occurs once the ligand is coordinated to platinum(II). Crystals of [*cis*‐Pt(**4b**)Cl_2_]·2.5CH_2_Cl_2_ suitable for X‐ray crystallography were obtained from the diffusion of hexane into a dichloromethane solution of [*cis*‐Pt(**4b**)Cl_2_]. The complex shows similar coordination geometry to that seen in [*cis*‐Pt(**4a**)Cl_2_]; the platinum(II) centre is square planar (τ and τ’ <0.06) and **4b** coordinates in the expected chelating *P,P’* manner, with the Pt‐P bond lengths (Pt‐P 2.1764(6) and 2.1934(53) Å) in the same range. The N atoms of the hydrazine backbone are again planar (sum of angles at N 355.2(4) and 357.5(4)°, respectively) but adopt a more typically gauche conformation (C─N─N─C 62.2(2)°).

Regarding the unusual behavior of **4b** in hydroformylation where lower *n:iso* ratios were observed at higher temperatures, it is important to note the effect is subtle, and subtle effects can often not be detected spectroscopically. Some observations can be made; we have observed that this is a reversible effect; if the experiments are carried out with a catalyst that is aged at 110 °C in hexanal (see ESI, section 11) before carrying out the hydroformylation at 80 °C, essentially the same results are observed as a normal experiment at 80 °C. Thermal decomposition of **4b** to another catalyst therefore appears ruled out. Whilst the coordination studies show that the free ligand interconverts between its two isomers at room temperature, there is evidence that the Pt complex does not equilibrate, which is entirely expected. If the two conformational isomers were relatively similar in thermodynamic stability, but one was significantly favored to coordinate kinetically, it is possible that the rhodium complex of the other isomer could only be present at higher temperature when sufficient energy to overcome the barrier to coordination for the kinetically disfavoured ligand isomer was present. This seems more likely than interconversion within the eight‐membered ring on the rhodium. The minor isomer could give lower *n:iso* ratios than the dominant isomer at room temperature. The HPIR evidence does not provide evidence for this since only one main species is observed at high temperatures. However, IR bands are often hidden underneath the major species, and the likely electronic similarities of the two isomers make it likely that each isomer would display very similar IR bands. There is an additional possibility for the minor component to be a more active catalyst and hence disproportionately influence selectivity also makes a lack of HPIR evidence not surprising. The most likely explanation is that minor undetectable species becomes present at higher temperatures and alters the selectivity.

Whilst this project was focused upon propene hydroformylation, a very simply‐made achiral catalyst that would give predominantly branched isomers for other terminal alkenes could be of use to synthetic chemists. Whilst, commercially available Bobphos ligand fulfils this role, it is enantiomerically pure, which may not be needed, or even desired, in racemic aldehyde synthesis. We therefore examined the use of the EasyDiPhos ligand **4a** in Rh‐catalyzed hydroformylation of a selection of terminal alkenes. Some of these are entirely unbiased alkenes that normally favor the linear product. Others have polar groups that can help move the regioselectivity toward branched aldehydes products. The screening conditions chosen aim to give the best chance for high branched regioselectivity, with low temperatures, and the unusual fluorinated solvent choice that has been beneficial for two sets of quite structurally different substrates.^[^ ^16^, ^17^ ^]^ We note that the fluorinated solvents were not as compatible with these catalysts due to low solubility at low temperature. Probably the most interesting regioselectivity levels are in allyl benzene hydroformylation. For example, in Ref [13] where 84:16 branched selectivity was reported for allyl phthalimide hydroformylation, allyl benzene gave only 42% regioselectivity, and various other catalysts give around a 1:1 ratio. A few experiments were carried out to clarify the best conditions for best regioselectivity, with slightly improved results observed as ligand to Rh ratio increases from 1.25 to 3. This tendency of better regioselectivity with higher L:Rh ratio has been observed before with Rh/Bobphos catalysts needing a reasonably large excess of ligand to optimize regioselectivity.^[^ ^16^, ^28^ ^]^ The regioselectivity of around 3.8:1 (79% b) is slightly below that observed with the enantiomerically pure Rh/Bobphos catalyst (∼6:1), (86% b)^[^ ^16^ ^]^ or encapsulated catalysts,^[^ ^24^ ^]^ but the fact that ligand **4a** is so easily made could make this a useful option for racemic branched aldehyde synthesis. The branched isomer would be expected to be the major product for allyl cyanide; the level of regioselectivity, whilst better than many catalysts, is slightly lower than the very best reported.^[^ ^37^ ^]^ The regioselectivity for allyl phthalimide hydroformylation is slightly lower than the best previously reported (81:19 versus 84:16^[^ ^38^ ^]^). Oct‐1‐ene gives lower regioselectivity than propene. We conclude that Rh/**4a** also gives unusual regioselectivity favoring the branched isomer for other terminal alkene substrates. Although there are no dramatic improvements to the state‐of‐the‐art, it should be noted that this is a readily available achiral ligand and might prove useful relative to more complex chiral ligands when racemic products are sought (Table 4).

**Table 4:** Hydroformylation of alkenes of type RCH_2_CH═CH_2_ using Rh/4a catalyst.^[^ ^a^ ^]^

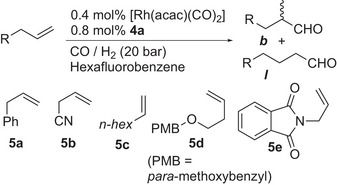				
Entry^[^ ^a^ ^]^	Substrate	Conv.^[^ ^b^ ^]^	Conv. Aldehydes^[^ ^b^ ^]^	% Branched [b:l]
1	**5a** ^[^ ^c^ ^]^	>99	90 [76]	72 [2.6:1]
2	5a	>99	94 [87]	78 [3.5:1]
3	**5a** ^[^ ^d^ ^]^	>99	95 [87]	79 [3.8:1]
4	**5a** ^[^ ^e^ ^]^	>99	98	78 [3.6:1]
4	**5b** ^[^ ^f^ ^]^	27	26	94 [15.5:1]
5	**5b** ^[^ ^g^ ^]^	>99	97 [73]	91 [10.4: 1]
8	5c	>99	94 [71]	65 [1.8:1]
9	5d	>99	92 [85]	71 [2.5:1]
11	5e	>99	96 [85]	81 [4.2:1]

^[a]^ 0.4 mol% [Rh(acac)(CO)_2_] and 0.8 mol% **4a** were stirred at 20 bar syngas at 90 °C for 5 hours in C_6_F_6_ (2 mL), prior to running the reaction at 30 °C and 20 bar initial syngas pressure for 89 h;

^[b]^ % Conversion = terminal alkene consumed; % products = linear and branched aldehydes determined by ^1^H NMR spectroscopy using 1‐methylnaphthalene as internal standard; isolated yield of aldehyde isomers;

^[c]^ 0.5 mol% of **4a**. Product isolated was alcohol after reduction of aldehyde with NaBH_4._

^[d]^ 1.2 mol% of **4a**;

^[e]^ 1.2 mol% of **4a**. 40 °C, 8 bar, 24 hours;

^[f]^ 18 h;

^[g]^ 60 °C, 42 hours.

## 3 Conclusion

Two ligand classes that can be made from commercially available chemicals in one synthetic operation have been synthesized and their use in rhodium catalyzed propene hydroformylation investigated. Whilst the C1‐symmetric *bis*‐phosphonites discussed initially validated the design principles, almost as good branched selectivity can be realized with even simpler C2‐symmetric analogues. Whilst the phosphonite ligands were very moisture sensitive, *bis*‐phosphoramidites showed significant stability to water and air to enable standard manipulations and application in catalysis. They are so easy to prepare, we suggest the ligand family name, EasyDiPhos. They also gave thermally robust catalysts that were stable for long periods using elevated reaction temperatures. The latter was verified by a combination of HPIR experiments and NMR monitoring of the catalyst resting state. The *bis*‐phosphoramidite ligands form [Rh(L)(CO)_2_H] catalysts more slowly than many of the bidentate ligands we have studied in the past, and a suitable catalyst activation phase needs to be considered if the catalysts are to be used. Once the ligands have coordinated, they form a single resting state, except in the case of the very bulky example **4c**. Catalysts that give unusually high *iso*‐selectivity in terminal alkene hydroformylation have been reported before, but still are generally quite complicated ligands that an end‐user may not be willing or able to synthesize. A quick examination of a selection of other terminal alkenes reveals the level of selectivity for allylbenzene hydroformylation to be unusually high, and hence this represents one example of a class of racemic branched aldehydes that can be made more readily, once catalyst synthesis is taken into account, using the new ligands. Since the catalysts do not show an extremely strong preference for the branched isomer, it is likely to be difficult to uncover the reasons behind the selectivity. However, from an empirical point of view, what are arguably the best two families of catalysts for making the more branched aldehydes from unbiased terminal alkyl alkenes, both exclusively form equatorial‐axial coordinated [Rh(L)(CO)_2_H] complexes. It is possible some of the space around the Rh‐propyl active site in Rh/ **4a** catalysts is filled in a quite similar fashion to Rh/Bobphos catalysts. The examination of other chelating narrow bite‐angle *bis*‐phosphorous ligands may turn out to offer further opportunities to further refining performance in branched‐selective hydroformylation. The ligand class reported here might prove generally useful in catalysis. There has been significant interest in modular, rapidly made phosphoramidite ligands^[^ ^39^, ^40^ ^]^ and the ligands reported here are stable, easy to make and have demonstrated unusual features in the reactions studied here. A previous paper^[^ ^35^ ^]^ reported somewhat similar N‐N backboned ligands with chiral diols for asymmetric hydrogenation; combined with the observations reported here, the EasyDiPhos family seems to have many desirable features for regioselective or enantioselective catalysis. It is suggested that this ligand class has been underdeveloped, and hoped this article will spur further efforts.

## 4 Experimental Section

4.1

All reactions were performed under an inert atmosphere of nitrogen or argon using standard Schlenk techniques, unless otherwise stated. All glassware used was flame‐dried. Dry and degassed solvents were obtained from a solvent still or SPS solvent purification system. Commercially purchased anhydrous solvents were degassed before use by the freeze‐pump‐thaw method or by purging with inert gas. Triethylamine and CDCl_3_ were dried and degassed before use. All chemicals, unless specified were purchased commercially and used as received. CO/H_2_ and propylene/CO/H_2_ (10/45/45%) were obtained premixed from BOC. Flash column chromatography was performed using Merck Geduran Si 60 (40–63 µm) silica gel. Thin layer chromatographic (TLC) analyses were carried out using POLYGRAM SIL G/UV254 or POLYGRAM ALOX N/UV254 plastic plates. TLC plates were visualized using a UV visualizer or stained using potassium permanganate dip followed by gentle heating.

NMR spectra were recorded on a Bruker Avance 300, 400 or 500 MHz instrument. Proton chemical shifts are referenced to internal residual solvent protons. Carbon chemical shifts are referenced to the carbon signal of the deuterated solvent. Signal multiplicities are given as s (singlet), d (doublet), t (triplet), q (quartet), m (multiplet), or a combination of the above. Where appropriate coupling constants (*J*) are quoted in Hz and are reported to the nearest 0.1 Hz. All spectra were recorded at r.t. (unless otherwise stated) and the solvent for a particular spectrum is given in parentheses. NMR of compounds containing phosphorus were recorded under an inert atmosphere in dry and degassed solvent. Gas chromatography was performed on an Agilent Technologies 7820A machine. Mass spectrometry was performed on a Micromass GCT spectrometer, Micromass LCT spectrometer, Waters ZQ4000, Thermofisher LTQ Orbitrap XL or Finnigan MAT 900 XLT instruments. X‐ray diffraction data for compounds [*cis*‐Pt(**4a**)Cl_2_]·2CHCl_3_ and [*cis*‐Pt(**4b**)Cl_2_]·2.5CH_2_Cl_2_ were collected using a Rigaku FR‐X Ultrahigh Brilliance Microfocus RA generator/confocal optics with XtaLAB P200 diffractometer [Mo Kα radiation (*λ* = 0.71073 Å)]. CCDC 2429273 and CCDC 2429274 contain the supplementary crystallographic data for this paper. These data can be obtained free of charge from The Cambridge Crystallographic Data Centre via www.ccdc.cam.ac.uk/structures. Further crystallographic details can be found in the supporting information.

### 1,2‐*bis*(4,8‐di‐*tert*‐Butyl‐2,10‐dimethoxydibenzo[*d*,*f*][1,3,2]dioxaphosphepin‐6‐yl)‐1,2‐dimethylhydrazine, 4a

To a stirred solution of 1,2‐*bis*(dichlorophosphaneyl)‐1,2‐dimethylhydrazine (0.166 g, 0.63 mmol) in THF (3 mL) at −78 °C, was added a solution of 3,3′‐di‐*tert*‐butyl‐5,5′‐dimethoxy‐[1,1′‐biphenyl]‐2,2′‐diol (0.454 g, 1.27 mmol) in THF (5 mL) slowly via syringe. This was followed by Et_3_N (0.386 mL, 2.77 mmol) also added via syringe. The reaction was then allowed to warm to −45 °C while stirring for 1 hour, then the solution was taken out of the cold bath and stirred for a further 90 minutes at room temperature. The reaction was filtered under an argon atmosphere and concentrated in *vacuo*. The resulting solid was purified under air by flash chromatography on silica gel (5:1 petrol (40–60 °C):EtOAc) affording the desired product **4a** (0.386 g, 0.463 mmol, 74%) as a white solid. ^1^H NMR (C_6_D_6_, 500 MHz) δ 7.20 (4H, br d, *J =* 2.6 Hz, ArCH), 6.74 (4H, br d, *J =* 2.6 Hz, ArCH), 3.36 (12H, s, 4 x OCH_3_), 2.76 (6H, s, 2 x NCH_3_), 1.62 (18H, s, 2 x C(CH_3_)_3_), 1.53 (18H, s, 2 x C(CH_3_)_3_). ^13^C NMR (C_6_D_6_, 126 MHz) δ 155.75–155.69 (4 x ArC), 143.61 (2 x ArC), 143.31 (2 x ArC), 142.74 (2 x ArC), 142.16 (2 x ArC), 134.06 (2 x ArC), 133.44 (2 x ArC), 114.61 (4 x ArCH), 112.83 (4 x ArCH), 54.74 (4 x OCH_3_), 35.64 (2 x NCH_3_), 35.50 (2 x *C*(CH_3_)_3_), 35.18 (2 x *C*(CH_3_)_3_), 30.76 (4 x C(*C*H_3_)_3_). ^31^P{^1^H} NMR (C_6_D_6_, 202 MHz) δ 144.0 (s). HRMS (ES^+^) [MH]^+^ m/z: 833.4028 found, C_46_H_63_O_8_N_2_P_2_ requires 833.4054.

### General Procedure for Rhodium‐Catalyzed Hydroformylation of Propene

CAUTION: Carbon monoxide and hydrogen mixtures are toxic and highly flammable. Experiments of this nature should only be carried out in a well‐ventilated fumehood, by suitably trained chemists. CO detection instruments should be used to ensure there are no leaks. Hydroformylation reactions of propene were performed in a Parr 4590 Micro Reactor fitted with a gas entrainment stirrer; comprising of holes which gives better gas dispersion throughout the reaction mixture. The vessel had a volume capacity of 0.1 L, an overhead stirrer with gas entrainment head (set to 1000 r.p.m.), temperature controls, pressure gauge and the ability to be connected to a gas cylinder.

Ligand (10.24 µmol (Rh:L 1:2)) was added to a Schlenk tube, which was then purged with nitrogen (or argon). The internal standard 1‐methylnaphthalene (0.1 mL) was then added. The mixture was dissolved in a stock solution of [Rh(acac)(CO)_2_] in toluene (2 mg/mL, 0.65 mL, 5.12 µmol of [Rh(acac)(CO)_2_]), followed by the addition of the designated solvent (19.35 mL, for DOTP(90%) 1.35 ml toluene, and 18 mL of DOTP). The solution was transferred via syringe to the pressure vessel (which had been purged with CO/H_2_) through the injection port. CO/H_2_(1:1) (20 bar) was added and the heating jacket set to the desired temperature while stirring. Once the desired temperature was reached, the reaction was stirred for the required time to fully activate the catalyst. Then pressure was slowly released and repressurised with propene/CO/H_2_. The reaction was then run for the time specified in the tables. After this time, stirring was stopped and the reaction was cooled by placing the vessel in a basin of cold water. The pressure was released, and the crude sample was analysed immediately by GC (in toluene). The GC method was run on a HP‐5 Agilent column; with length 30 m, diameter 0.250 mm and film 0.25 µm. The oven was initially held at 25 °C for 6 minutes, and then increased to 60 °C at a rate of 10 °C per minute. The ramp was then increased to 20 °C per minute until the temperature reached 300 °C. The products could be identified with the following retention times; *iso*‐butyraldehyde (1.02 min); *n*‐butyraldehyde (1.15 min) and 1‐methylnaphthalene (13.50 min). The GC was calibrated for propene hydroformylation using (1‐methylnaphthalene) as an internal standard. Both the linear (*n‐*butyraldehyde) and branched (*iso*‐butyraldehyde) products were calibrated against the internal standard and against each other.

## Supporting Information

The supporting information contains detailed procedures and NMR spectra for all compounds. This includes some further discussion of NMR experiments. The authors have cited additional references within the Supporting Information.^[^ ^41^, ^42^, ^43^, ^44^, ^45^, ^46^, ^47^, ^48^, ^49^, ^50^, ^51^, ^52^, ^53^, ^54^ ^]^ Crude NMR files are also available.^[^ ^55^ ^]^

## Conflict of Interests

The authors declare no conflict of interest.

## Supporting information



Supporting Information

## Data Availability

The research data underpinning this publication can be accessed at https://doi.org/10.17630/d8cccd53‐5f35‐4bad‐a86b‐037658c7c8f7.
