# Supplementary material for: A Very Readily Prepared Ligand for Rhodium Catalyzed Propene Hydroformylation
Source: Chemistry. 2025 Jun 3;31(34):e202500917. doi: 10.1002/chem.202500917 (PMC12172607; doi:10.1002/chem.202500917)
Supplement: Supplementary file 1 — Supporting Information [file CHEM-31-e202500917-s001.pdf]

Supporting Information for:

# A Very Readily Prepared Ligand for Rhodium Catalysed Propene Hydroformylation.

José A. Fuentes,[a] David B. Cordes,[a] Aidan McKay,[a] Mesfin E. Janka,[b] and Matthew L. Clarke*[a]

[a] Dr. J. A. Fuentes, Dr. D. Cordes, Dr. A. McKay, Prof., Prof. M. L. Clarke

EaStCHEM School of Chemistry, University of St Andrews,

Purdie Building, North Haugh, St Andrews, KY16 9ST, United Kingdom

E-mail: mc28@st-andrews.ac.uk

[b] Dr Mesfin E. Janka

Eastman Chemical Company, 200 South Wilcox Drive, Kingsport, Tennessee, 37660, USA

**Table of Contents**

1. General Information.

1.1. High pressure infrared spectroscopy

2. General procedures.

2.1 General Procedure for rhodium-catalysed hydroformylation of propene.

2.2 General procedure for monitoring the complexation of ligands using HPIR spectroscopy.

2.3. General procedure for rhodium catalysed hydroformylation of alkenes.

3. Synthesis of phosphonites **3b** and **3c**

4. Synthesis of bis-phosphoramidite ligands **4a-4d.**

4.1. Variable temperature NMR spectroscopy measurement of ligand **4b**.

5. Bis-phosphoramidite ligands**,** relative stability to moisture compared to phosphonites.

6. Preparation of Pt complexes.

6.1 Preparation of [Pt(**4a**)$Cl_2$].

6.2 Preparation of [Pt(**4b**)$Cl_2$].

6.3 NMR monitoring of the formation of [Pt(**4b**)$Cl_2$].

7. Experimental data for products from catalysis.

8. Procedure for the rhodium-catalysed hydroformylation of propene. Gas-uptake experiment using **4a** as ligand.

9. NMR spectroscopic investigation of the coordination mode of [Rh(**4a**)$(CO)_2$H].

10. NMR spectroscopic investigation of the stability under syngas at 90 °C of [Rh(**4a**)$(CO)_2$H]. One week-long experiment.

11. Propene hydroformylation using Rh/**4b**, comparing selectivity before and after ageing in the presence of an aldehyde.

12. Reaction mixture from allylbenzene hydroformylation.

13. Effect of ligand **4d** on propylene hydroformylation.

14. X-ray Crystallography.

15. HPIR spectroscopic activation study of phopshonites **3b** and **3c** and phosphoramidites **4a**-**4d** with [Rh(acac)$(CO)_2$].

16. NMR spectra.

18. References

## 1. General Information.

All reactions were performed under an inert atmosphere of nitrogen or argon using standard Schlenk techniques, unless otherwise stated. All glassware used was flame-dried. Dry and degassed solvents were obtained from a solvent still or SPS solvent purification system.

Commercially purchased anhydrous solvents were degassed before use by the freeze-pump-thaw method or by purging with inert gas. Triethylamine and $CDCl_3$ were dried and degassed before use. All chemicals, unless specified were purchased commercially and used as received. $CO/H_2$ and propylene/$CO/H_2$ (10/45/45%) were obtained pre-mixed from BOC.

NMR spectra were recorded on a Bruker Avance 300, 400 or 500 MHz instrument. Proton chemical shifts are referenced to internal residual solvent protons. Carbon chemical shifts are referenced to the carbon signal of the deuterated solvent. Signal multiplicities are given as s (singlet), d (doublet), t (triplet), q (quartet), m (multiplet) or a combination of the above. Where appropriate coupling constants ($J$) are quoted in Hz and are reported to the nearest 0.1 Hz. All spectra were recorded at r.t. (unless otherwise stated) and the solvent for a particular spectrum is given in parentheses. NMR of compounds containing phosphorus were recorded under an inert atmosphere in dry and degassed solvent.

Gas chromatography was performed on an Agilent Technologies 7820A machine.

Mass spectrometry was performed on a Micromass GCT spectrometer, Micromass LCT spectrometer, Waters ZQ4000, Thermofisher LTQ Orbitrap XL or Finnigan MAT 900 XLT instruments.

Flash column chromatography was performed using Merck Geduran Si 60 (40-63 µm) silica gel.

Thin layer chromatographic (TLC) analyses were carried out using POLYGRAM SIL G/UV254 or POLYGRAM ALOX N/UV254 plastic plates. TLC plates were visualised using a UV visualizer or stained using potassium permanganate dip followed by gentle heating.

3,3'-di-tert-butyl-5,5'-dimethoxy-[1,1'-biphenyl]-2,2'-diol[41] and 2-allylisoindoline-1,3-dione (allylphthalimide)[42] were prepared via the synthetic routes reported previously.

### 1.1. High pressure infrared spectroscopy

High pressure infrared spectroscopy was performed in a Parr high pressure IR CSTR vessel constructed from Hastelloy C, fitted with $CaF_2$ windows and rated to 275 bar. The adjustable pathlength was set to 4 mm. The high pressure IR spectra were recorded using an Avatar 360 FT-IR.

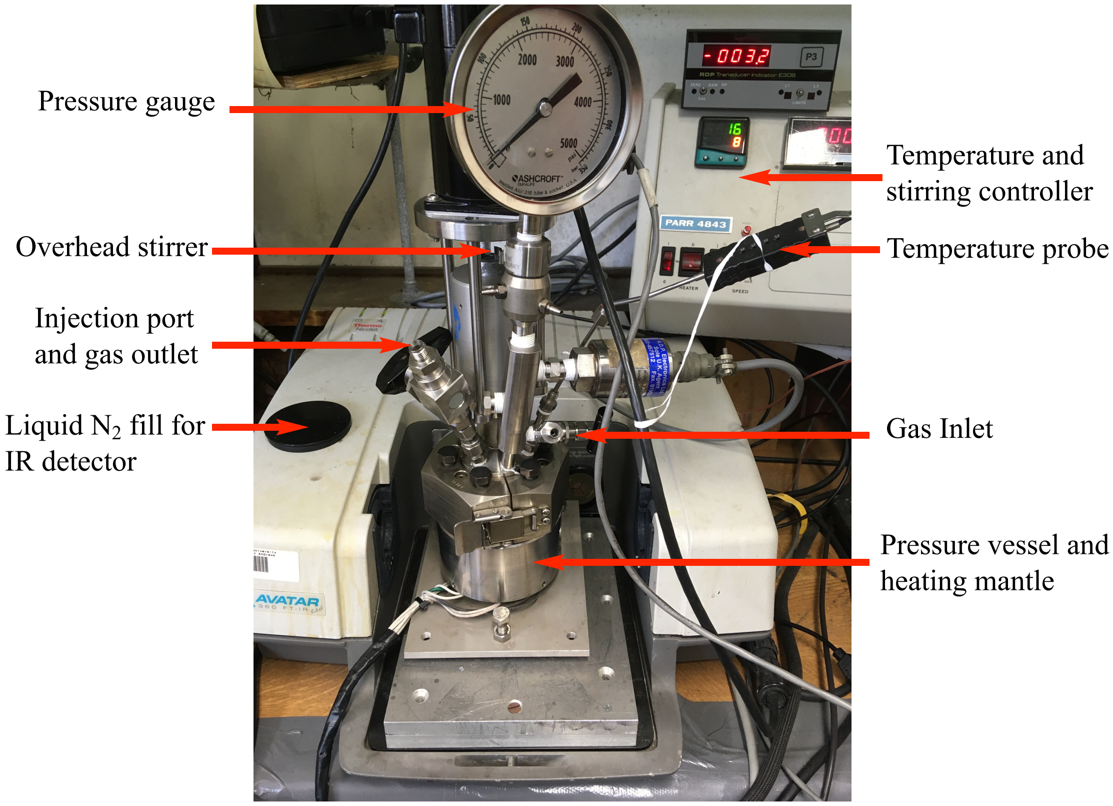


**Figure S1.** High pressure infrared spectroscopy apparatus

In reference [10], we described further aspects of validating the HPIR set up. Catalyst decomposition of a phosphine modified Rh catalyst can take many forms, some of them giving inactive Rh catalysts such as Rh metal or certain dimeric species with bridging phosphido ligands. However, it is common for the decomposed catalysts to also be some form of hydroformylation catalyst, either with an altered phosphine ligand (such as a monodentate ligand formed after cleavage of a bidentate ligand into two or more components). Another possibility is that after ligand decoordination and decomposition, the so-called unmodified Rh catalysts can be present. In reference [10], HPIR spectra of these catalysts in this HPIR set up are presented in the ESI. These show that using [$Rh_2$(hexanoate)$_4$] as precursor, the unmodified catalyst takes a cluster resting state with peaks at 2074, 2043 and 1822 $cm^{-1}$. This is consistent with previous work[43] assigning this species as [$Rh_6(CO)_{16}$]. This is one resting state for the unmodified catalyst under hydroformylation conditions. [Rh(acac)$(CO)_2$] the precursor used in these experiments, tends to remain partially intact under hydroformylation conditions (IR bands at 2082 and 2015 $cm^{-1}$), but also accompanied

by two clusters [2074, 2069, 2044 and 1888, which are assigned to [$Rh_4(CO)_{12}$)] and lower levels of [$Rh_6(CO)_{16}$] noted above.

In the stability studies of the ligands presented in this paper we did not find any evidence of the presence of [Rh(acac)$(CO)_2$], [$Rh_4(CO)_{12}$)] or [$Rh_6(CO)_{16}$] in noticeable amounts (any band in the 1888-1822 $cm^{-1}$ region would be especially easily spotted since this is an uncluttered area in the spectra).

## 2. General procedures.

### 2.1 General Procedure for rhodium-catalysed hydroformylation of propene.

Hydroformylation reactions of propene were performed in a Parr 4590 Micro Reactor fitted with a gas entrainment stirrer; comprising of holes which gives better gas dispersion throughout the reaction mixture. The vessel had a volume capacity of 0.1 L, an overhead stirrer with gas entrainment head (set to 1200 r.p.m.), temperature controls, pressure gauge and the ability to be connected to a gas cylinder.

Ligand (10.24 μmol (Rh:L 1:2)) was added to a Schlenk tube, which was then purged with nitrogen (or argon). The internal standard 1-methylnaphthalene (0.1 mL) was then added. The mixture was dissolved in a stock solution of [Rh(acac)$(CO)_2$] in toluene (2 mg/mL, 0.65 mL, 5.12 μmol of [Rh(acac)$(CO)_2$]), followed by the addition of the designated solvent (19.35 mL, for DOTP (90%) (1.35 ml toluene and 18 mL of DOTP). The solution was transferred *via* syringe to the pressure vessel (which had been purged with $CO/H_2$) through the injection port. $CO/H_2$(1:1) (20 bar) was added and the heating jacket set to the desired temperature while stirring. Once the desired temperature was reached, the reaction was stirred for the required time to fully activate the catalyst. Then pressure was slowly released and repressurised with propene/$CO/H_2$. The reaction was then run for the time specified in the tables. After this time, stirring was stopped and the reaction was cooled by placing the vessel in a basin of cold water. The pressure was released, and the crude sample was analysed immediately by GC (in toluene).
The GC method was run on a HP-5 Agilent column; with length 30 m, diameter 0.250 mm and film 0.25 μm. The oven was initially held at 25 °C for 6 minutes, and then increased to 60 °C at a rate of 10 °C per minute. The ramp was then increased to 20 °C per minute until the temperature reached 300 °C. The products could be identified with the following retention times; *iso*-butyraldehyde (1.02 min); *n*-butyraldehyde (1.15 min) and 1-methylnaphthalene (13.50 min). The GC was calibrated for propene hydroformylation using (1-methylnaphthalene) as an internal standard. Both the linear (*n*-butyraldehyde) and branched (*iso*-butyraldehyde) products were calibrated against the internal standard and against each other.

### 2.2 General procedure for monitoring the complexation of ligands using HPIR spectroscopy.

The HPIR spectroscopy pressure vessel was purged with $CO/H_2$ three times and then n-dodecane (22 mL) was injected into the pressure vessel. The heating jacket set to 90 °C (or the desired temperature) while stirring. Once the required temperature was reached, the vessel was pressurised at 20 bar of syngas and a background spectrum was recorded (1024 scans). [Rh(acac)$(CO)_2$] (0.06 mmol) and ligand (0.075 mmol

(Rh:L 1:1.25)) was added to a flame dried schlenk tube, which was then purged with nitrogen (or argon). n-dodecane (8 mL) was added to dissolve the rhodium precursor and ligand and stirred for 10 minutes. The reaction mixture was then transferred *via* syringe to the pressure vessel (which had been depresurized before addition) through the injection port, $CO/H_2$ (20 bar) was added and sampling initiated, 128 scans per spectrum. Active catalyst formation was then monitored by IR.

**2.3. General procedure for rhodium catalysed hydroformylation of alkenes.**

$[Rh(acac)(CO)_2]$ (1 mg, 0.004 mmol, 0.4 mol%) and ligand **4a** (6.7 mg, 0.008 mmol, 0.8 mol%) were added into a vial. A stirring bar was added, and the vial was sealed with a crimp cap and put under inert atmosphere (Ar). Two needles were pierced into the septum of the vial cap and the vial was placed into the autoclave. The autoclave was then purged with two vacuum/Ar cycles. Hexafluorobenzene (2 ml) was added using a syringe via an injection port equipped with a septum on the autoclave. The autoclave was then purged three times with syngas (50/50, $CO/H_2$), pressurised to 20 bar, inmersed into an oil bath preheated at 90 °C and stirred at 700 r.p.m for 5 h. After this time, the autoclave was cooled down to room temperature by partial inmersion in cold water and the pressure released to 1 atm of syngas. A solution of the corresponding alkene (1 mmol) and internal standard (1-methylnaphthalene, 50 μl) in hexafluorobenzene (2ml) was prepared and a NMR sample ($t_0$) was taken. The solution was then added using a syringe via the autoclave injection port, through the septum in the glass reaction vial. The autoclave was then pressurised back to the desired pressure (20 bar) and stirred at the required temperature at 700 r.p.m. After the desired reaction time, the pressure was slowly released, and the autoclave was opened. A small sample was taken and analysed by $^1$H NMR ($C_6D_6$) to calculate conversion and branched to linear ratio of the resulting aldehydes. In all cases, no significant amount of any isomerised alkenes or other aldehyde isomers have been detected.

### 3. Synthesis of Phosphonites 3b and 3c

**3.1** 4,8-di-*tert*-butyl-6-(2-((3a*R*,6*R*)-2,2-dimethyl-4,4,8,8-tetra(naphthalen-1-yl)tetrahydro-[1,3]dioxolo[4,5-*e*][1,3,2]dioxaphosphepin-6-yl)ethyl)-2,10-dimethoxydibenzo[*d,f*][1,3,2]dioxaphosphepine, **3b.**

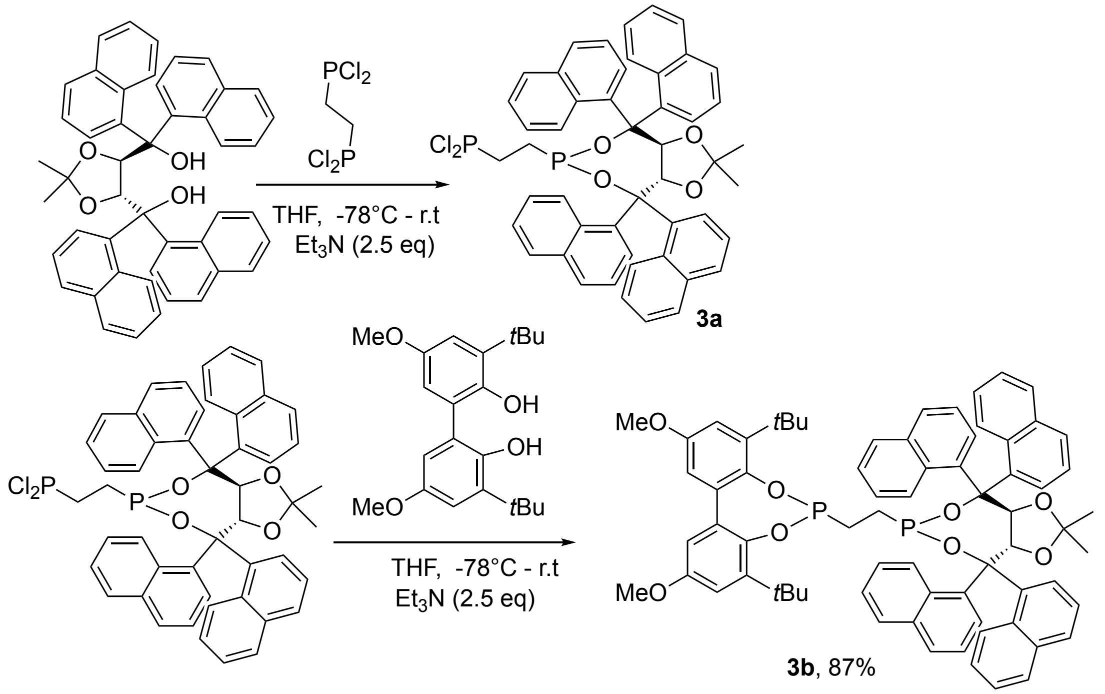


To a stirred solution of ((4*R*,5*R*)-2,2-dimethyl-1,3-dioxolane-4,5-diyl)bis(di(naphthalen-1-yl)methanol) (3.072 g, 4.65 mmol) in THF (30 mL) at –78°C, was added in one portion a solution of 1,2-bis(dichlorophosphino)ethane (1.078 g, 0.7 ml, 4.65 mmol). This was followed by $Et_3N$ (1.62 mL, 11.63 mmol) also added via syringe. The reaction was stirred at –78°C for 10 min and then allowed to warm to -50°C while stirring for 0.5 h. At that point, an NMR was taken ($C_6D_6$ capilar) that showed full conversion to the intermediate **3a**, $^{31}P\{^1H\}$ NMR (THF, $C_6D_6$, 202 MHz) δ 196.36 (d, *J* = 14.0 Hz), 171.95 (d, *J* = 14.0 Hz). The reaction mixture was cooled down again to –78°C and, using a syringe, a solution of the second diol, 6,6'-(ethane-1,1-diyl)bis(2,4-di-*tert*-butylphenol) (1.666 g, 4.65 mmol) in THF (15 mL) was added in one portion, followed by $Et_3N$ (1.62 mL, 11.62 mmol). The reaction mixture was then allowed to warm up slowly for 1 h and then stirred at r.t. for a further 60 minutes. The reaction was filtered through a plug of $SiO_2$ (compacted with toluene) under an argon atmosphere and concentrated in *vacuo*. The resulting white solid was used without further purification containing compound **3c** as a small impurity as determined by $^{31}P\{^1H\}$ NMR (ca 1-3%) (4.5 g, 4.05 mmol, 87%). $^1H$ NMR ($C_6D_6$, 500 MHz) δ 10.29 (1H, d, *J* = 8.7 Hz, ArC*H*), 9.33 (1H, d, *J* = 7.4 Hz, ArC*H*), 9.12 (1H, d, *J* = 7.4 Hz, ArC*H*), 8.73 (1H, d, *J* = 7.4 Hz, ArC*H*), 8.42 (1H, d, *J* = 8.9 Hz, ArC*H*), 8.24 (1H, d, *J* = 8.9 Hz, ArC*H*), 8.14 (1H, d, *J* = 9.0 Hz, ArC*H*), 7.91 (1H, br s, ArC*H*), 7.14-6.73 (23H, m, ArCH, CH), 6.59 (1H, d, *J* = 2.8 Hz, ArC*H*), 6.57 (1H, d, *J* = 2.8 Hz, ArC*H*), 6.17 (1H, d, *J* = 6.5 Hz, C*H*), 3.33 (3H, s, $OCH_3$), 3.28 (3H, s, $OCH_3$), 2.34-2.20 (1H, m,

C$H_2$-CH$_2$), 2.01-1.85 (2H, m, C$H_2$-CH$_2$), 1.84-1.68 (1H, m, C$H_2$-CH$_2$), 1.40 (3H, s, CH$_3$), 1.36 (9H, s, C(CH$_3$)$_3$), 1.33 (9H, s, C(CH$_3$)$_3$), -0.59 (3H, s, CH$_3$). $^{13}$C NMR (C$_6$D$_6$, 126 MHz) δ 156.33-111.61 (20 x ArC), 130.28-113.37 (32 x ArCH), 83.01 (CH), 82.80 (CH), 55.11 (OCH$_3$), 55.14 (OCH$_3$), 35.55 (*C*(CH$_3$)$_3$), 35.52 (*C*(CH$_3$)$_3$), 31.25 (C(*C*H$_3$)$_3$), 31.17 (C(*C*H$_3$)$_3$), 28.41-27.84 (m, 2 x CH$_2$), 27.97 (CH$_3$), 24.08 (CH$_3$). $^{31}$P{$^1$H} NMR (C$_6$D$_6$, 202 MHz) δ 203.63 (d, $J$ = 17.7 Hz), 173.57 (d, $J$ = 17.7 Hz). HRMS (ES$^+$) [MH]$^+$ m/z: 1111.4448 found, $C_{71}H_{69}O_8P_2$ requires 1111.4462.

**3.2** 1,2-bis(4,8-di-*tert*-butyl-2,10-dimethoxydibenzo[*d,f*][1,3,2]dioxaphosphepin-6-yl)ethane, **3c.**

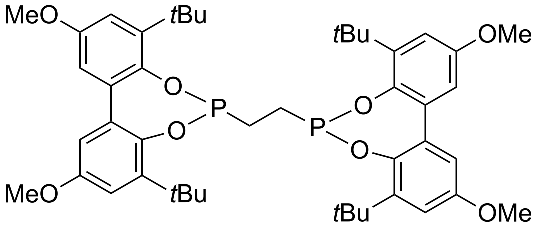


Compound **3c** was prepared according to a literature procedure and experimental data matched the reported data.[44] $^{31}$P{$^1$H} NMR (C$_6$D$_6$, 202 MHz) δ 202.66 (s).

**4. Synthesis of bis-phosphoramidite ligands 4a-4d.**

1,2-bis(4,8-di-*tert*-butyl-2,10-dimethoxydibenzo[*d,f*][1,3,2]dioxaphosphepin-6-yl)-1,2-dimethylhydrazine, **4a.**

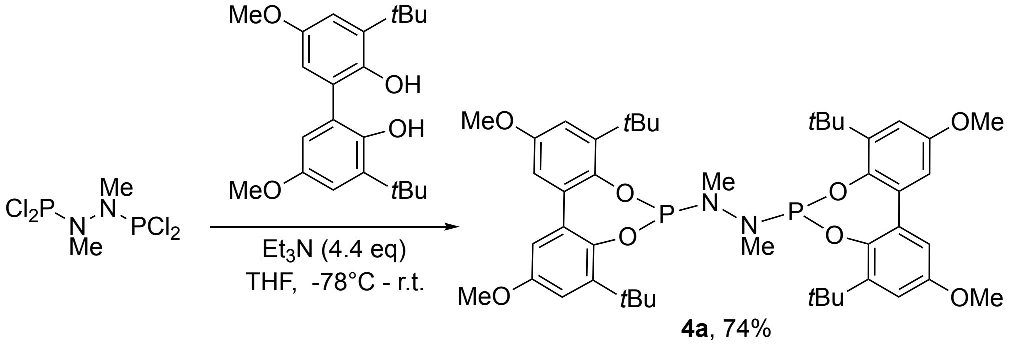


To a stirred solution of 1,2-bis(dichlorophosphaneyl)-1,2-dimethylhydrazine (0.166 g, 0.63 mmol) in THF (3 mL) at –78°C, was added a solution of 3,3'-di-*tert*-butyl-5,5'-dimethoxy-[1,1'-biphenyl]-2,2'-diol (0.454 g, 1.27 mmol) in THF (5 mL) slowly *via* syringe. This was followed by Et$_3$N (0.386 mL, 2.77 mmol) also added via syringe. The reaction was then allowed to warm to -45°C while stirring for 1 h, then the solution was taken out of the cold bath and stirred for a further 90 minutes at room temperature. The reaction was filtered under an argon atmosphere and concentrated in *vacuo*. The resulting solid was purified under air by flash chromatography on silica gel (5:1 petrol (40-60°C):EtOAc) affording the desired product **4a** (0.386 g, 0.463 mmol, 74%) as a white solid. $^1$H NMR (C$_6$D$_6$, 500 MHz) δ 7.20 (4H, br d, $J$ = 2.6 Hz, ArCH), 6.74 (4H, br d, $J$ = 2.6 Hz, ArCH), 3.36 (12H, s, 4 x OCH$_3$), 2.76 (6H, s, 2 x NCH$_3$), 1.62 (18H, s, 2 x C(CH$_3$)$_3$),

1.53 (18H, s, 2 x $C(CH_3)_3$). $^{13}C$ NMR ($C_6D_6$, 126 MHz) δ 155.75-155.69 (4 x ArC), 143.61 (2 x ArC), 143.31 (2 x ArC), 142.74 (2 x ArC), 142.16 (2 x ArC), 134.06 (2 x ArC), 133.44 (2 x ArC), 114.61 (4 x ArCH), 112.83 (4 x ArCH), 54.74 (4 x $OCH_3$), 35.64 (2 x $NCH_3$), 35.50 (2 x *C*$(CH_3)_3$), 35.18 (2 x *C*$(CH_3)_3$), 30.76 (4 x C(*C*$H_3)_3$). $^{31}P\{^1H\}$ NMR ($C_6D_6$, 202 MHz) δ 144.0 (s). HRMS (ES$^+$) $[MH]^+$ m/z: 833.4028 found, $C_{46}H_{63}O_8N_2P_2$ requires 833.4054.

1-(4,8-di-*tert*-butyl-2,10-dimethoxydibenzo[*d*,*f*][1,3,2]dioxaphosphepin-6-yl)-1,2-dimethyl-2-(2,4,8,10-tetra-*tert*-butyl-12-methyl-12*H*-dibenzo[*d*,*g*][1,3,2]dioxaphosphocin-6-yl)hydrazine, **4b**.

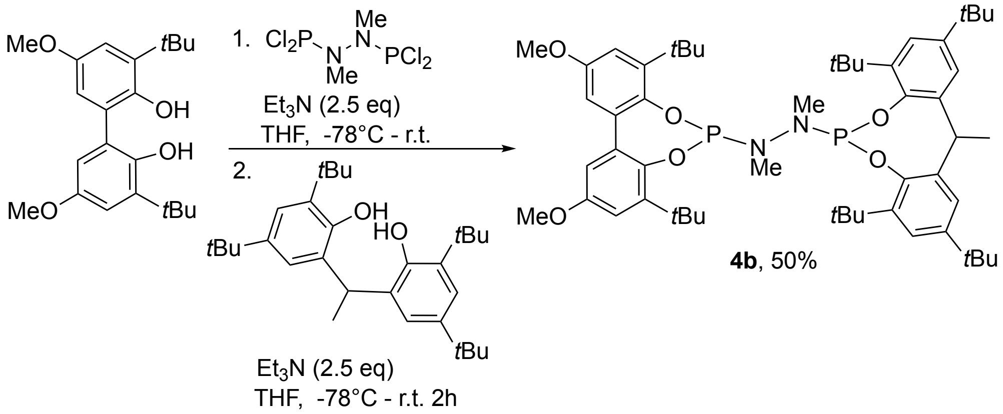


To a stirred solution of 3,3'-di-*tert*-butyl-5,5'-dimethoxy-[1,1'-biphenyl]-2,2'-diol (0.226 g, 0.630 mmol) in THF (4 mL) at –78°C, was added in one portion a solution of 1,2-bis(dichlorophosphaneyl)-1,2-dimethylhydrazine (0.165 g, 0.630 mmol) in THF (2 mL). This was followed by $Et_3N$ (0.220 mL, 1.575 mmol) also added via syringe. The reaction was then allowed to warm to -50°C while stirring for 1 h. The solution was taken out of the cold bath and stirred for a further 60 minutes at room temperature. The reaction mixture was cooled down again to –78°C and, using a syringe, a solution of the second diol, 6,6'-(ethane-1,1-diyl)bis(2,4-di-*tert*-butylphenol) (0.276 g, 0.630 mmol) in THF (3 mL) was added in one portion, followed by $Et_3N$ (0.220 mL, 1.575 mmol). The reaction mixture was then allowed to warm up slowly for 1 h and then stirred at r.t. for a further 60 minutes. The reaction was filtered under an argon atmosphere and concentrated in *vacuo*. The resulting solid was purified under air by flash chromatography on silica gel (15:1Hexane:$Et_2O$) affording the desired product **4b** (0.289 g, 0.316 mmol, 50%) as a white off solid (isomeric mixture in a 1:1.2 ratio as determined by $^{31}P\{^1H\}$ NMR). $^1H$ NMR ($C_6D_6$, 500 MHz) δ 7.44-7.35 (2 x 4H, br m, ArCH), 7.20 (2 x 2H, app br d, $J$ = 10.1 Hz, ArCH), 6.74 (2 x 2H, app br d, $J$ = 10.9 Hz, ArCH), 5.48 (1H, vbr s, CH), 4.17 (1H, vbr s, CH), 3.36 (2 x 9H, br s, 2 x $OCH_3$, $NCH_3$), 2.82 (2 x 3H, s, $NCH_3$), 2.10-1.26 (2 x 57H, br m, $CH_3$-CH, 6 x $C(CH_3)_3$). $^{13}C$ NMR ($C_6D_6$, 126 MHz) δ 155.84-128.20 (2 x 16 x ArC), 127.99 (2 x 2 x ArCH), 122.58 (2 x 2 x ArCH), 114.64 (2 x 2 x ArCH), 112.86 (2 x 2 x ArCH), 54.75 (2 x 2 x $OCH_3$), 35.38 (2 x 2 x *C*$(CH_3)_3$), 35.25 (2 x 2 x *C*$(CH_3)_3$), 34.25 (2 x 2 x *C*$(CH_3)_3$), 31.33-30.72 (2 x 2 $NCH_3$, 2 x 6 C(*C*$H_3)_3$). $^{31}P\{^1H\}$ NMR ($C_6D_6$, 202 MHz) δ 147.6 (br s), 144.4 (br s), 139.3 (br s), 131.5 (br s). HRMS (ES$^+$) $[MH]^+$ m/z: 913.5391 found, $C_{54}H_{79}O_6N_2P_2$ requires 913.5408.

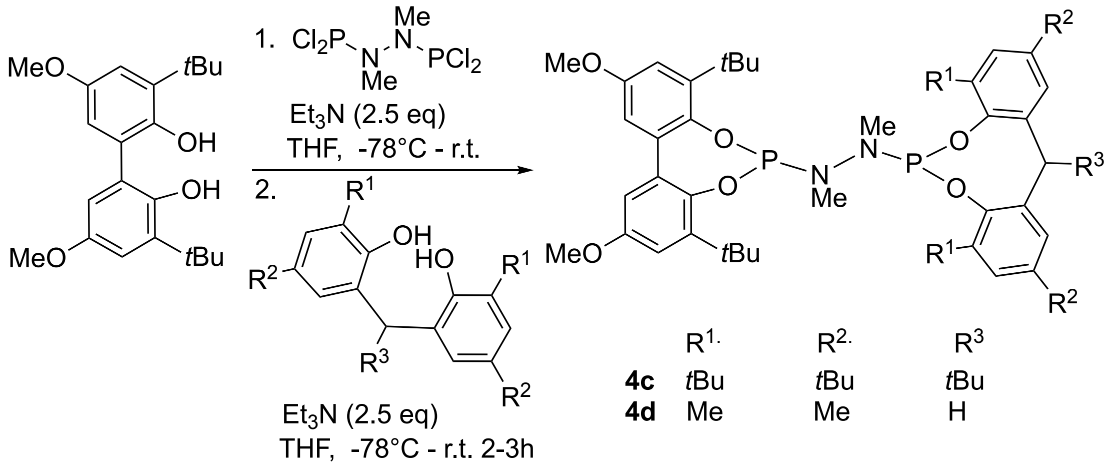


**Scheme S1.** Preparation of ligands **4c and 4d**

1-(4,8-di-*tert*-butyl-2,10-dimethoxydibenzo[*d,f*][1,3,2]dioxaphosphepin-6-yl)-1,2-dimethyl-2-(2,4,8,10,12-penta-*tert*-butyl-12*H*-dibenzo[*d,g*][1,3,2]dioxaphosphocin-6-yl)hydrazine, **4c**.

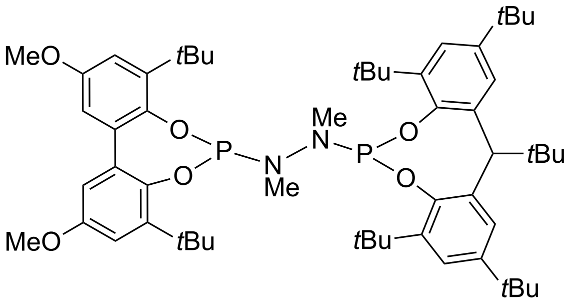


The compound was prepared using the same method as **4d** with 3,3'-di-*tert*-butyl-5,5'-dimethoxy-[1,1'-biphenyl]-2,2'-diol (0.339 g, 0.946 mmol), 1,2-bis(dichlorophosphaneyl)-1,2-dimethylhydrazine (0.236 g, 0.901 mmol) and 6,6'-(2,2-dimethylpropane-1,1-diyl)bis(2,4-di-*tert*-butylphenol) (0.433 g, 0.901 mmol). The resulting reaction crude (as an isomeric mixture in a 1:20 ratio) was purified by flash chromatography on silica gel (18:1 Hexane:$Et_2O$) affording the major isomer (0.349 g, 0.365 mmol, 40.5 %) as a white solid. A mixture of the major and minor isomer (as an isomeric mixture 1:11 ratio) was also isolated (0.158, 0.165 mmol, 18.4%). $^1$H NMR ($CDCl_3$, 400 MHz) δ 7.60 (2H, br s, ArCH), 7.14 (2H, br s, ArCH), 7.02 (1H, br s, ArCH), 6.96 (1H, br s, ArCH), 6.72 (2H, br s, ArCH), 4.96 (1H, s, CH), 3.83 (6H, s, 2 x $OCH_3$), 3.34 (3H, s, $NCH_3$), 2.74 (3H, s, $NCH_3$), 1.56 (9H, br s, $C(CH_3)_3$), 1.44 (9H, br s, $C(CH_3)_3$), 1.39 (9H, br s, $C(CH_3)_3$), 1.35 (9H, br s, $C(CH_3)_3$), 1.30 (18H, s, 2 x $C(CH_3)_3$), 1.10 (9H, s, $C(CH_3)_3$). $^{13}$C NMR ($CDCl_3$, 100 MHz) δ 155.44-132.45 (16 x ArC), 122.36 (ArCH), 122.14 (ArCH), 121.23 (2 x ArCH), 114.33 (2 x ArCH), 112.90 (ArCH), 112.50 (ArCH), 55.56 (2 x $OCH_3$), 45.07 (CH), 35.96 (2 x *C*($CH_3$)$_3$), 35.39 (2 x *C*($CH_3$)$_3$), 35.22 (*C*($CH_3$)$_3$), 35.07 ($NCH_3$), 34.50 (2 x *C*($CH_3$)$_3$), 32.79 ($NCH_3$), 31.59 (C(*C*$H_3$)$_3$), 31.17 (C(*C*$H_3$)$_3$), 31.12 (C(*C*$H_3$)$_3$), 30.88 (C(*C*$H_3$)$_3$), 30.83 (C(*C*$H_3$)$_3$), 30.72 (C(*C*$H_3$)$_3$), 30.70 (C(*C*$H_3$)$_3$). $^{31}$P{$^1$H}

NMR ($CDCl_3$, 161 MHz) δ 148.04 (s), 138.02 (s). HRMS (ES$^+$) [MH]$^+$ m/z: 955.5854 found, $C_{57}H_{85}O_6N_2P_2$ requires 955.5877.

1-(4,8-di-*tert*-butyl-2,10-dimethoxydibenzo[*d,f*][1,3,2]dioxaphosphepin-6-yl)-1,2-dimethyl-2-(2,4,8,10-tetramethyl-12*H*-dibenzo[*d,g*][1,3,2]dioxaphosphocin-6-yl)hydrazine, **4d**.

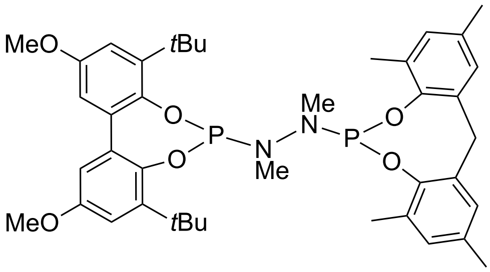


To a stirred solution of 3,3'-di-*tert*-butyl-5,5'-dimethoxy-[1,1'-biphenyl]-2,2'-diol (0.399 g, 1.110 mmol), in THF (4 mL) at –78°C, was added in one portion a solution of 1,2-bis(dichlorophosphaneyl)-1,2-dimethylhydrazine (0.292 g, 1.110 mmol) in THF (2 mL). This was followed by $Et_3N$ (0.387 mL, 2.775 mmol) also added via syringe. The reaction was then allowed to warm to -50°C while stirring for 1 h. The solution was taken out of the cold bath and stirred for a further 60 minutes at room temperature. The reaction mixture was cooled down again to –78°C and, using a syringe, a solution of the second diol, 6,6'-methylenebis(2,4-dimethylphenol) (0.378 g, 1.110 mmol) in THF (3 mL) was added in one portion, followed by $Et_3N$ (0.387 mL, 2.775 mmol). The reaction mixture was then allowed to warm up slowly for 1 h and then stirred at r.t. for a further 60 minutes. The reaction was filtered under an argon atmosphere and concentrated in *vacuo*. The resulting reaction crude was purified under air by flash chromatography on silica gel (3:1 Hexane:$Et_2O$) affording the desired product **4d** (0.436 g, 0.597 mmol, 53.7%) as a white off solid. $^1H$ NMR ($C_6D_6$, 400 MHz) δ 7.18 (2H, br s, ArCH), 6.90 (2H, br s, ArCH), 6.75 (2H, d, *J* = 3.1 Hz, ArCH), 6.68 (2H, br s, ArCH), 4.60 (1H, dd, *J* = 12.4, 3.1 Hz, *H*CH), 3.36 (6H, s, 2 x $OCH_3$), 3.34-3.30 (4H, m, $NCH_3$, *H*CH), 2.89 (3H, s, $NCH_3$), 2.42 (3H, br s, $CH_3$), 2.26 (3H, br s, $CH_3$), 2.04 (6H, s, 2 x $CH_3$), 1.56 (9H, s, $C(CH_3)_3$), 1.47 (9H, s, $C(CH_3)_3$). $^{13}C$ NMR ($C_6D_6$, 100 MHz) δ 155.72-133.37 (16 x ArC), 129.85 (2 x ArCH), 128.00 (2 x ArCH), 114.57 (2 x ArCH), 112.88 (2 x ArCH), 54.75 (2 x $OCH_3$), 35.49 (d, *J* = 5.4 Hz, $NCH_3$), 35.30 (2 x *C*$(CH_3)_3$), 32.97 ($NCH_3$), 30.70 (2 x C(*C*$H_3)_3$), 20.38 (2 x $CH_3$), 17.63 (br s, $CH_3$) 17.20 (br s, $CH_3$). $^{31}P\{^1H\}$ NMR ($C_6D_6$, 162 MHz) δ 145.9 (d, *J* = 20.3 Hz), 135.7 (d, *J* = 20.3 Hz). HRMS (ES$^+$) [MH]$^+$ m/z: 731.370 found, $C_{41}H_{53}O_6N_2P_2$ requires 731.3373.

## 4.1 Variable temperature NMR spectroscopy measurement of ligand 4b.

The spectrum of ligand **4b** in solution ($C_6D_6$) showed a marked broadness of the $^{31}P\{^1H\}$ and some of the $^1H$ NMR resonances, especially the bridging CH. This fact itself suggests that ligand **4b** exists in solution as a mixture of isomers that exchange under equilibrium conditions. In contrast ligand **4c** (with a *t*Bu group in the carbon bridge instead of a methyl group as in the case of **4b**) was prepared as a mixture of isomers that gave much sharper resonances and, in fact, the major isomer could be separated by chromatography on $SiO_2$. Heating the major isomer of **4c** at 110 °C in toluene for two hours and running a $^{31}P\{^1H\}$ NMR afterwards did not show the formation of any amount of the minor isomer, confirming that for that ligand, there is no exchange between isomers in solution.

Variable temperature NMR spectroscopic measurements were carried out. Low temperatures being used to see if further peaks are resolved and high temperatures to confirm the exchange process. At room temperature there are 4 broad signals in the $^{31}P\{^1H\}$ NMR. These correspond to the two different phosphorous environments (the 8-membered phosphacycle comes around 10 ppm upfield from the 7 membered one for ligands 4b-4d). There are then two isomers detected, and no P-P coupling observed. Heating a solution of **4b** in d8-toluene up to 80°C (**Figure S3.a)**) afforded two signals in the $^{31}P\{^1H\}$ NMR spectrum, with the two signals (145.7 and 135.5 ppm) arising from isomerism achieving coalescence, confirming the exchange process. The $^1H$ NMR of the same mixture also showed coalescence of the C*H*-Methyl resonances for both isomers into a single signal at 4.99 ppm, somewhere in the middle of the signals observed at 25 °C for both isomers (**Figure S5**). Given that ligand **4c** shows the same behaviour in the crude mixture but the isomers are separable, and **4d**, with a $CH_2$ bridge does not show this isomerism, the isomers are assigned as being of type A and B, differing in the methyl group and P lone pair being *syn* or *anti* to each other.

Cooling down the sample to –80°C allowed us to observe a relative sharpening of the $^{31}P\{^1H\}$ resonances. In addition, the furthest upfield peak resolves into a further signal (there is a hint that the peak at 142 ppm may be beginning to split also). A total of 4 isomers were observed (147.4 (br s, 3xP), 142.0 (br s, P), 137.8 (br s, 2 x P), 130.3 (br s, P), 129.6 (br s, P). The gap between the peaks is too large to be consistent with P-P coupling, so is ascribed to the appearance of the pseudo E/Z isomers that can arise from restricted rotation about the N-N bond (**Figure S3.c)**).

**Figure S2.** Possible structures of isomers **4b** observed at 25°C, 80°C and –80°C.

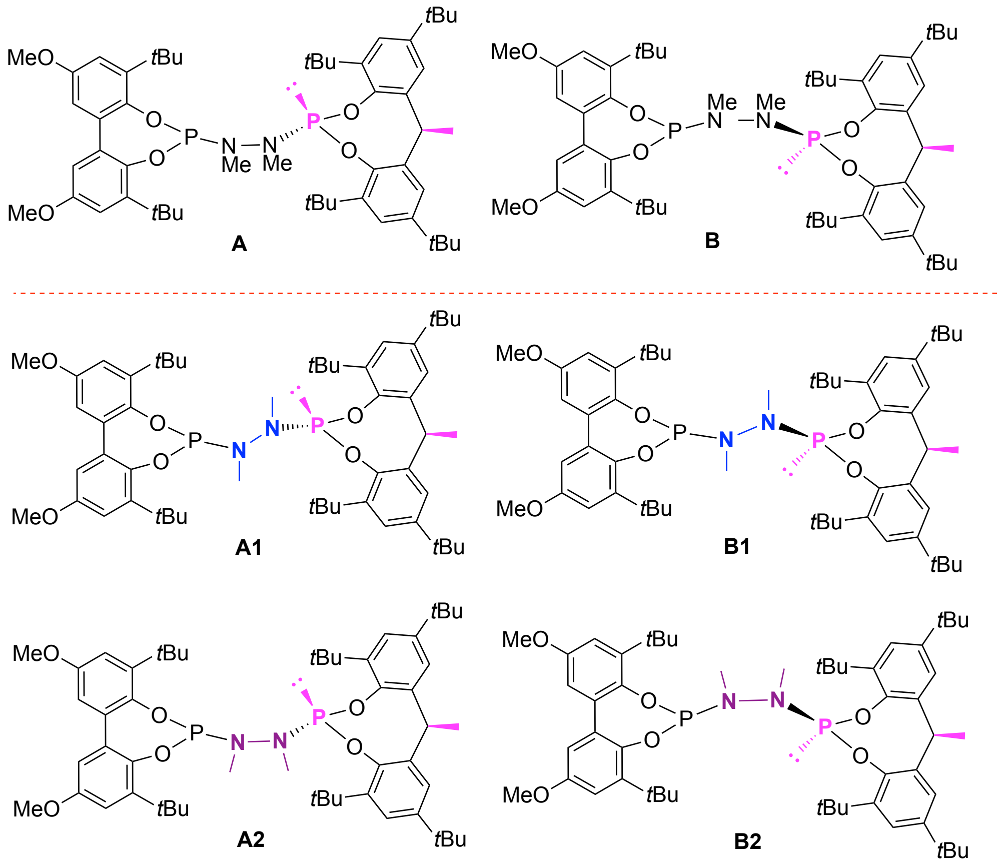


**Figure S3.** $^{31}$**P{**$^{1}$**H}-NMR** (d8-toluene) spectra of **4b** at 80 °C (a), 25°C (b), and –80 °C (c)

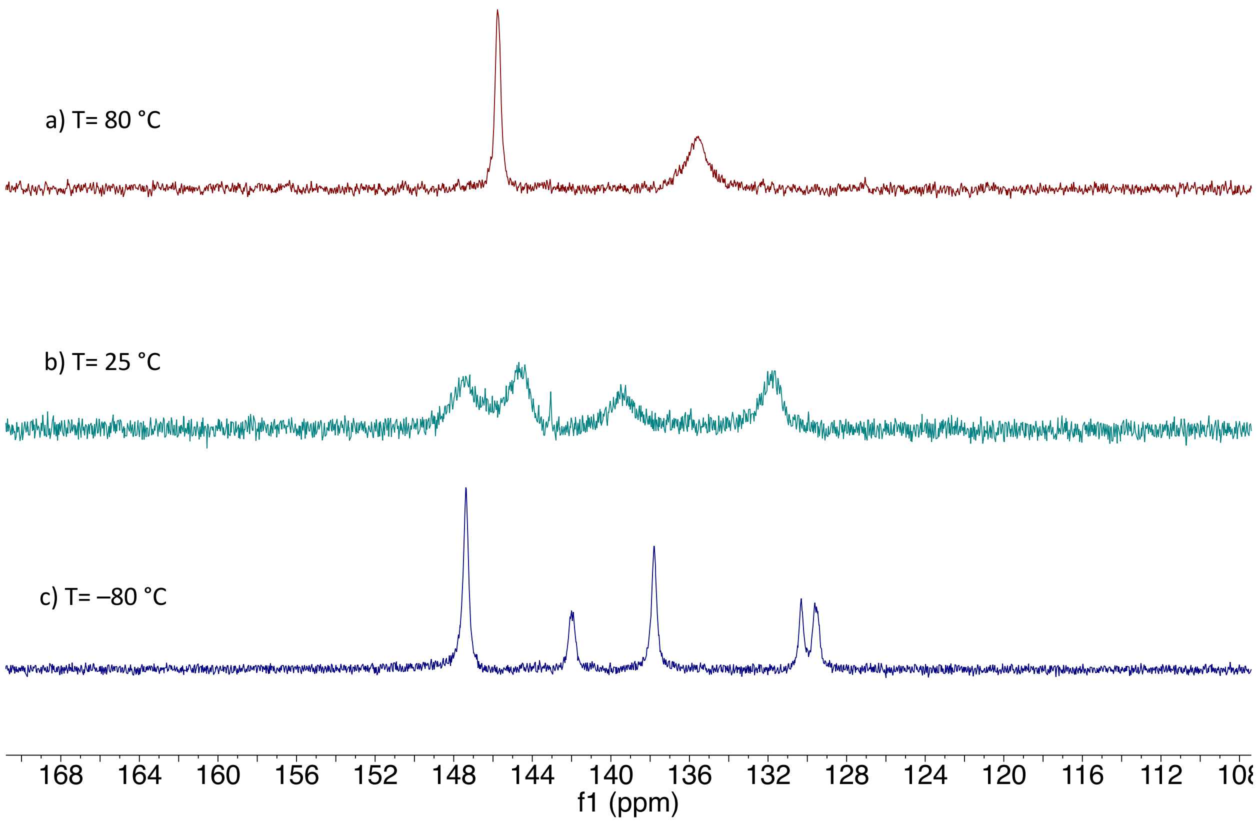

**Figure S4.** [1]**H-NMR** (d8-toluene) spectra of **4b** at 80 °C (a), rt (b), and –80 °C (c)

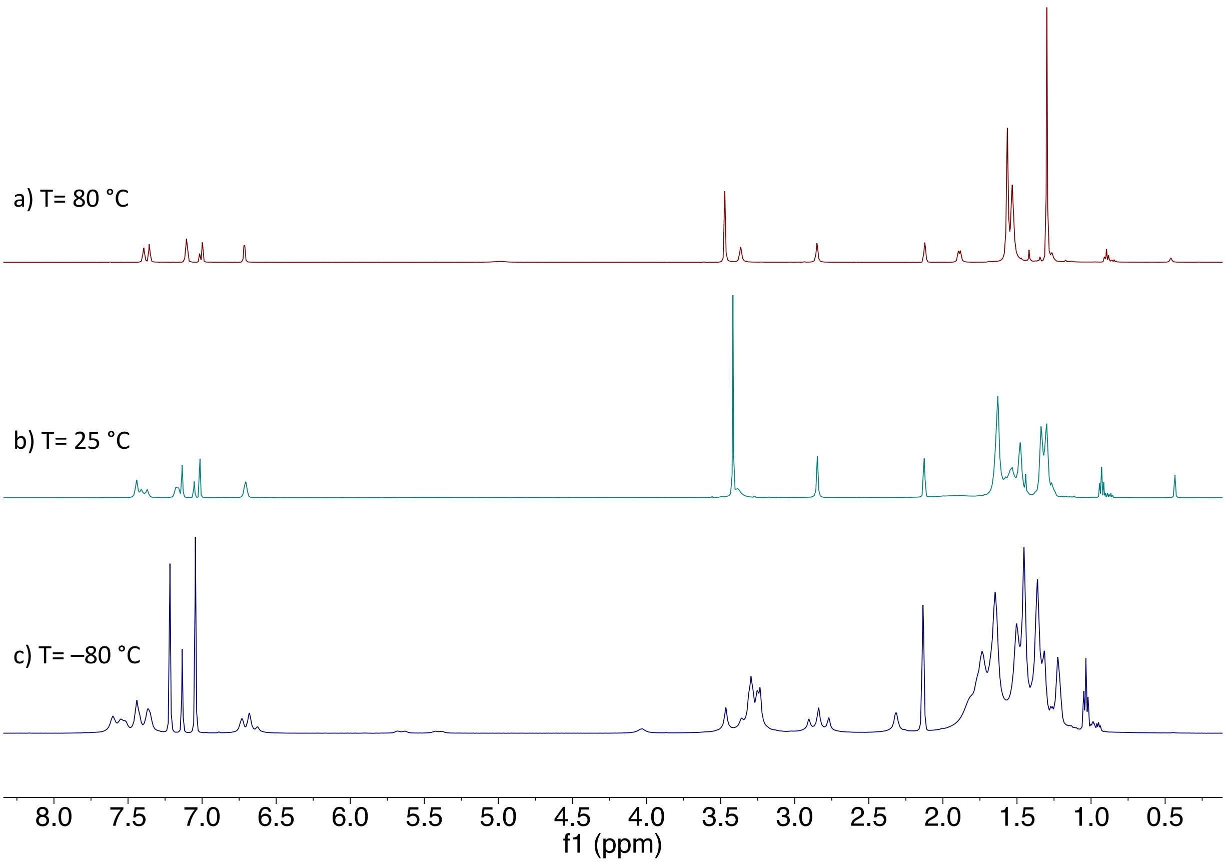


**Figure S5.** [1]**H-NMR** (d8-toluene) spectra of **4b** at 80 °C (a), 25°C (b), and –80 °C (c). Expansion showing the CH bridge region.

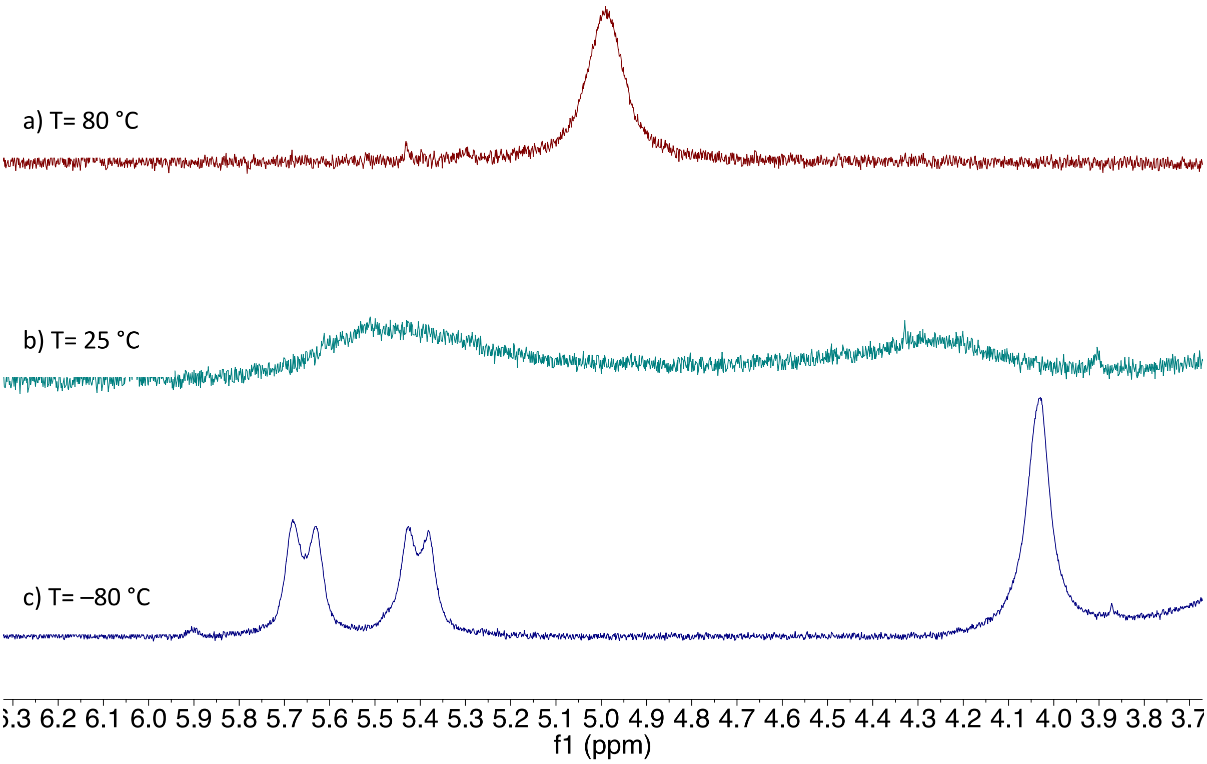

**Figure S6.** $^{1}$**H-NMR** (d8-toluene) spectra of **4b** at 80 °C (a), 25°C (b), and –80 °C (c). Expansion showing the alkyl groups region.

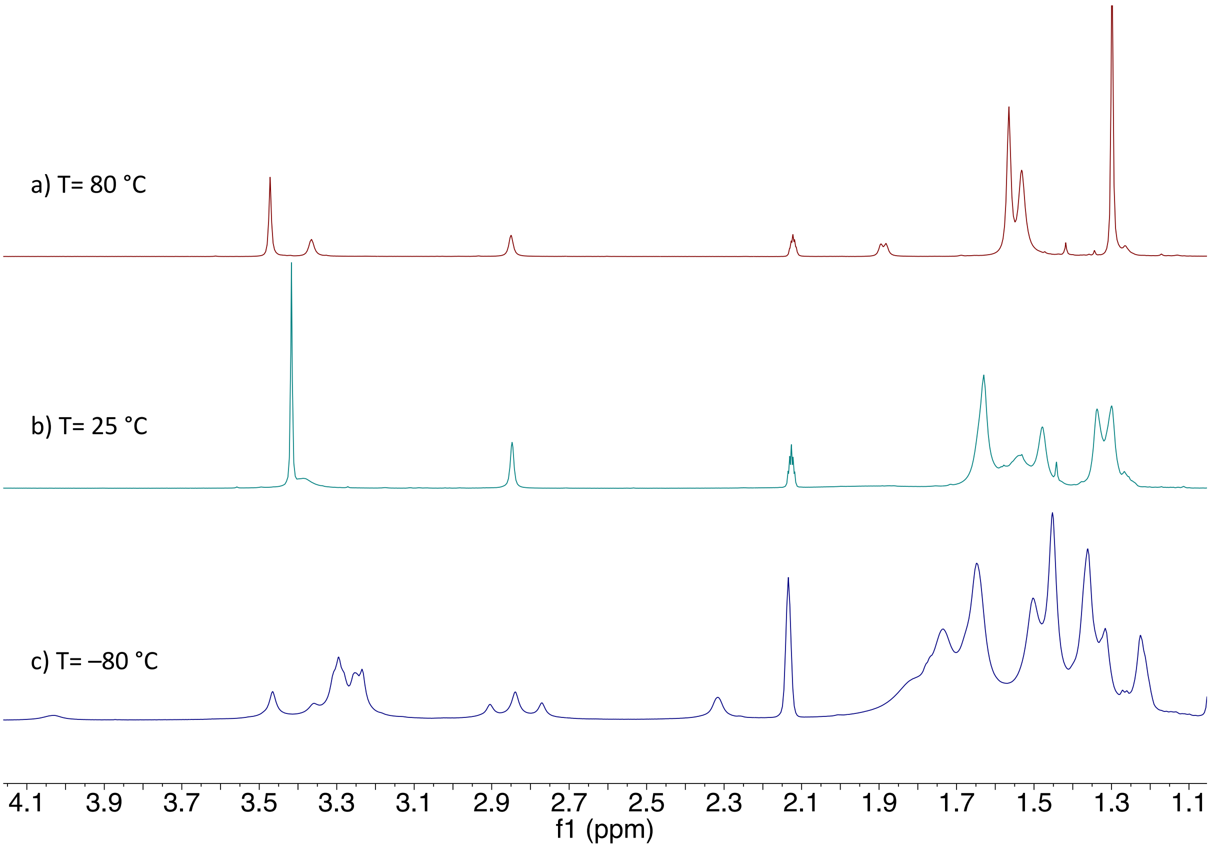

## 5. Bis-phosphoramidite ligands, relative stability to moisture compared to phosphonites

**Figure S7.** $^{31}$**P{$^1$H}-NMR** (toluene, $C_6D_6$ capillary) spectra of **3c** after 18h.

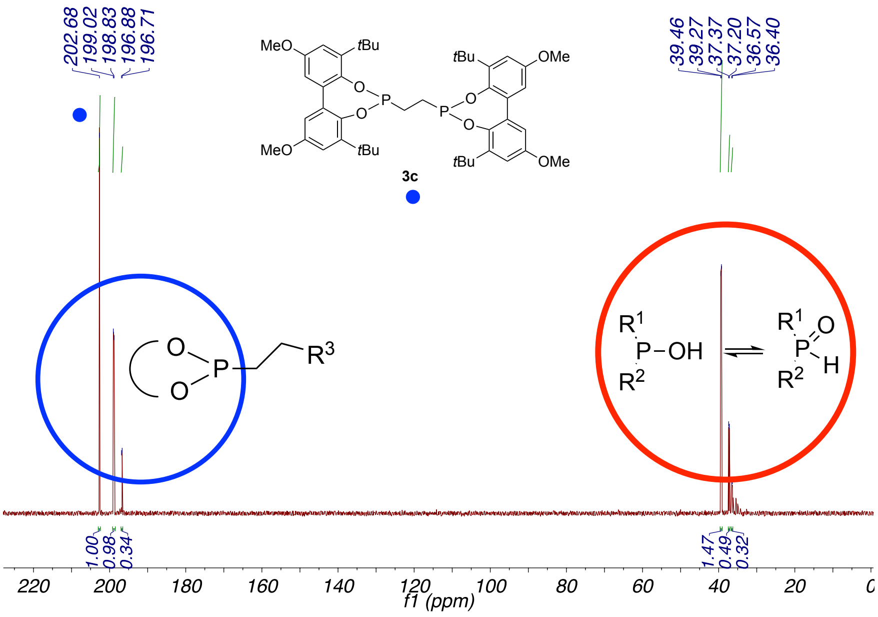


Ligand **3c** was dissolved in standard "out of the bottle" toluene under air and left overnight at r.t. A $^{31}$P NMR was then recorded showing a mixture of products including the original ligand **3c** and decomposition products resulting from the hydrolysis of ligand **3c**.

**Figure S8.** $^{31}$**P{$^1$H}-NMR** ($CDCl_3$ + 1drop of water) spectra of **4a** after one week.

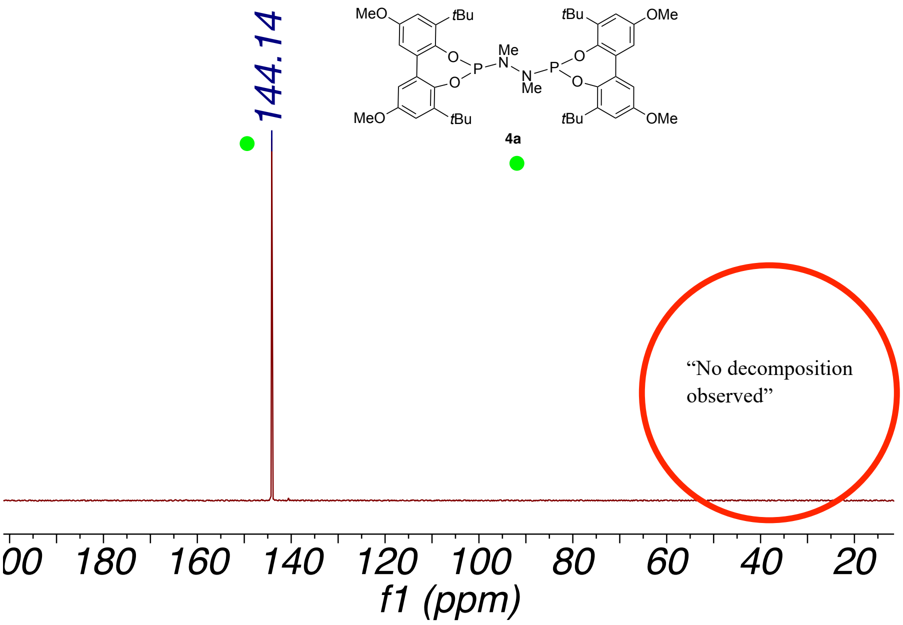


Ligand **4a** was dissolved in standard "out of the bottle" $CDCl_3$ under air, a drop of water was added to the solution and left one week at r.t. A $^{31}$P NMR was then recorded not showing any decomposition products resulting from the hydrolysis of ligand **4a**.

## 6. Preparation of Pt complexes.

### 6.1 Preparation of [Pt(4a)$Cl_2$].

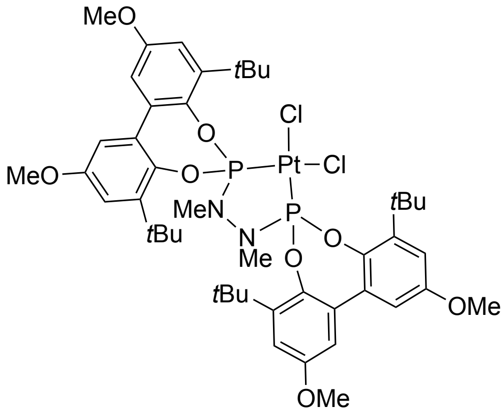


A Schlenk flask was charged with [$PtCl_2$(COD)] (24 mg, 0.064mmol) and **4a** (58 mg, 0.069 mmol). The solids were purged with three vacuum/argon cycles and left under an argon atmosphere. $CH_2Cl_2$ (2.0 mL) was added, and the solution was stirred at room temperature for 22 h. The solvent was then removed under reduced pressure and then 2 ml of hexane was added, and the mixture vigorously stirred for 2 h. The resulting solid was filtered off and washed with 2 ml of $Et_2O$ to afford the required complex as a white solid (62 mg, 0.056 mmol, 88%). X-Ray quality crystals were grown by slow diffusion of hexane over a $CDCl_3$ solution of the complex. (**Figure S9**). $^1$H NMR ($CDCl_3$, 500 MHz) δ 7.00 (4H, d, $J$ = 2.9 Hz, ArCH), 6.70 (4H, d, $J$ = 2.9 Hz, ArCH), 3.82 (12H, s, 4 x $OCH_3$), 2.86 (6H, s, 2 x $NCH_3$), 1.54 (36H, br s, 2 x $C(CH_3)_3$). $^{13}$C NMR (126 MHz, $CDCl_3$) δ 156.68 (br s, 8 x ArC), 142.13 (8 x ArC), 115.34 (br s, 4 x ArCH), 113.65 (4 x ArCH), 55.77 (4 x $OCH_3$), 35.84 (br s, 4 x $C(CH_3)_3$), 33.59 (2 x $NCH_3$), 32.00 (br s, 4 x $C(CH_3)_3$). $^{31}$P{$^1$H} NMR ($CDCl_3$, 202 MHz) δ 96.86 (s with Pt satellites, $^1J_{P\text{-}Pt}$ 5582 Hz). HRMS (ES$^+$) $C_{46}H_{63}O_8ClN_2P_2Pt$ [MH-Cl]$^+$ m/z: 1063.3305 found, 1063.3390 required.

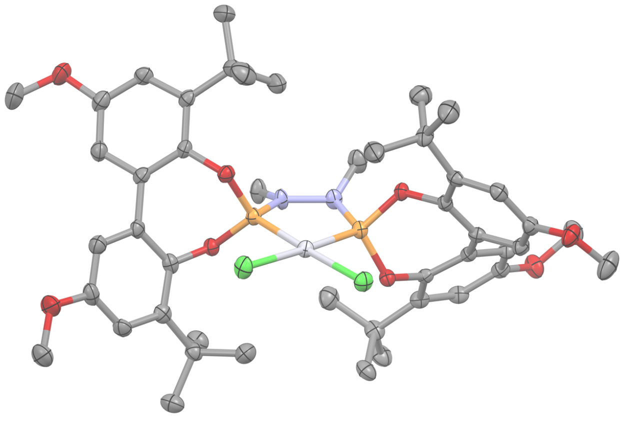

**Figure S9.** View of an independent molecule in the structure of complex **[Pt(4a)$Cl_2$]**. Hydrogen atoms omitted for clarity.

**6.2 Preparation of [Pt(4b)$Cl_2$].**

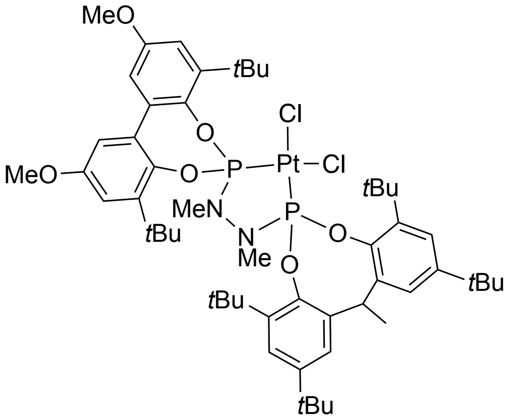


A Schlenk flask was charged with [$PtCl_2$(COD)] (47.5 mg, 0.127 mmol) and **4b** (127 mg, 0.139 mmol). The solids were purged with three vacuum/argon cycles and left under an argon atmosphere. $CH_2Cl_2$ (2.0 mL) was added, and the solution was stirred at room temperature for 22 h. The solvent was then removed under reduced pressure and then 2 ml of hexane was added, and the mixture vigorously stirred for 2 h. The resulting solid was filtered off and washed with 4 ml of $Et_2O$ to afford the required complex as a white solid (136 mg, 0.115 mmol, 91%). X-Ray quality crystals were grown by slow diffusion of hexane over a DCM solution of the complex. (**Figure S10**). $^{1}$H NMR ($CDCl_3$, 400 MHz) δ 7.27-7.21 (4H, br m, ArCH), 7.00 (2H, d, $J$ = 3.0 Hz, ArCH), 6.70 (2H, d, $J$ = 3.0 Hz, ArCH), 5.46 (1H, q, $J$ = 6.7 Hz, CH), 3.82 (6H, s, 2 x $OCH_3$), 3.00 (3H, br s, $NCH_3$), 2.99 (3H, br s, $NCH_3$), 1.62 (d, $J$ = 6.8 Hz, 3H, CH-C$H_3$), 1.55 (18H, br s, 2 x C($CH_3$)$_3$), 1.49 (18H, s, 2 x C($CH_3$)$_3$), 1.30 (18H, s, 2 x C($CH_3$)$_3$). $^{13}$C NMR (101 MHz, $CDCl_3$) δ 156.55-131.49 (16 x ArC), 123.39 (2 x ArCH), 122.45 (2 x ArCH), 115.21 (2 x ArCH), 113.80 (2 x ArCH), 55.78 (2 x $OCH_3$), 35.74 (2 x $C$($CH_3$)$_3$), 35.51 (2 x $C$($CH_3$)$_3$), 35.22 ($NCH_3$), 34.85 (2 x $C$($CH_3$)$_3$), 33.90 (CH), 32.78 (d, $J$= 4 Hz, $NCH_3$), 32.45 (2 x C($CH_3$)$_3$), 31.86 (br s, 2 x C($CH_3$)$_3$), 31.60 (2 x C($CH_3$)$_3$), 20.60 (CH-$CH_3$)$_3$). $^{31}$P{$^{1}$H} NMR ($CDCl_3$, 162 MHz) δ 94.05 (d, $^{2}J_{P-P}$= 19.5 Hz with Pt satellites, $^{1}J_{P-Pt}$ 5581 Hz), 82.68 (d, $J_{P-P}$= 19.5 Hz with Pt satellites, $^{1}J_{P-Pt}$ 5621 Hz). HRMS (ES$^+$) $C_{54}H_{78}O_6ClN_2P_2Pt$ [MH-Cl]$^+$ m/z: 1143.4675 found, 1143.4744 required.

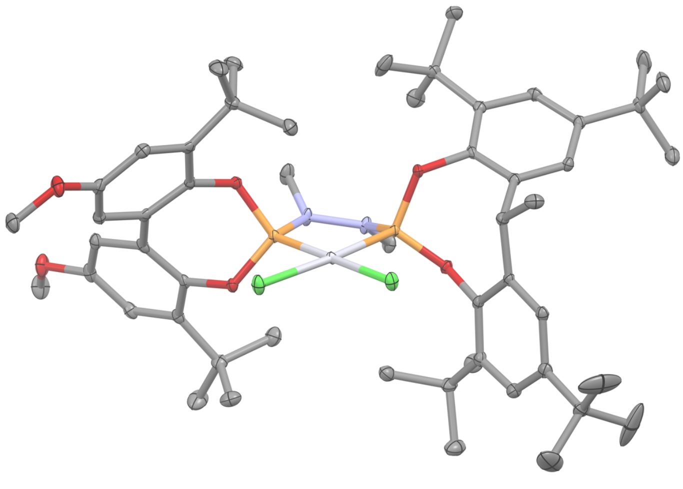

**Figure S10.** View of an independent molecule in the structure of complex **[Pt(4b)$Cl_2$]**. Hydrogen atoms omitted for clarity.

## 6.3 NMR monitoring of the formation of [Pt(4b)$Cl_2$].

An NMR tube was charged with [$PtCl_2$(COD)] (4.5 mg, 0.012 mmol), **4b** (1eq. [a], 2 eq. [b]) and $CDCl_3$ (0.6 mL). The resulting solution was then monitored by NMR.

**[a] 1 eq. of 4b.** $^{31}P\{^{1}H\}$ NMR spectra of [Pt(**4b**)$Cl_2$]. The presence of free ligand can be observed after 5 minutes but only one isomer of the complex is observed in solution. After 75 minutes then same isomer of the complex is observed.

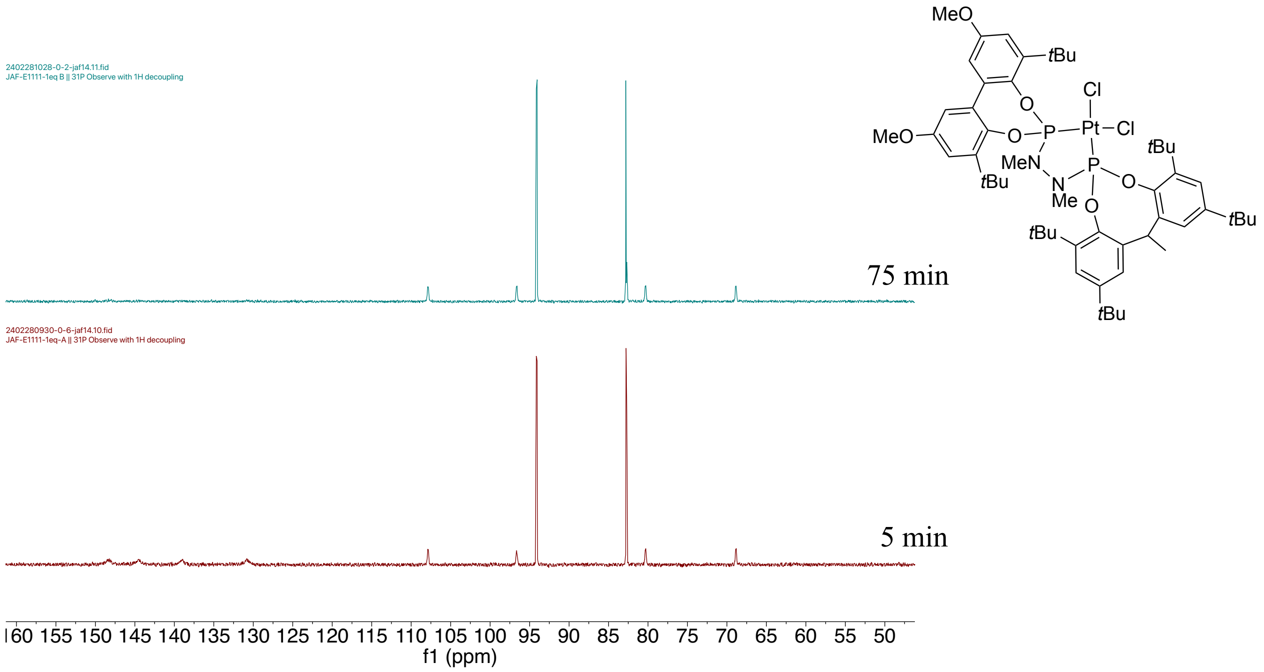


**[b] 2 eq. of 4b.** $^{31}P\{^{1}H\}$ NMR spectra of [Pt(**4b**)$Cl_2$]. The presence of an excess of ligand **4b** does not lead to a preferential coordination of one of the isomers relative to the other one. Only one of the isomers of the complex can be observed after 5 or 75 minutes.

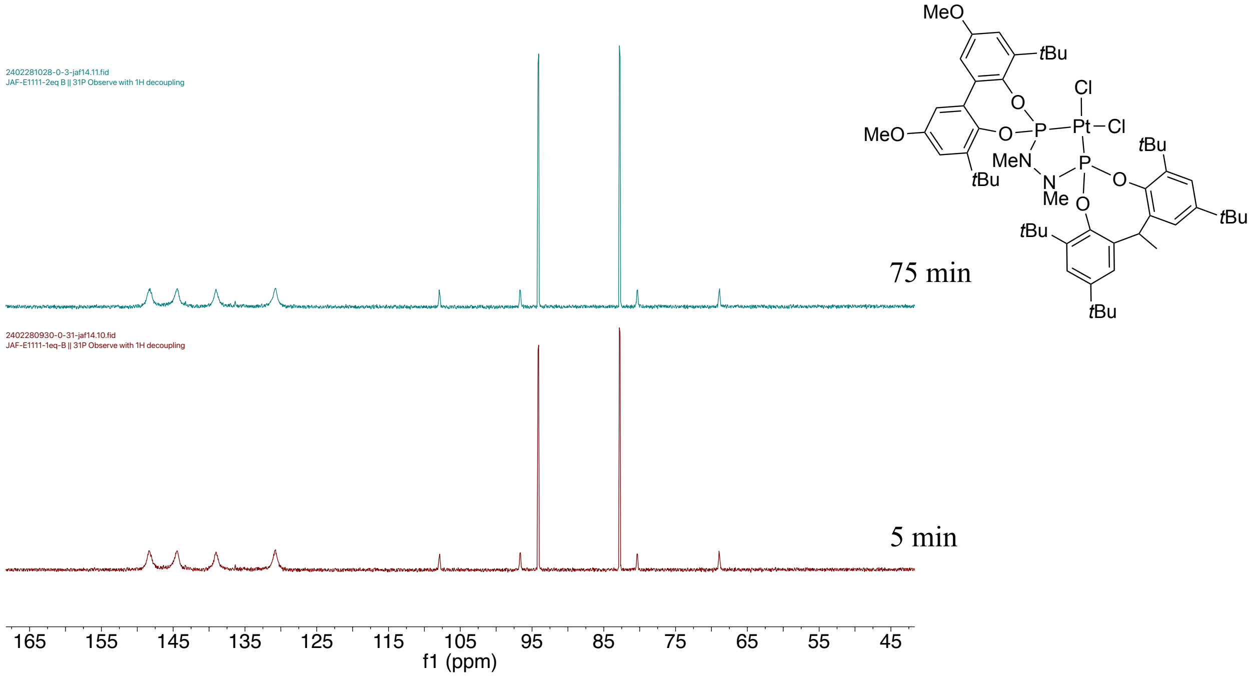

## 7. Experimental data for products from catalysis:

### 7.1. Hydroformylation of allylbenzene, 5a.

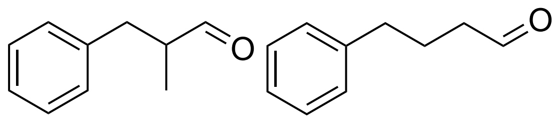

Hydroformylation was performed following the general method by reaction of allylbenzene **5a** (1 mmol, 132 µL). The products were isolated as the corresponding alcohols obtained by reduction with $NaBH_4$ and isolated by column chromatography on silica gel using hexane/EtOAc 4:1 as eluent to give the branched and linear alcohols as a colourless oil (130 mg, 87% combined yield). The NMR data matches previously reported data for the title compounds. **2-Methyl-3-phenylpropanal**.[45] $^1$H NMR (500 MHz, $C_6D_6$): δ= 9.31 (1H, d, *J* = 1.4 Hz, C*H*O), 7.15-7.01 (3H, m), 6.88 (2H, d, *J* = 7.3 Hz, ArCH), 2.73 (1H, dd, *J* = 13.6, 5.8 Hz, C*H*$_2$), 2.21-2.07 (2H, m, C*H*, C*H*$_2$), 0.71 (3H, d, *J* = 7.0 Hz, $CH_3$). $^{13}$C NMR (126 MHz, $C_6D_6$): 202.72 (CHO), 139.38 (ArC), 129.29 (2 x ArCH), 128.69 (2 x ArCH), 126.54 (ArCH), 47.94 (CH), 36.64 ($CH_2$), 12.98 ($CH_3$). **4-phenylbutanal**.[46] $^1$H NMR (500 MHz, $C_6D_6$): δ= 9.23 (1H, t, *J* = 1.4 Hz, C*H*O), 7.15-7.01 (3H, m), 6.94 (2H, d, *J* = 7.2 Hz, ArCH), 2.27 (2H, t, *J* = 7.6 Hz, C*H*$_2$), 1.76-1.73 (2H, m, C*H*$_2$), 1.60-1.54 (2H, m, C*H*$_2$), $^{13}$C NMR (126 MHz, $C_6D_6$): 200.39 (CHO), 141.69 (ArC), 128.74 (2 x ArCH), 128.70 (2 x ArCH), 126.31 (ArCH), 42.99 ($CH_2$), 35.16 ($CH_2$), 23.82 ($CH_2$).

### 7.2. Hydroformylation of allylcyanide, 5b.

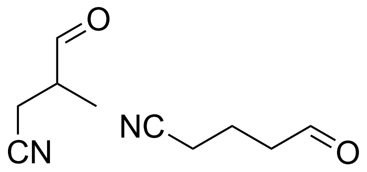

Hydroformylation was performed following the general method by reaction of allylcyanide **5b** (1 mmol, 81 µL). The products were isolated by column chromatography on silica gel using hexane/$Et_2O$ 1:3 as eluent to give the branched and linear aldehydes as a colourless oil (71 mg, 73% combined yield). **3-Methyl-4-oxobutanenitrile**.[37] $^1$H NMR (400 MHz, $CDCl_3$): δ= 8.82 (1H, br d, C*H*O), 1.74-1.69 (1H, m, C*H*$_2$), 1.62-1.50 (2H, m, C*H*, C*H*$_2$), 0.55 (3H, d, *J* = 7.4 Hz, $CH_3$). $^{13}$C NMR (126 MHz, $C_6D_6$) δ 199.17 (CHO), 128.35 (CN), 42.26 (CH), 17.27 ($CH_2$), 12.53 ($CH_3$). **5-Oxopentanenitrile**.[37] $^1$H NMR (400 MHz, $C_6D_6$): 9.0 (1H, br t, C*H*O), 1.62-1.54 (2H, m, C*H*$_2$), 1.40 (2H, t, *J*= 7.4 Hz, C*H*$_2$), 1.14-1.08 (3H, m, $CH_2$). $^{13}$C NMR (126 MHz, $C_6D_6$) δ 198.96 (CHO), 117.68 (CN), 41.60 ($CH_2$), 17.92 ($CH_2$), 15.86 ($CH_2$).

### 7.3. Hydroformylation of 1-octene, 5c.

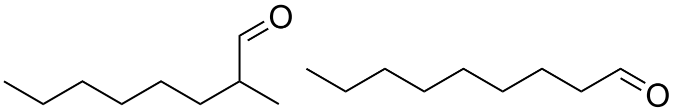

Hydroformylation was performed following the general method by reaction of 1-octene (1 mmol, 157 µL). The products were isolated by column chromatography on silica gel using hexane/EtOAc 10:1 as eluent to give the branched and linear aldehydes as a colourless oil (101 mg, 71% combined yield). **2-Methyloctanal**.[47] $^1$H NMR (400 MHz, $C_6D_6$): δ= 9.31 (1H, d, *J* = 1.8 Hz, C*H*O), 1.91-1.83 (1H, m, CH), 1.43-0.96 (10H, 5x $CH_2$), 0.89 (3H, t, *J* = 7.1 Hz), 0.79 (3H, d, *J* = 7.0 Hz). **Nonanal**.[47] $^1$H NMR (400 MHz, $C_6D_6$): δ= 9.51 (1H, t, *J* = 1.7 Hz, C*H*O), 1.83 (2H, td, *J* = 7.3, 1.8 Hz, C*H*$_2$CHO), 1.43-0.96 (12H, 6x $CH_2$), 0.88 (3H, t, *J* = 7.1 Hz),

### 7.4. Hydroformylation of 1-((but-3-en-1-yloxy)methyl)-4-methoxybenzene, 5d.

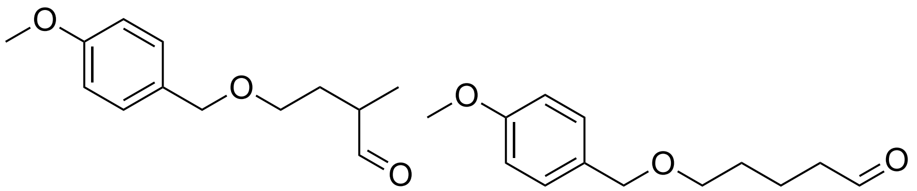

Hydroformylation was performed following the general method by reaction of 1-((but-3-en-1-yloxy)methyl)-4-methoxybenzene (1 mmol, 192 mg). The products were isolated by column chromatography on silica gel using hexane/EtOAc 4:1 as eluent to give the branched and linear aldehydes as a pale-yellow oil (188 mg, 85% combined yield). **4-((4-methoxybenzyl)oxy)-2-methylbutanal.**[48] $^1$H NMR (400 MHz, $C_6D_6$): δ= 9.38 (1H, d, *J* = 1.6 Hz, C*H*O), 7.18 (2H, d, *J* = 8.7 Hz, ArCH), 6.81 (2H, d, *J* = 8.7 Hz, ArCH), 4.21 (2H, s, Ar$CH_2$O), 3.31 (3H, s, $CH_3$O), 3.24-3.14 (2H, m, $CH_2$O), 2.18-2.09 (1H, m, CH), 1.77-1.69 (1H, m, $CH_2$), 1.77-1.69 (1H, m, $CH_2$), 1.38-1.30 (1H, m, $CH_2$), 0.78 (3H, d, *J* = 7.1 Hz, $CH_3$). $^{13}$C NMR (101 MHz, $C_6D_6$) δ 203.07 (CHO), 159.78 (ArC), 130.99 (ArC), 129.45 (2 x ArCH), 114.10 (2 x ArCH), 72.82 (Ar*C*H$_2$O), 67.17 ($CH_2$O), 54.80 ($CH_3$O), 43.87 (CH), 31.07 ($CH_2$), 13.19 ($CH_3$). **5-((4-methoxybenzyl)oxy)pentanal.**[49] $^1$H NMR (400 MHz, $C_6D_6$): δ= 9.28 (1H, t, *J* = 1.6 Hz, C*H*O), 7.21 (2H, d, *J* = 8.7 Hz, ArCH), 6.81 (2H, d, *J* = 8.7 Hz, ArCH), 4.28 (2H, s, Ar$CH_2$O), 3.31 (3H, s, $CH_3$O), 3.19 (2H, t, *J* = 6.0 Hz, $CH_2$O), 1.81 (2H, t, *J* = 1.6 Hz, C*H*$_2$CHO), 1.49-1.33 (4H, m, 2 x $CH_2$). $^{13}$C NMR (101 MHz, $C_6D_6$) δ 200.69 (CHO), 159.75 (ArC), 131.32 (ArC), 129.39 (2 x ArCH), 114.10 (2 x ArCH), 72.74 (Ar*C*H$_2$O), 69.57 ($CH_2$O), 54.81 ($CH_3$O), 43.52 (*C*H$_2$CHO), 29.47 ($CH_2$), 19.20 ($CH_2$).

**7.5. Hydroformylation of 2-allylisoindoline-1,3-dione (allylphthalimide), 5e.**

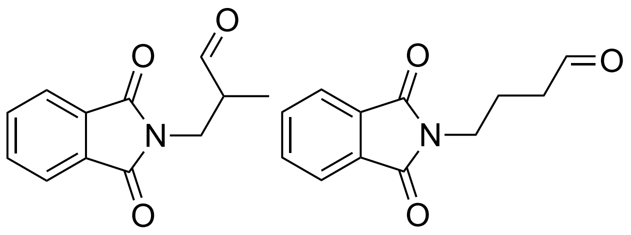

Hydroformylation was performed following the general method by reaction of allylphthalimide (1 mmol, 187.2 mg). The products were isolated by column chromatography on silica gel using hexane/EtOAc 3:1 as eluent to give the branched aldehyde and linear aldehydes as a pale-yellow oil (185 mg, 85% combined yield). **3-(1,3-Dioxoisoindolin-2-yl)-2-methylpropanal.**[2] $^1$H NMR (500 MHz, $C_6D_6$): δ= 9.36 (1H, br d, C*H*O), 7.41-7.39 (2H, m, ArCH), 6.89-6.87 (2H, m, ArCH), 3.67 (1H, dd, *J* = 14.1, 7.0 Hz, C*H*H), 3.44 (1H, dd, *J* = 14.1, 6.4 Hz, C*H*H), 2.43-2.36 (1H, m, CH), 0.71 (3H, d, *J* = 7.2 Hz, $CH_3$). $^{13}$C NMR (101 MHz, $C_6D_6$) δ 201.14 (CHO), 167.93 (ArC), 133.67 (2 x ArCH), 132.29 (ArC), 123.11 (2 x ArCH), 45.98 (*C*H), 38.12 (*C*$H_2$N), 11.28 ($CH_3$). **4-(1,3-dioxoisoindolin-2-yl)butanal.**[50] $^1$H NMR (500 MHz, $C_6D_6$): δ= 9.36 (1H, br t, C*H*O), 7.46-7.45 (2H, m, ArCH), 6.92-6.90 (2H, m, ArCH), 3.36 (2H, t, *J* = 6.7, NC$H_2$), 1.75-1.72 (2H, m, C$H_2$), 1.67-1.61 (2H, m, C$H_2$). $^{13}$C NMR (101 MHz, $C_6D_6$) δ 199.43 (CHO), 167.95 (ArC), 133.55 (2 x ArCH), 132.58 (ArC), 122.97 (2 x ArCH), 40.92 (*C*$H_2$N), 37.13 (*C*$H_2$CHO), 21.33 ($CH_2$).

**8. Procedure for the rhodium-catalysed hydroformylation of propene. Gas-uptake experiment using 4a as ligand.**

Hydroformylation of propene was performed in a Parr 4590 Micro Reactor fitted with a gas entrainment stirrer; comprising of holes which gives better gas dispersion throughout the reaction mixture. The vessel had a volume capacity of 0.1 L, an overhead stirrer with gas entrainment head (set to 1000 r.p.m.), temperature controls, pressure gauge, a ballast vessel, and the ability to be connected to a gas cylinder.

Ligand **4a** (10.24 µmol, 8.6 mg (Rh:L 1:2)) was added to a Schlenk tube, which was then purged with nitrogen (or argon). The mixture was dissolved in a stock solution of [Rh(acac)$(CO)_2$] in toluene (2 mg/mL, 0.65 mL, 5.12 µmol of [Rh(acac)$(CO)_2$]), the internal standard 1-methylnaphthalene (0.1 mL) was then added, followed by the addition of the designated solvent (19.35 mL). The solution was transferred *via* syringe to the pressure vessel (which had been purged with CO/$H_2$) through the injection port. CO/$H_2$(1:1) (20 bar) was added and the heating jacket set to the desired temperature while stirring. Once the desired temperature was reached (105 °C), the reaction was stirred for the required time to fully activate the catalyst (1.5 h). Then pressure was slowly released and repressurised with propene/CO/$H_2$ (20 bar). During the experiment the pressure was kept constant (20 bar) and the gas used in the reaction was replaced with syngas from the ballast vessel. Drops in pressure from the ballast vessel were recorded at 0.1 bar increments.

The reaction was then run for the time specified in **Figure S11**. After this time (5 h 29 min), stirring was stopped and the reaction was cooled by placing the vessel in a basin of cold water. The pressure was released, and the crude sample was analysed immediately by GC (in toluene) affording the corresponding aldehydes with TON= 1387 and an *iso*-selectivity of 59.5% (Previous results showed that 100% conversion at this temperature corresponds to around 1400 turnovers).

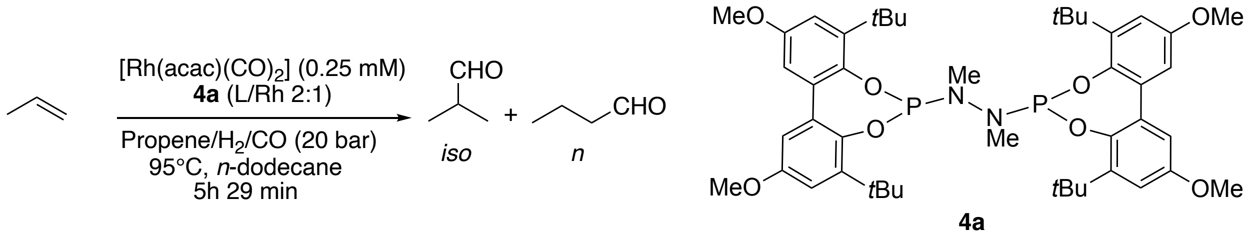


**Gas uptake (bars)**

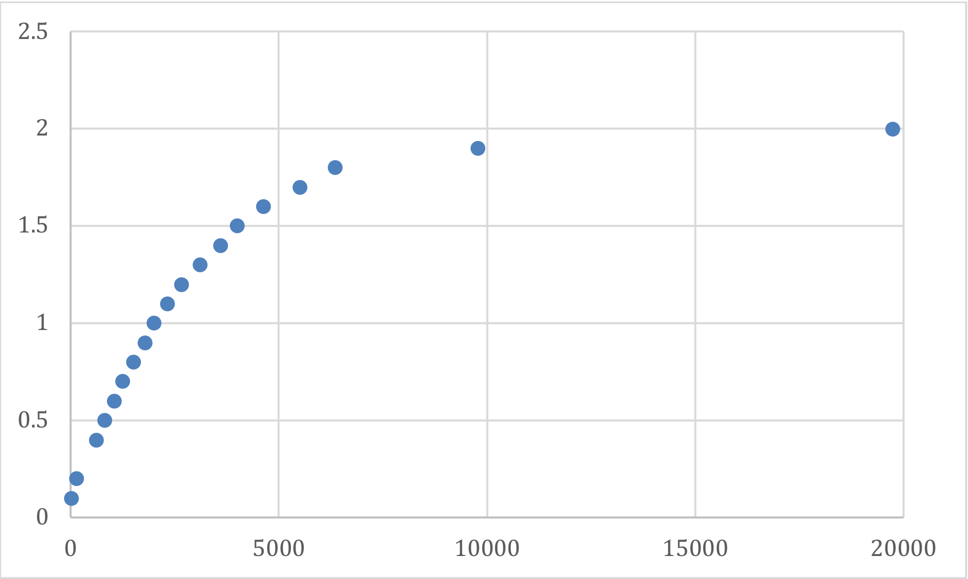


**Figure S11**. Gas uptake in the hydroformylation of propene using Ligand **4a** and [Rh(acac)(CO)$_2$]), (Rh:L 1:2). Pressure change recorded in 0.1 bar increments and time in seconds.

## 9. NMR spectroscopic investigation of the coordination mode of [Rh(4a)(CO)$_2$H].

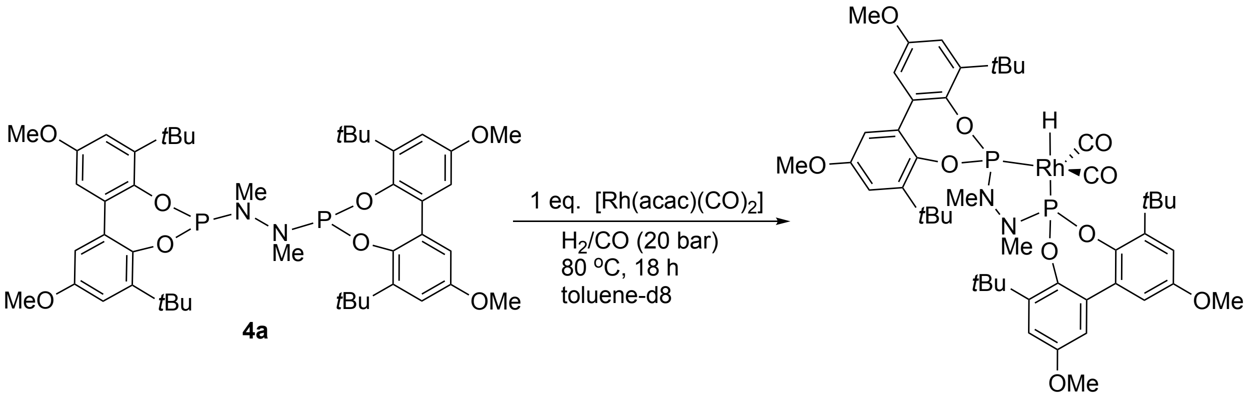


**Scheme S2**. Preparation of [Rh(**4a**)(CO)$_2$H]

### General procedure

A vial containing a stirring bar was charged with [Rh(acac)(CO)$_2$] (6.0 mg, 0.0235 mmol), **4a** (21 mg, 0.0246 mmol). The vial was sealed with a crimp cap, purged with two vacuum/argon cycles and left under an Ar atmosphere. Toluene-d8 (0.8 mL) was added, two needles were pierced into the septum of the vial cap and the vial was placed into the autoclave (The autoclave was previously purged with three vacuum/Ar cycles). The autoclave was then purged three times with syngas (50/50, CO/H$_2$), pressurised to 20 bar, immersed into an oil bath preheated at 80 °C and stirred at 700 r.p.m for the desired time (18 h). After this time, the autoclave was cooled down to room temperature by partial immersion in cold water and the pressure released to 1 atm of syngas. A suspension was observed, the complex had a low solubility in toluene at rt and DCM had to be used to aid solubility. Deuterated DCM was added till a homogenous solution was achieved. A sample was taken via syringe, introduced in a NMR tube previously purged with syngas and analysed by $^{1}$H NMR (CD$_2$Cl$_2$, toluene-d8) and $^{31}$P{$^{1}$H}-NMR (CD$_2$Cl$_2$, toluene-d8).

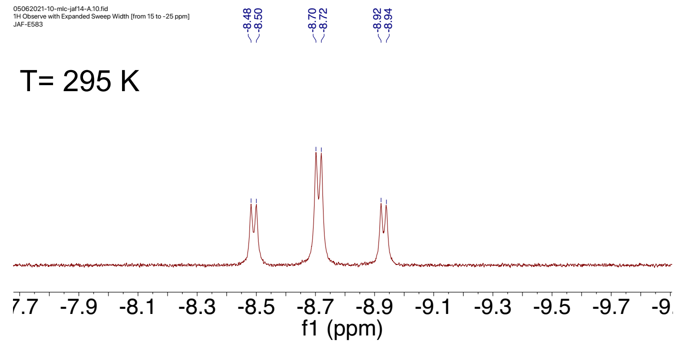


**Figure S12. $^{1}$H-NMR** spectrum of [Rh(**4a**)(CO)$_2$H] showing the hydride region at rt. Ligand **4a** presents a coordination behaviour comparable to Ph-BPE.[33] The presence of a triplet of doublets and the value of the corresponding $^{2}J_{P\text{-}H}$ coupling indicate that the ligand coordinates in an equatorial-axial (ea) fashion, in which the two P atoms are exchanging fast on the NMR time scale. **$^{1}$H NMR** (500 MHz, Toluene-$d_8$, CD$_2$Cl$_2$) δ -8.71 (td, $^{2}J_{P\text{-}H}$ = 109.7 Hz, $^{1}J_{Rh\text{-}H}$ 8.8 Hz, 1H).

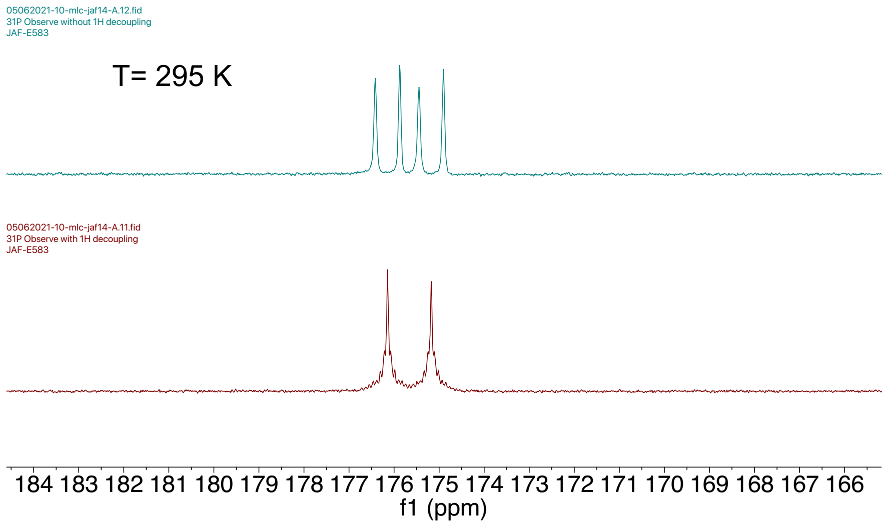


**Figure S13.** $^{31}$**P-NMR** and $^{31}$**P{$^1$H}-NMR** spectra of [Rh(**4a**)(CO)$_2$H] at rt. The presence of a double of doublets and the value of the $^2J_{P\text{-}H}$ suggest an equatorial-axial (ea) coordination mode. $^{31}$P-NMR (202 MHz, Toluene-$d_8$, $CD_2Cl_2$) δ 175.9 (dd, $^1J_{Rh\text{-}P}$ = 195.7 Hz, $^2J_{P\text{-}H}$ = 109.4 Hz). $^{31}$P{$^1$H}-NMR (202 MHz, Toluene-$d_8$, $CD_2Cl_2$) δ 175.9 (dd, $^1J_{Rh\text{-}P}$ = 195.7 Hz).

## 10. NMR spectroscopic investigation of the stability under syngas at 90 °C of [Rh(4a)(CO)$_2$H]. One week-long experiment.

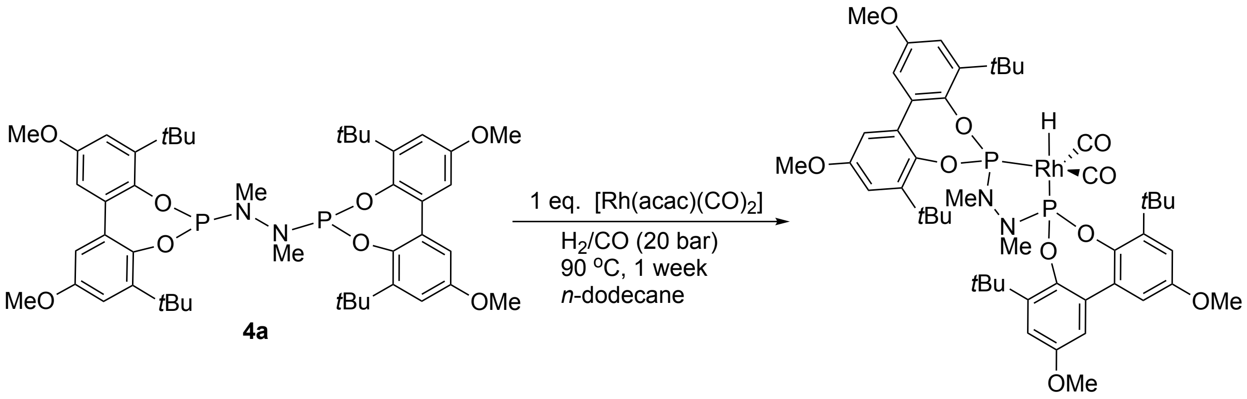


**Scheme S3**. Stability experiment with [Rh(**4a**)(CO)$_2$H]

[Rh(**4a**)(CO)$_2$H] complex was formed according to the general procedure. After 1 week there was a precipitate. A sample of the solution was taken via syringe, introduced in an NMR tube previously purged with syngas and analysed by $^1$H NMR and $^{31}$P-NMR using a benzene-d6 capillary. Both NMR showed no presence of the complex or any other major P-containing species in solution. As much as possible n-dodecane was removed from the precipitate *via* syringe and then was dissolved in $CD_2Cl_2$ (previously degassed with $H_2$/CO). The 1H and 31P NMR showed the presence in solution of the desired complex [Rh(**4a**)(CO)$_2$H] in excellent purity even after a whole week.

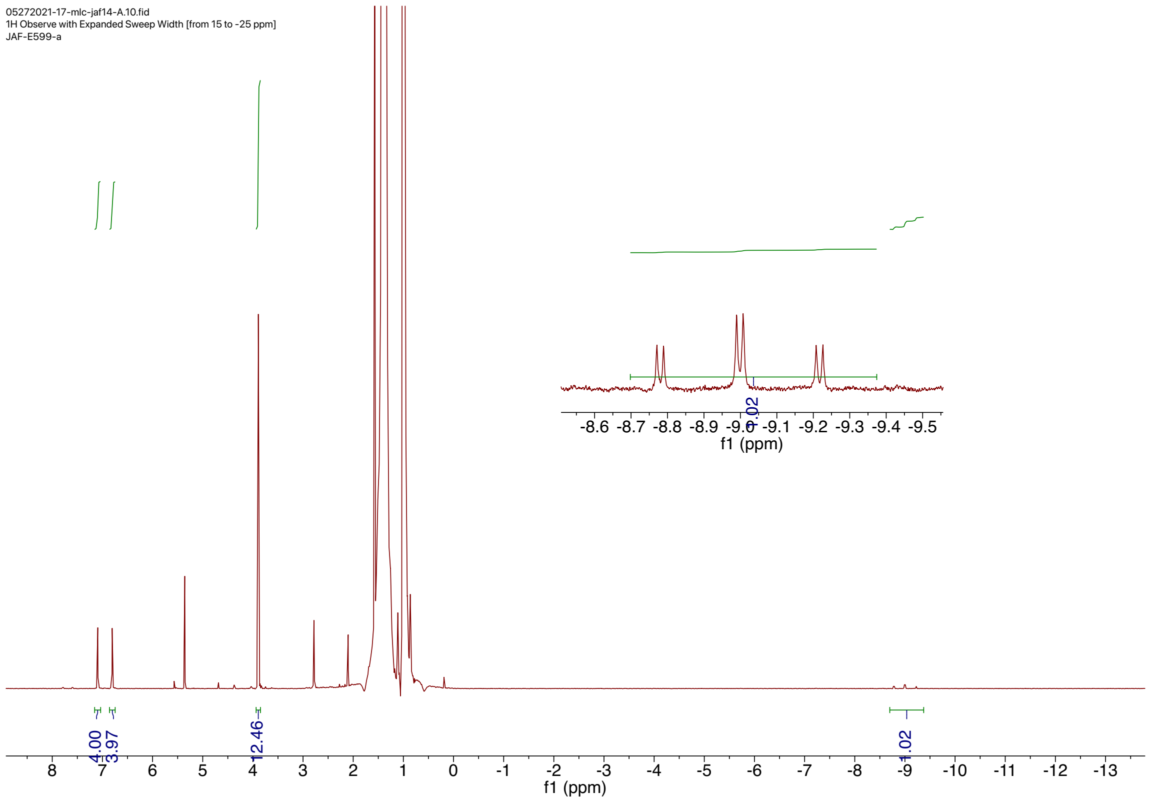


**Figure S14.** **$^{1}$H-NMR**($CD_2Cl_2$) spectrum of [Rh(**4a**)(CO)$_2$H] (precipitate) after one week stirring under syngas atmosphere at 90 °C.

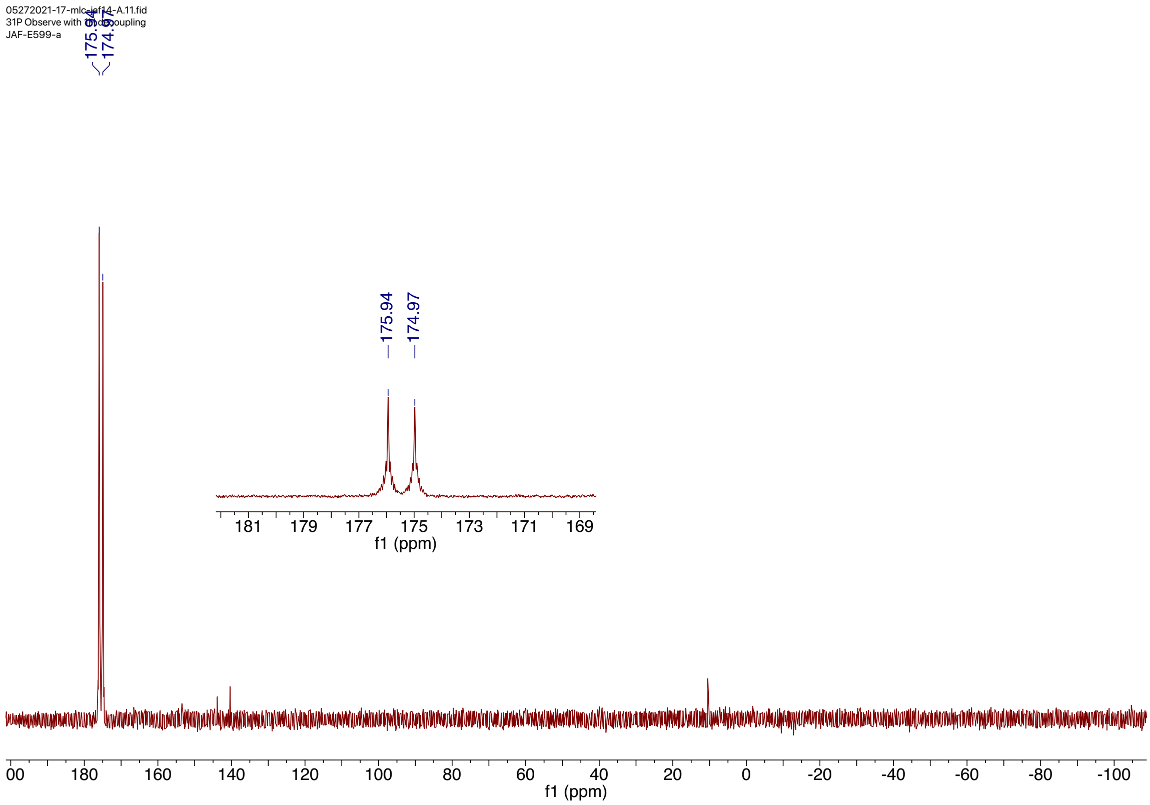


**Figure S15.** **$^{31}$P{$^{1}$H}-NMR**($CD_2Cl_2$) spectrum of [Rh(**4a**)(CO)$_2$H] (precipitate) after one week stirring under syngas atmosphere at 90 °C.

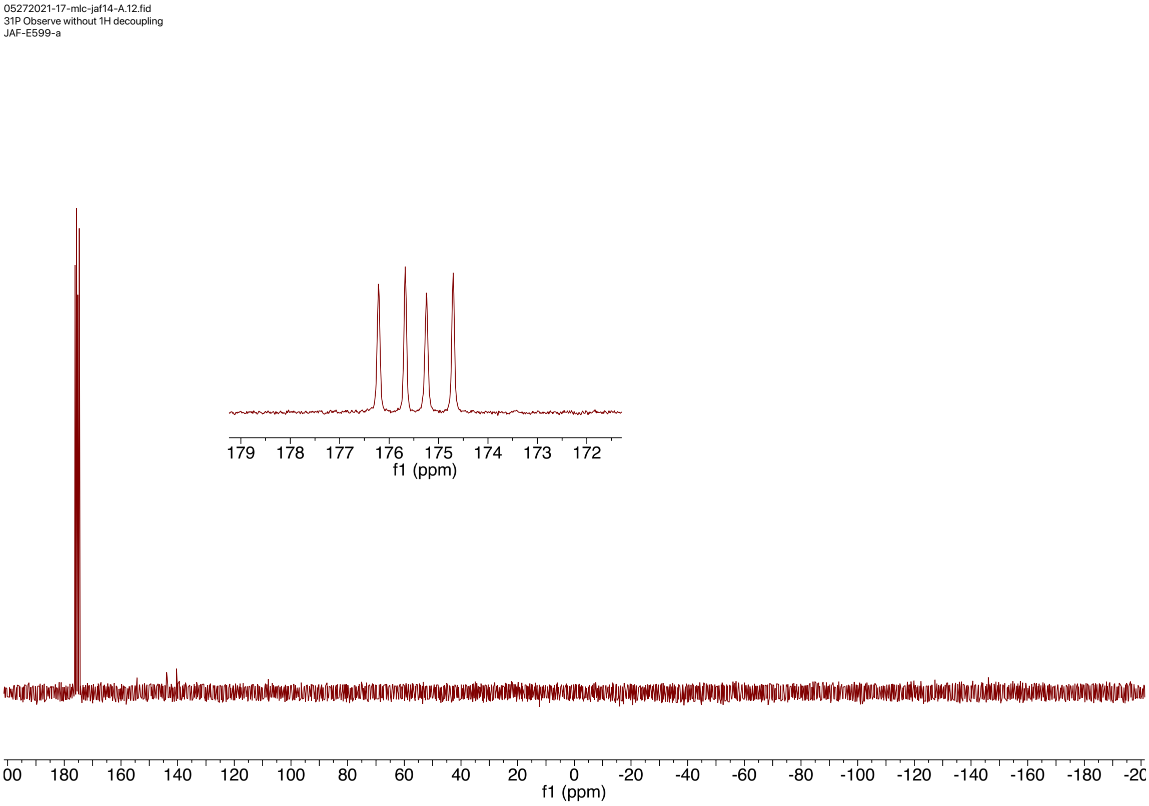


**Figure S16. $^{31}$P-NMR**($CD_2Cl_2$) spectrum of [Rh(**4a**)$(CO)_2$H] (precipitate) after one week stirring under syngas atmosphere at 90 °C

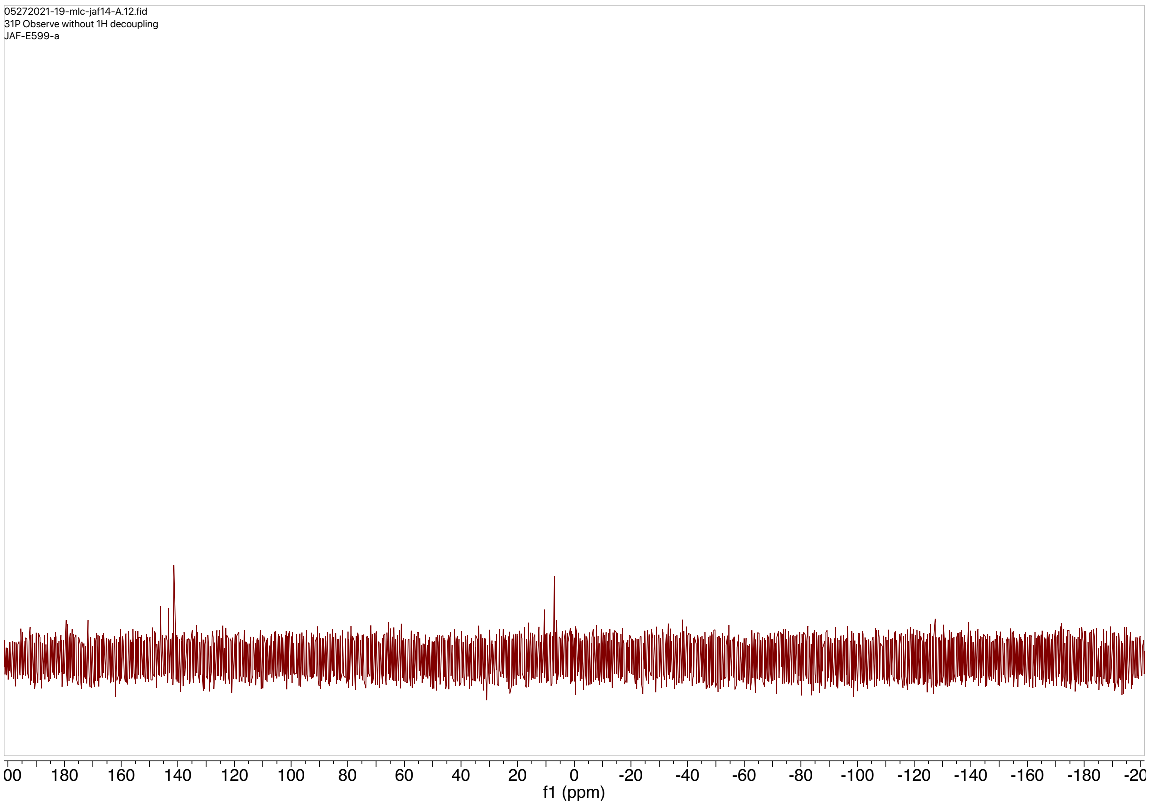


**Figure S17. $^{31}$P{$^{1}$H}-NMR** spectrum of *n*-dodecane solution from the formation [Rh(**4a**)$(CO)_2$H] after one week stirring under syngas showing no presence of the complex or any other major P-containing species in solution.

**11. Propene hydroformylation using Rh/4b, comparing selectivity before and after ageing in the presence of an aldehyde.**

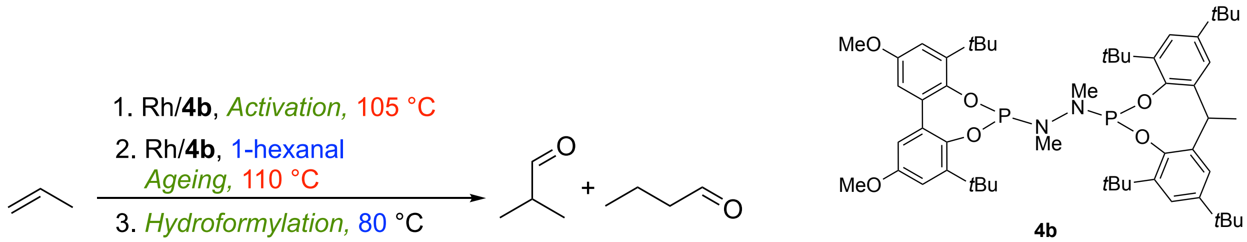


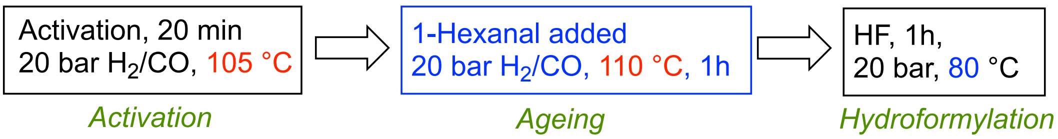


| Experiment | T(°C) | TON | T(h) | Iso(%) | *n: iso* |
|---|---|---|---|---|---|
| No ageing | 80 | 205 | 1 | 41.0 | 1.45 |
| With ageing | 80 | 182 | 1 | 40.6 | 1.47 |

If the experiments are carried out with a catalyst that is aged in hexanal before carrying out the hydroformylation, essentially the same results are observed. Thermal decomposition of Rh/**4b** to another catalysts is therefore ruled out and the difference in *iso* selectivity between 80 and 110 °C is not due to catalyst decomposition (*Iso* at 110 °C : 49.2%).

## 12. Reaction mixture from allylbenzene hydroformylation.

Reaction mixture from allyl benzene **5a** hydroformylation using **4a** and hexafluorobenzene as solvent achieving high branched aldehyde selectivity.

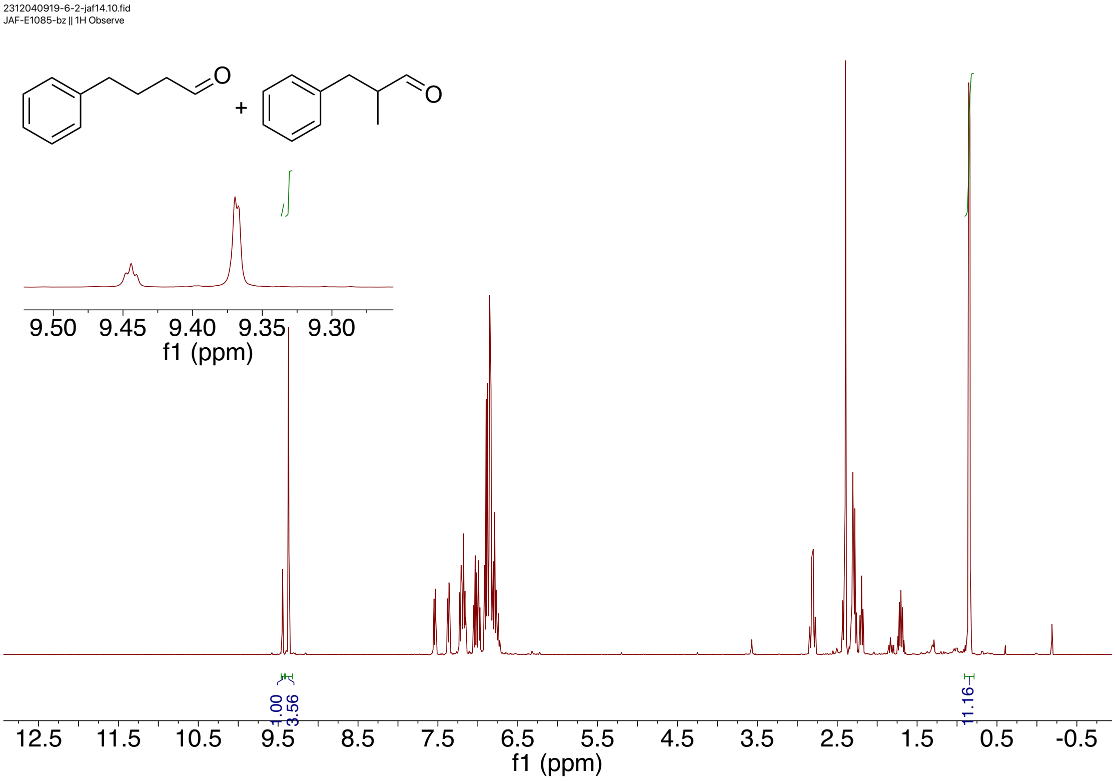


## 13. Effect of ligand 4d on propylene hydroformylation. Table S1.

| Entry[a] | Ligand | L:Rh | Solvent | ${}^{T}P$ | ${}^{P}P$ $N_2$ | $T_{act}$ °C | T °C | t h | TON | iso(%) | *n:iso* |
|---|---|---|---|---|---|---|---|---|---|---|---|
| 1 | **4d** | 2:1 | DOTP (90%) | 20 | 0 | 105 | 80 | 1 | 111 | 52.9 | 0.89 |
| 2 | **4d** | 10:1 | DOTP (90%) | 20 | 0 | 105 | 80 | 1 | 147 | 51.5 | 0.94 |
| 3 | **4d** | 2:1 | DOTP (90%) | 20 | 10 | 105 | 80 | 1 | 85 | 54.0 | 0.85 |
| 4 | **4d** | 2:1 | DOTP (90%) | 20 | 0 | 105 | 95 | 1 | 374 | 53.8 | 0.86 |
| 5 | **4d** | 2:1 | DOTP (90%) | 20 | 0 | 105 | 110 | 1 | 926 | 55.4 | 0.81 |

[a] Catalyst preformed from [Rh(acac)(CO)2] (5.12 x $10^{-3}$ mmol) and ligand (10.24 x $10^{-3}$ mmol (**4d**:Rh 2:1)) by stirring at 20 bar $CO/H_2$ at activation temperature for 20 min, in DOTP (90%) (18 mL + 2 mL toluene) and then increasing or decreasing the temperature to the required temperature prior to running reaction at time specified using propene/$CO/H_2$ in1:4.5:4.5 ratio (20 bar initial pressure). Rh concentration = 2.52 x $10^{-4}$ mol $dm^{-3}$. Product determined by GC using 1-methylnaphthalene as an internal standard.

## 14. X-ray Crystallography

X-ray diffraction data for compounds [Pt(**4a**)$Cl_2$]·2$CHCl_3$ and [Pt(**4b**)$Cl_2$]·2.5$CH_2Cl_2$ were collected using a Rigaku FR-X Ultrahigh Brilliance Microfocus RA generator/confocal optics with XtaLAB P200 diffractometer [Mo Kα radiation (λ = 0.71073 Å)]. Data for both compounds were collected (using a calculated strategy) and processed (including correction for Lorentz, polarization and absorption) using CrysAlisPro.[51] Structures were solved by dual-space methods (SHELXT[52]) and refined by full-matrix least-squares against $F^2$ (SHELXL-2019/3[53]). Non-hydrogen atoms were refined anisotropically, and hydrogen atoms were refined using a riding model. Disordered solvates in both structures were split into parts and refined with geometric and thermal restraints. The disordered methoxy group in [Pt(**4a**)$Cl_2$]·2$CHCl_3$ was modelled in two parts with thermal restraints. One dichloromethane solvate in [Pt(**4b**)$Cl_2$)]·2.5$CH_2Cl_2$ was split over an inversion point and refined with occupancy fixed at 0.5, geometric restraints, and $U_{eq}$ of both chlorides constrained to each other due to overlap with their symmetry generated counterparts. All calculations were performed using the Olex2[54] interface. Selected crystallographic data are presented in **Table S2**. CCDC 2429273 and 2429274 contains the supplementary crystallographic data for this paper. These data can be obtained free of charge from The Cambridge Crystallographic Data Centre via www.ccdc.cam.ac.uk/structures.

**Table S2.** Selected crystallographic data.

| | [Pt(**4a**)$Cl_2$]·2$CHCl_3$ | [Pt(**4b**)$Cl_2$]·2.5$CH_2Cl_2$ |
|---|---|---|
| formula | $C_{48}H_{64}N_2O_8P_2Cl_8Pt$ | $C_{56.5}H_{83}N_2O_6P_2Cl_7Pt$ |
| fw | 1337.64 | 1391.42 |
| temperature [K] | 120 | 100 |
| crystal description | Colourless plate | Colourless block |
| crystal size [mm$^3$] | 0.18 × 0.05 × 0.01 | 0.12 × 0.08 × 0.03 |
| space group | $P2_1/n$ | $P2_1/c$ |
| $a$ [Å] | 15.2095(3) | 16.4901(2) |
| $b$ [Å] | 32.4868(7) | 22.3697(2) |
| $c$ [Å] | 23.4987(5) | 18.2168(2) |
| $\beta$ [°] | 94.881(2) | 113.1739(15) |
| vol [Å]$^3$ | 11568.8(4) | 6177.61(14) |
| $Z$ | 8 | 4 |
| $\rho$ (calc) [g/cm$^3$] | 1.536 | 1.496 |
| $\mu$ [mm$^{-1}$] | 2.900 | 2.674 |
| F(000) | 5392 | 2844.0 |

| | | |
|---|---|---|
| reflections collected | 146052 | 129200 |
| independent reflections ($R_{int}$) | 26996 (0.0980) | 14830 (0.0392) |
| parameters, restraints | 1401, 195 | 730, 65 |
| GooF on $F^2$ | 1.021 | 1.044 |
| $R_1$ [$I > 2\sigma(I)$]s | 0.0493 | 0.0220 |
| $wR_2$ (all data) | 0.1106 | 0.0497 |
| largest diff. peak/hole [e/Å$^3$] | 1.48/-1.44 | 0.57/-1.00 |

## 15. HPIR spectroscopic activation study of phosphonites 3b and 3c and phosphoramidites 4a-4d with [Rh(acac)(CO)2].

**Figure S18.** HPIR spectra of **[Rh(*S,S*)-3b)(CO)2H]**. Conditions: Rh:L = 1:1.25 ($C_{Rh}$ 1 mM in dodecane), T = 75 °C. Full activation achieved after 60 min.

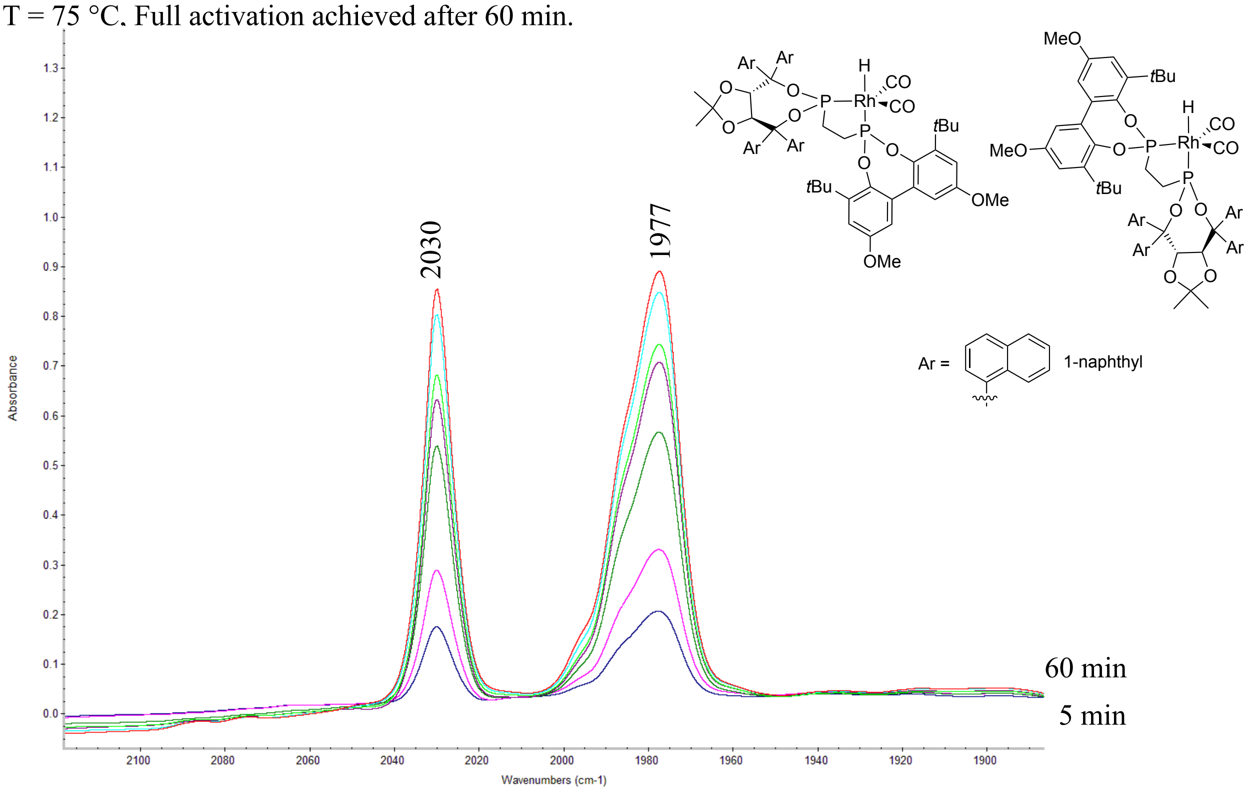


**Figure S19.** HPIR spectra of **[Rh(3c)(CO)2H]**. Conditions: Rh:L = 1:1.25 ($C_{Rh}$ 1 mM in dodecane), T = 75 °C, P= 20 bar, CO:$H_2$ 1:1. Full activation achieved after 45 min.

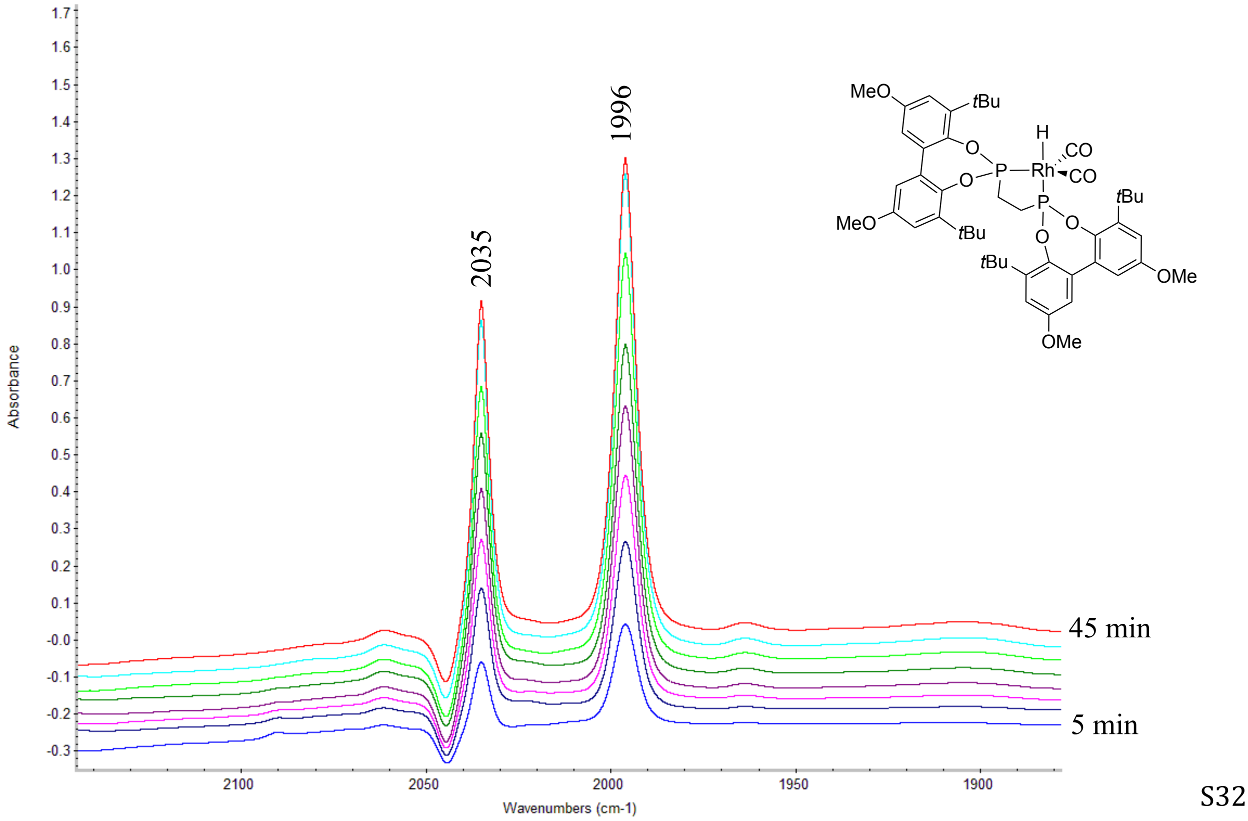

**Figure S20.** HPIR spectra of **[Rh(4a)(CO)$_2$H]**. Conditions: Rh:L = 1:1.25 ($C_{Rh}$ 1 mM in dodecane), T = 105 °C, P= 20 bar, CO:$H_2$ 1:1. Full activation achieved after 1.5h min.

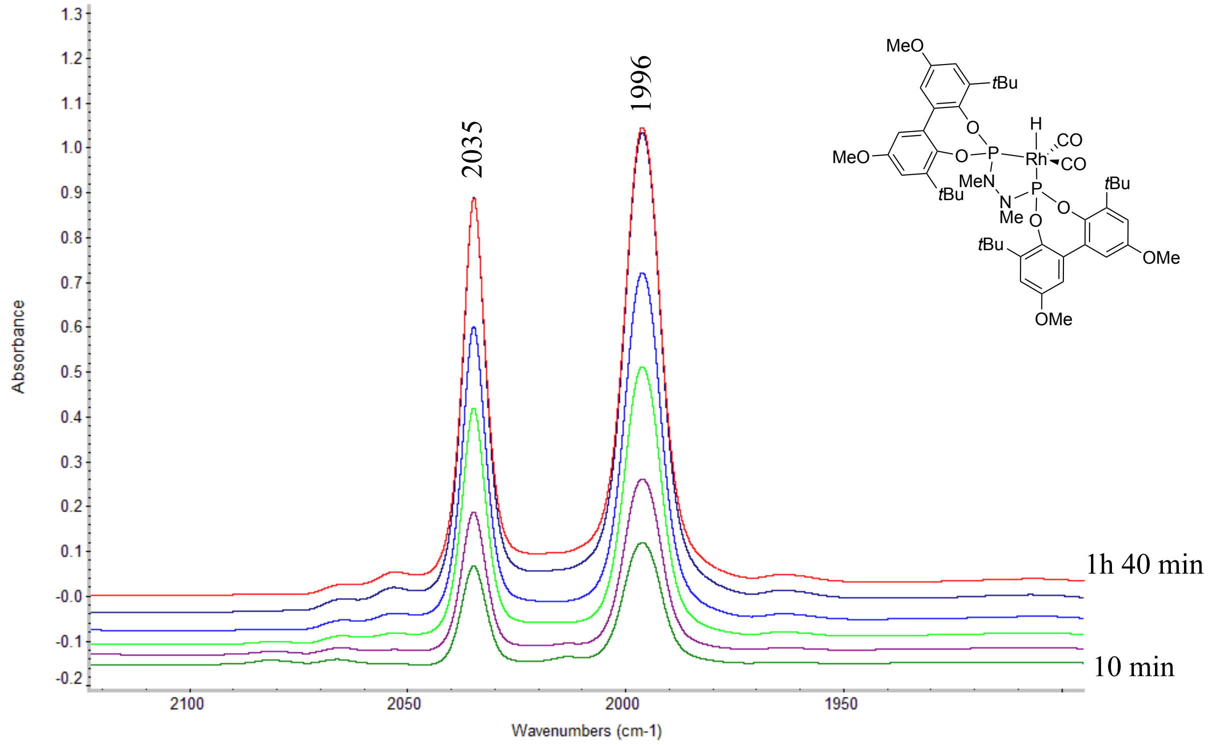


**Figure S21.** HPIR spectra of **[Rh(4a)(CO)$_2$H]** showing the thermal stability of the complex after 24h. Conditions: Rh:L = 1:1.25 ($C_{Rh}$ 1 mM in dodecane), T = 90 °C, P= 20 bar, CO:$H_2$ 1:1.

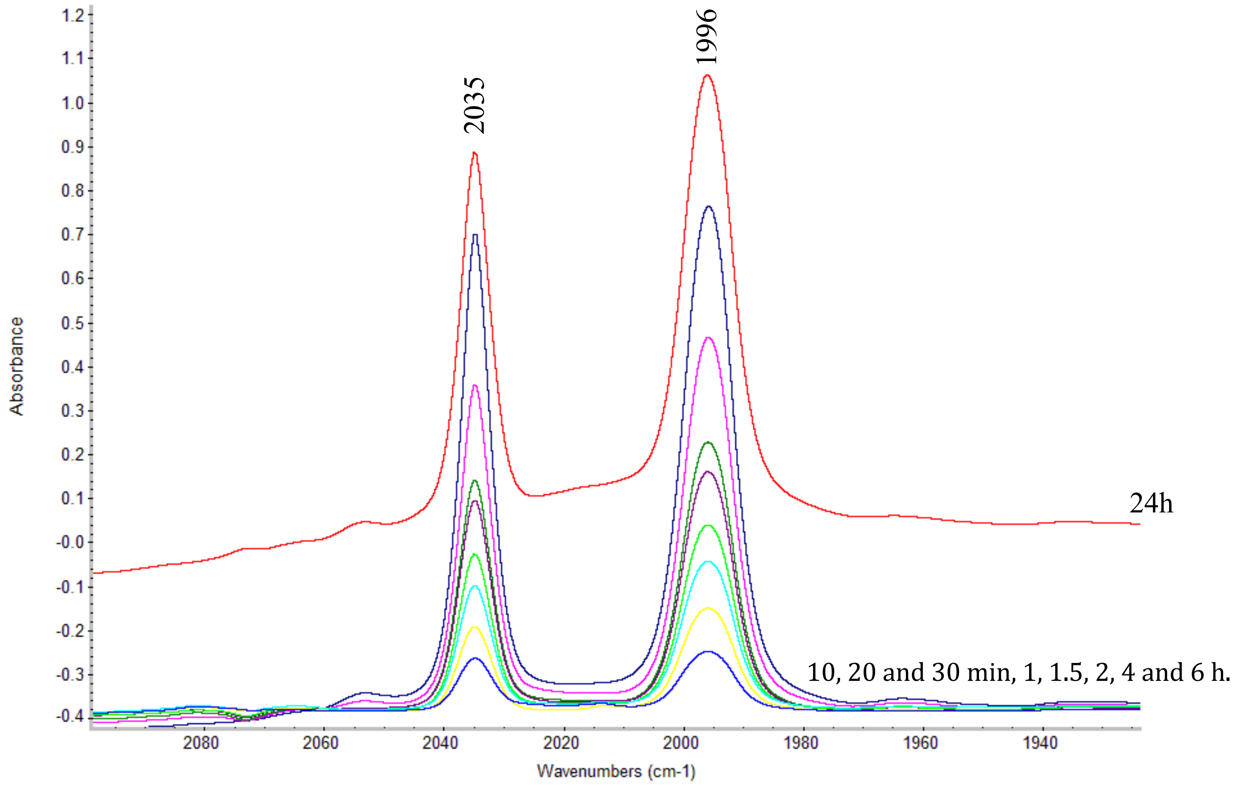

**Figure S22.** HPIR spectra of **[Rh(4b)(CO)$_2$H]**. Conditions: Rh:L = 1:1.25 ($C_{Rh}$ 1 mM in dodecane), T = 105 °C, P= 20 bar, CO:$H_2$ 1:1. Full activation achieved after 30 min.

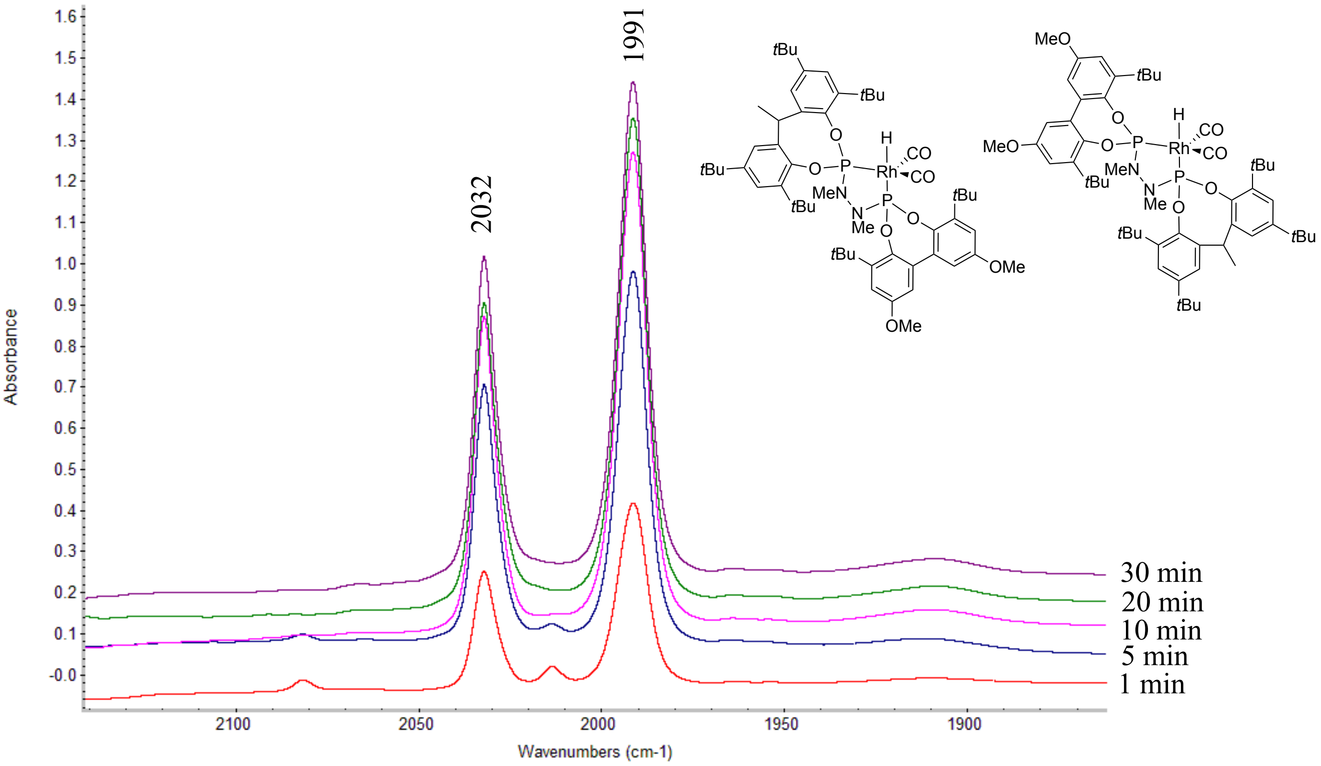


**Figure S23.** HPIR spectra of **[Rh(4c)(CO)$_2$H]**. Conditions: Rh:L = 1:1.25 ($C_{Rh}$ 1 mM in dodecane), T = 105 °C, P= 20 bar, CO:$H_2$ 1:1. Formation of Rh clusters observed after 5 min.

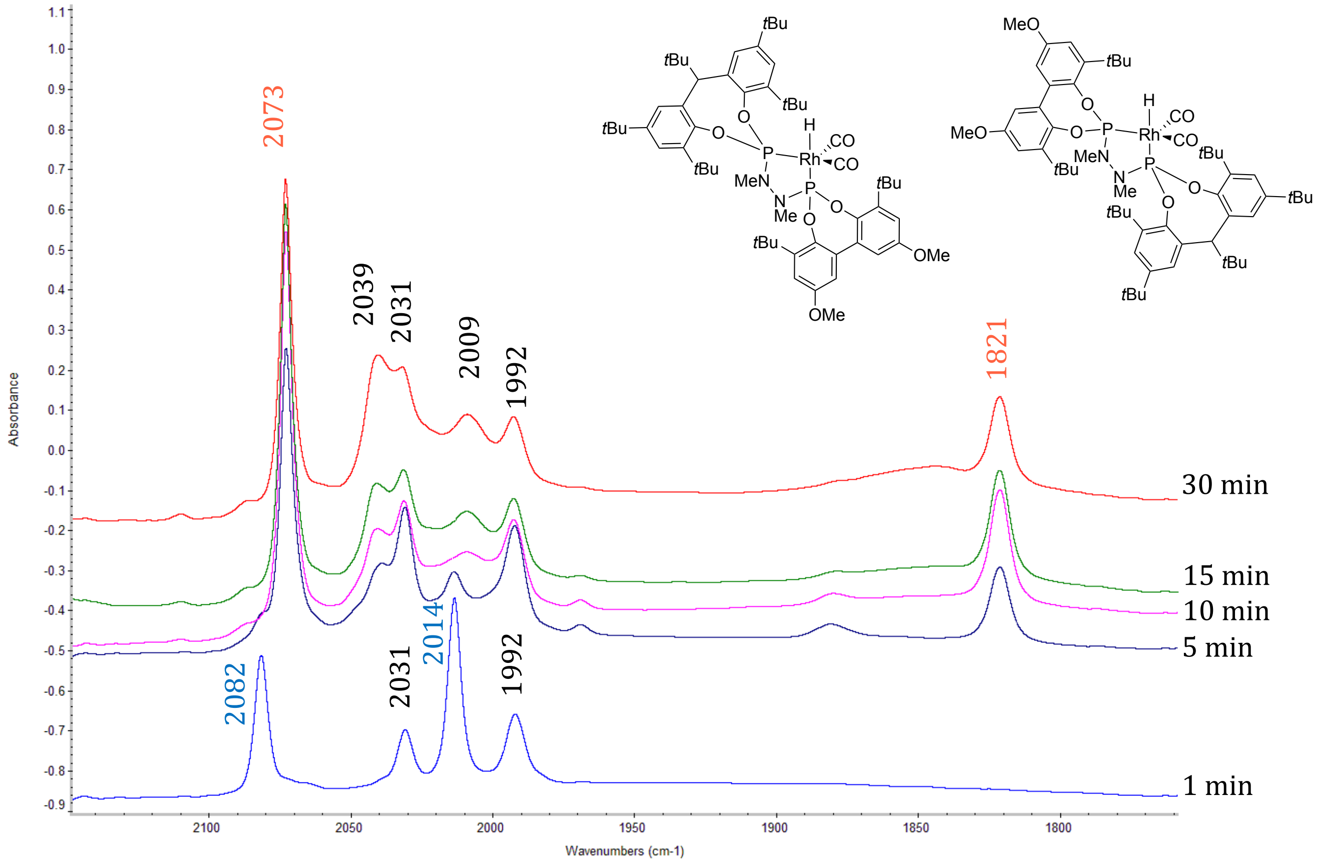

**Figure S24.** HPIR spectra of **[Rh(4d)(CO)$_2$H]**. Conditions: Rh:L = 1:1.25 ($C_{Rh}$ 1 mM in dodecane), T = 105 °C, P= 20 bar, CO:$H_2$ 1:1. Full activation achieved after 20  min.

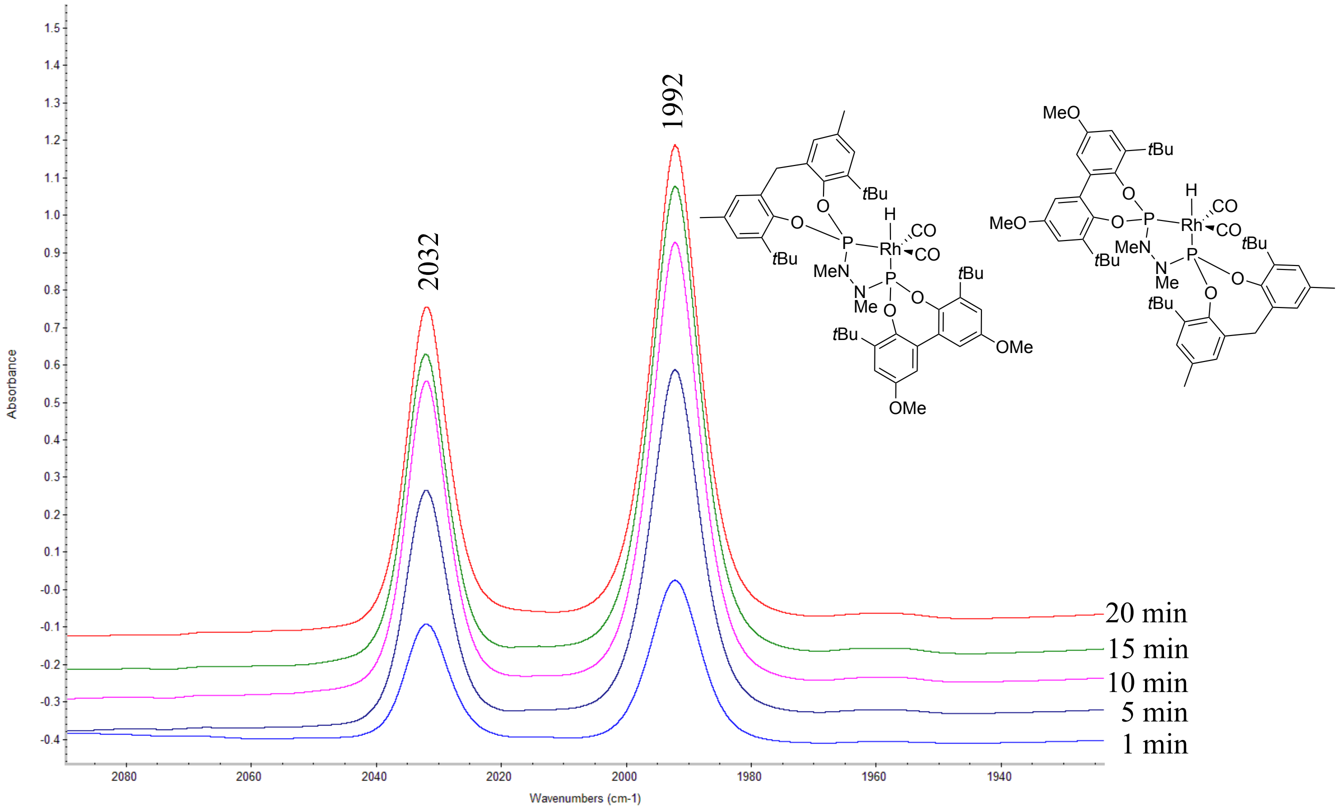

## 16. NMR spectra.

4,8-di-*tert*-butyl-6-(2-((3a*R*,6*R*)-2,2-dimethyl-4,4,8,8-tetra(naphthalen-1-yl)tetrahydro-[1,3]dioxolo[4,5-*e*][1,3,2]dioxaphosphepin-6-yl)ethyl)-2,10-dimethoxydibenzo[*d*,*f*][1,3,2]dioxaphosphepine, **3b.** $^{1}H$ NMR (top), $^{13}C$ NMR (bottom), $^{31}P\{^{1}H\}$NMR (top next page), HSQC (bottom next page)

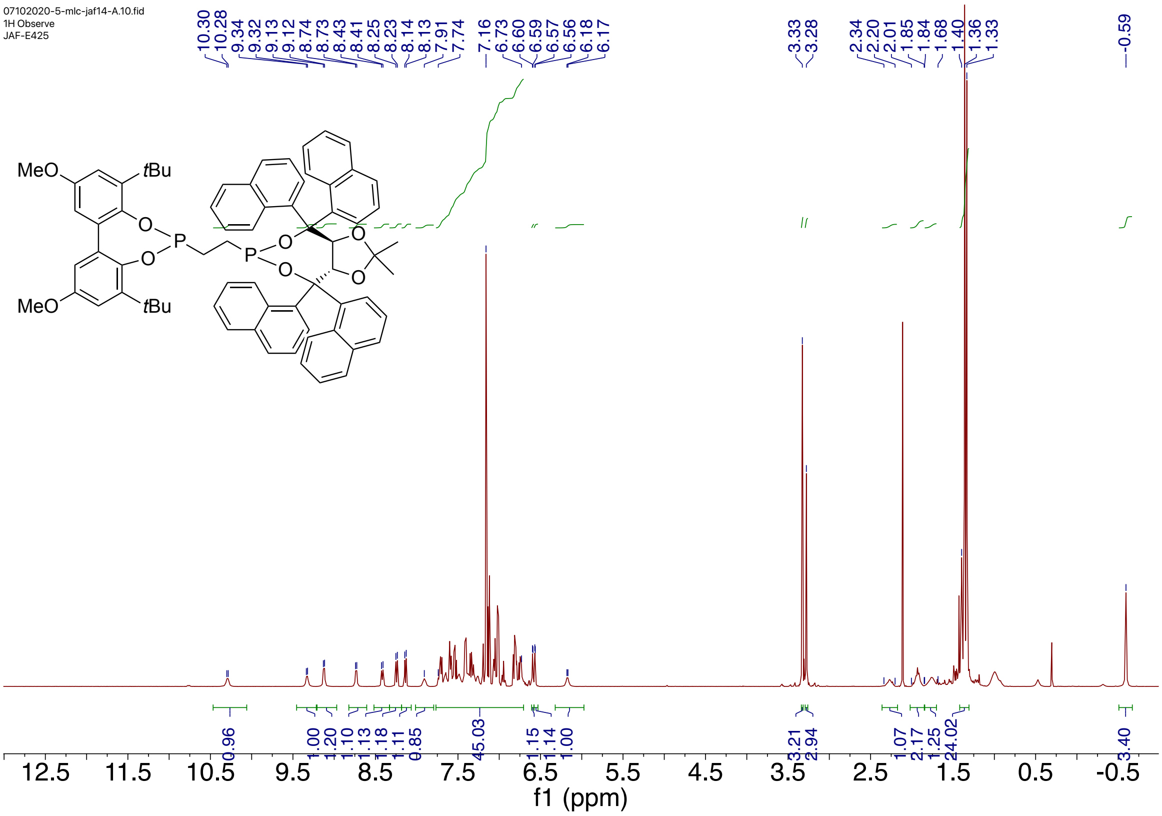

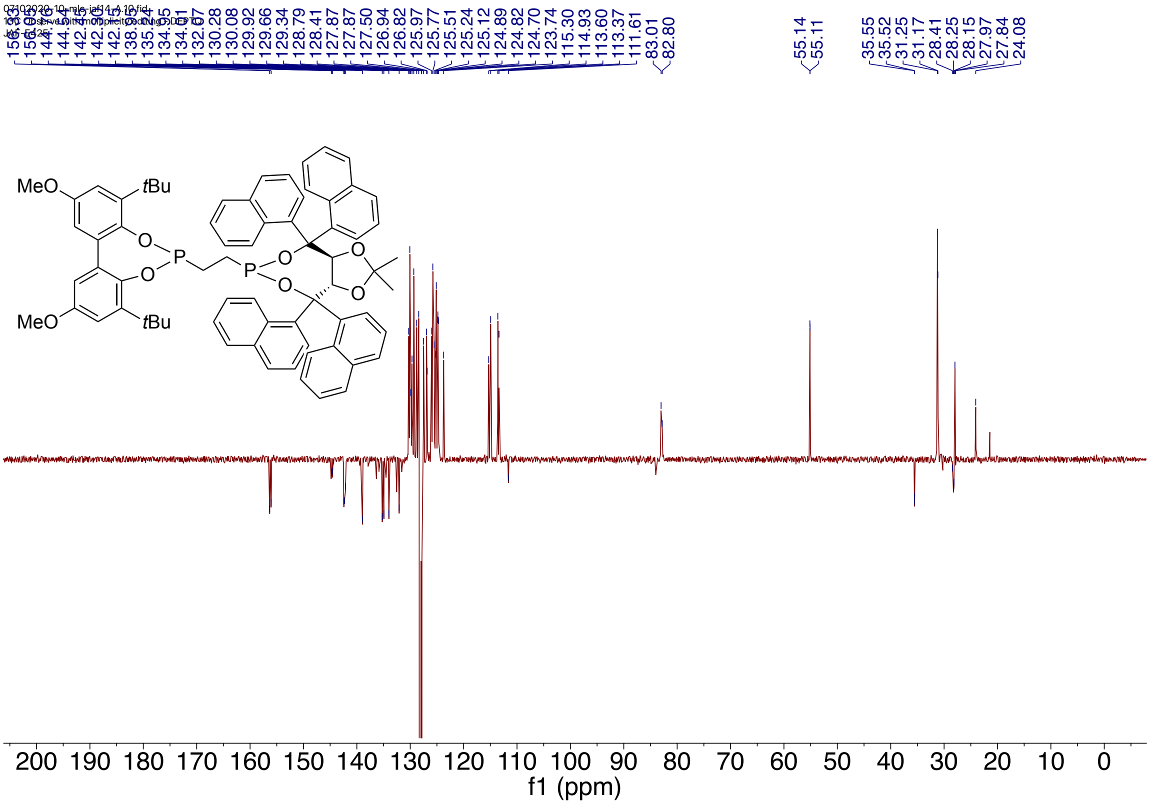

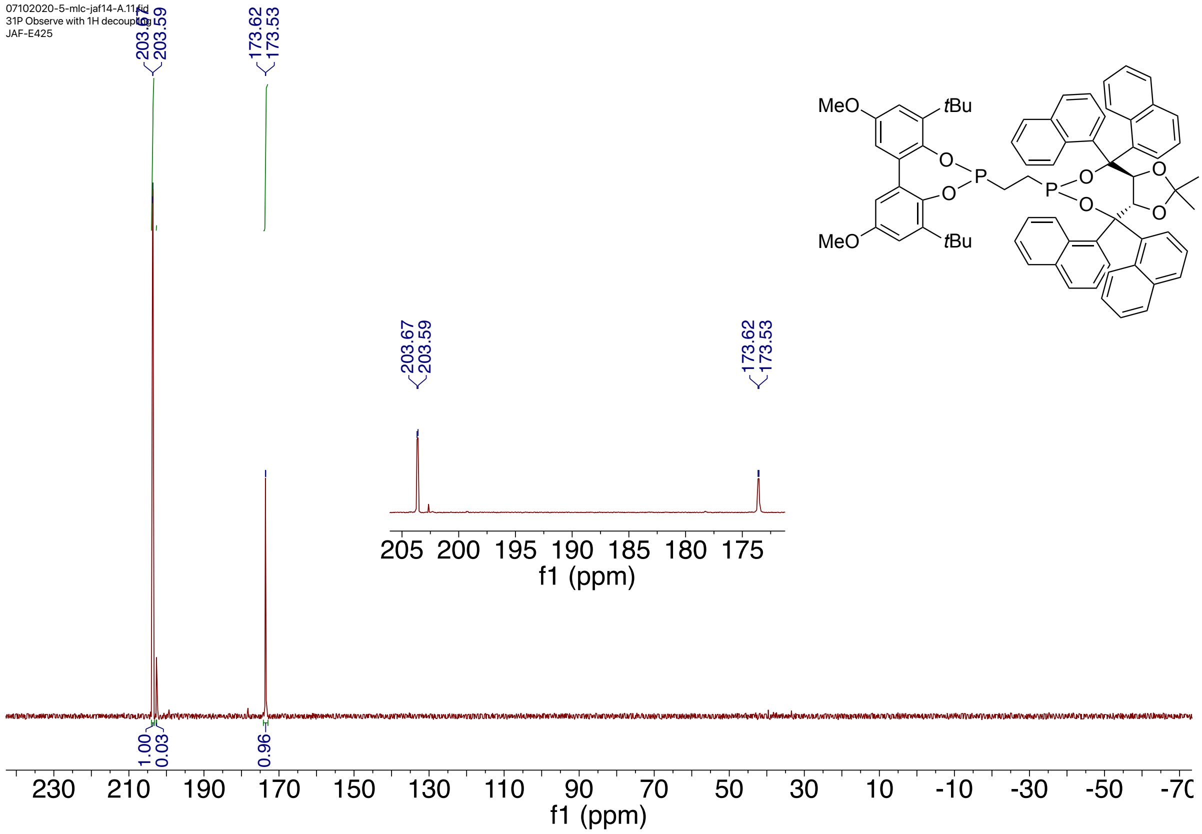
07102020-5-mlc-jaf14-A.11.fid
31P Observe with 1H decoupling
JAF-E425
203.67
203.59
173.62
173.53
1.00
0.03
0.96
f1 (ppm)
MeO
tBu
MeO
tBu
O
P
O

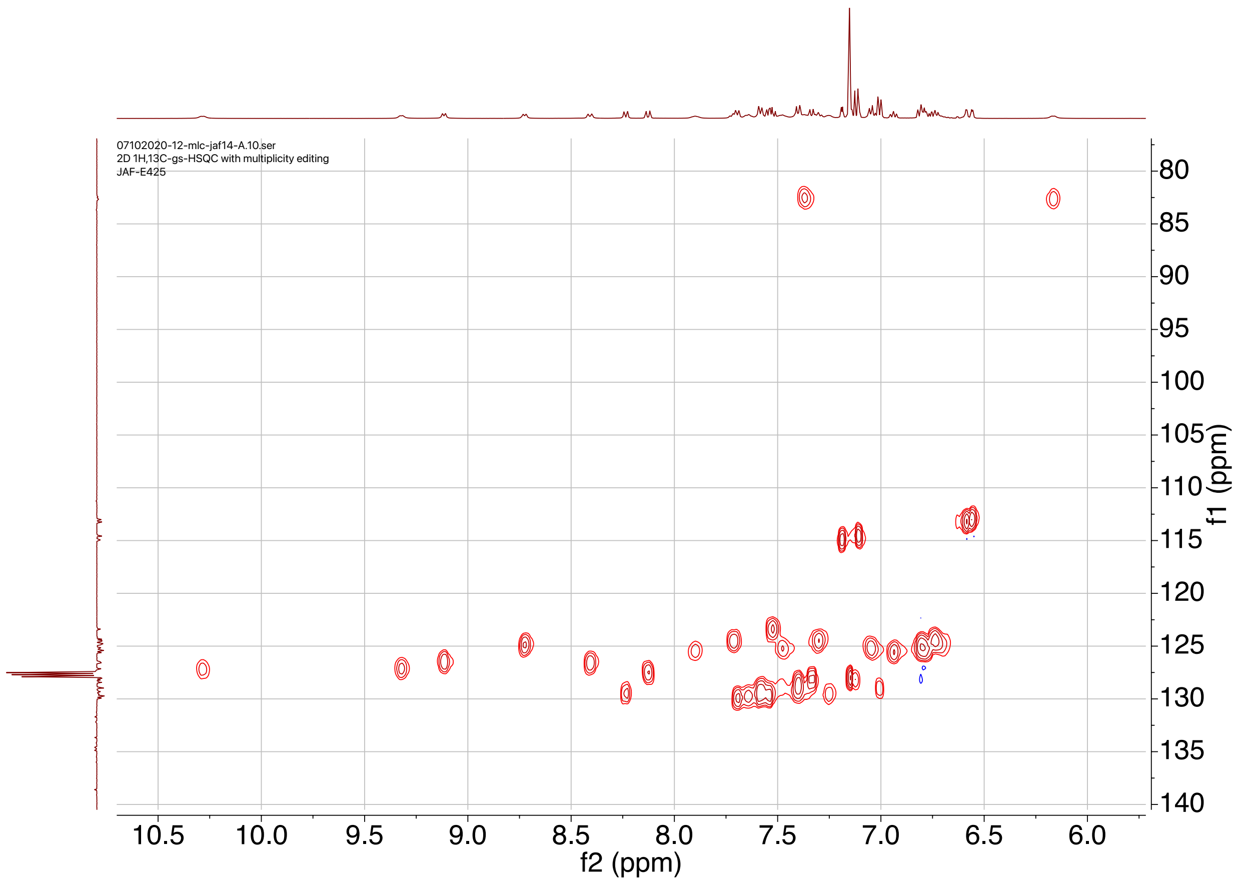
07102020-12-mlc-jaf14-A.10.ser
2D 1H,13C-gs-HSQC with multiplicity editing
JAF-E425
f2 (ppm)
f1 (ppm)

41,2-bis(4,8-di-*tert*-butyl-2,10-dimethoxydibenzo[*d*,*f*][1,3,2]dioxaphosphepin-6-yl)-1,2-dimethylhydrazine, **4a.** $^{1}$H NMR (top), $^{13}$C NMR (bottom), $^{31}$P{$^{1}$H}NMR (top next page).

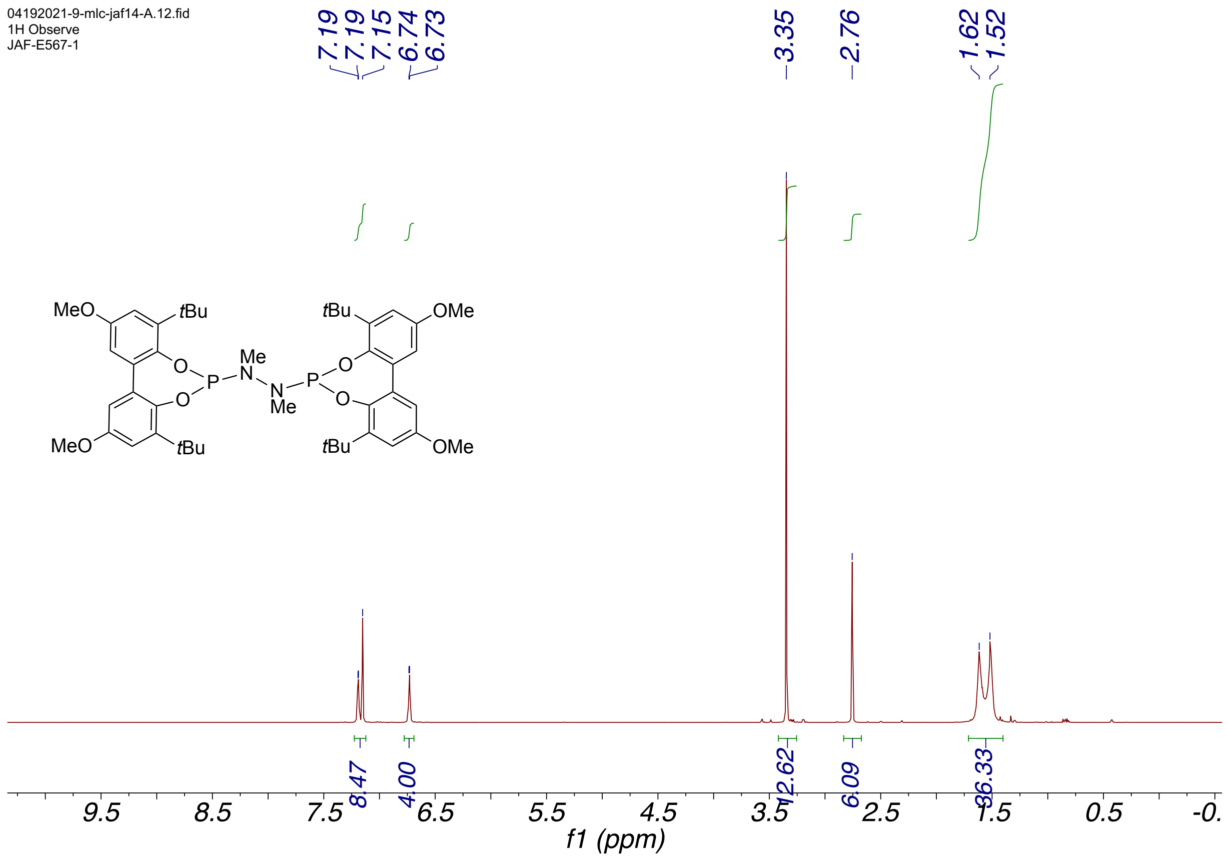


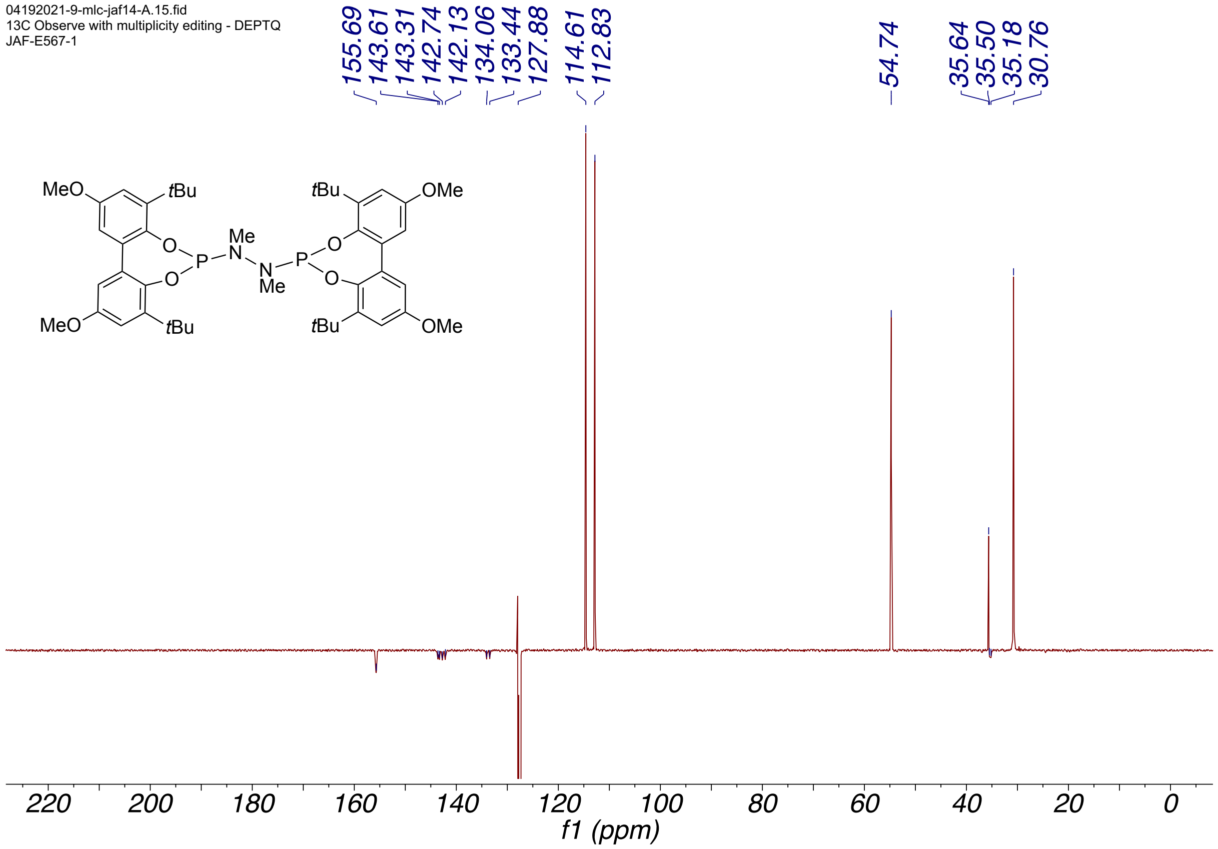

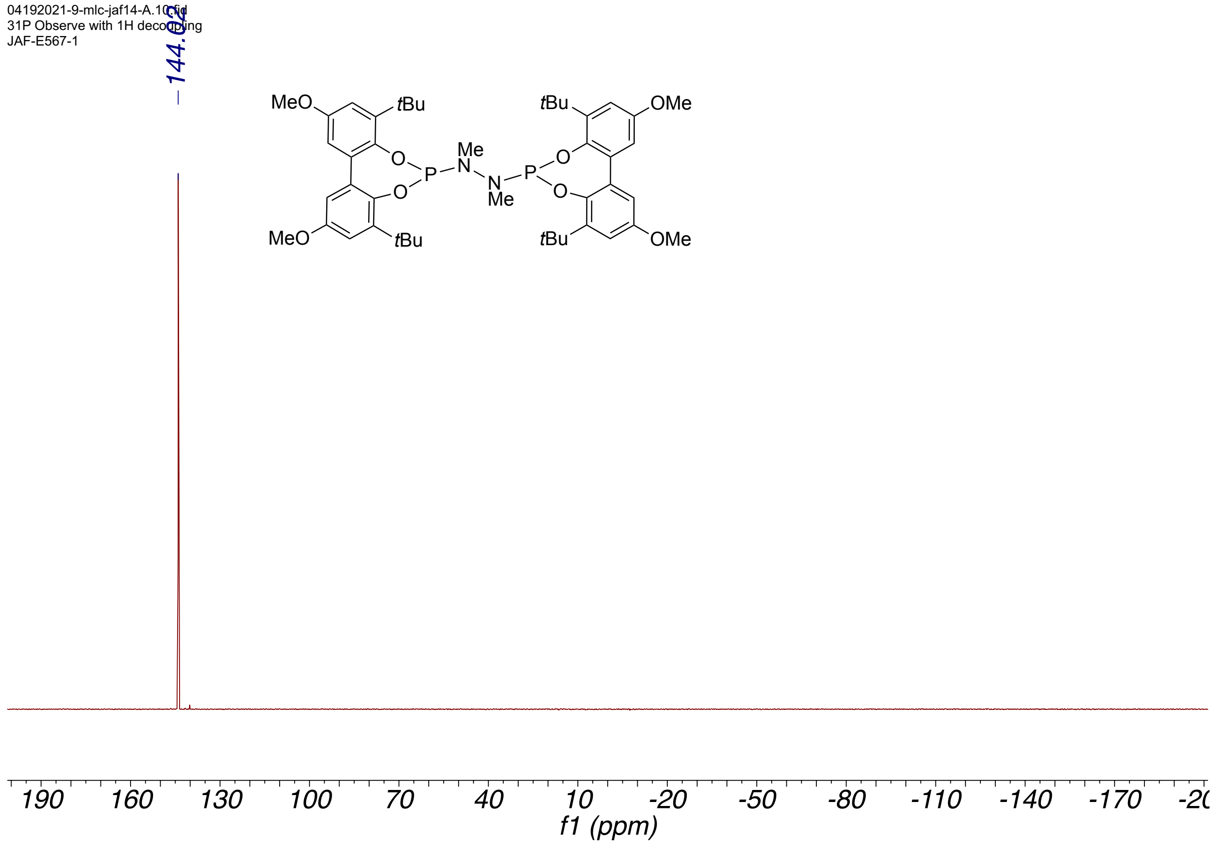


1-(4,8-di-*tert*-butyl-2,10-dimethoxydibenzo[*d*,*f*][1,3,2]dioxaphosphepin-6-yl)-1,2-dimethyl-2-(2,4,8,10-tetra-*tert*-butyl-12-methyl-12*H*-dibenzo[*d*,*g*][1,3,2]dioxaphosphocin-6-yl)hydrazine, **4b**. $^{1}$H NMR (top), $^{13}$C NMR (bottom), $^{31}$P{$^{1}$H}NMR (top next page).

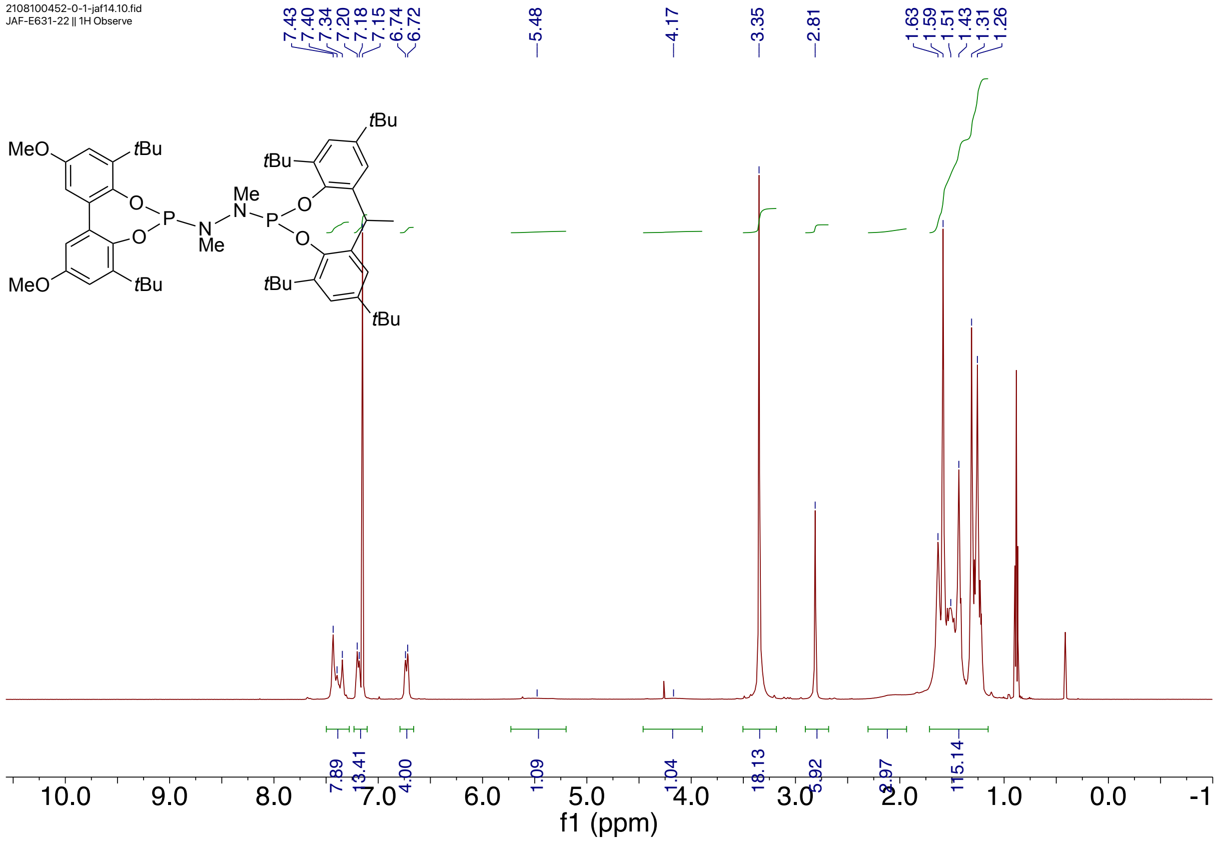

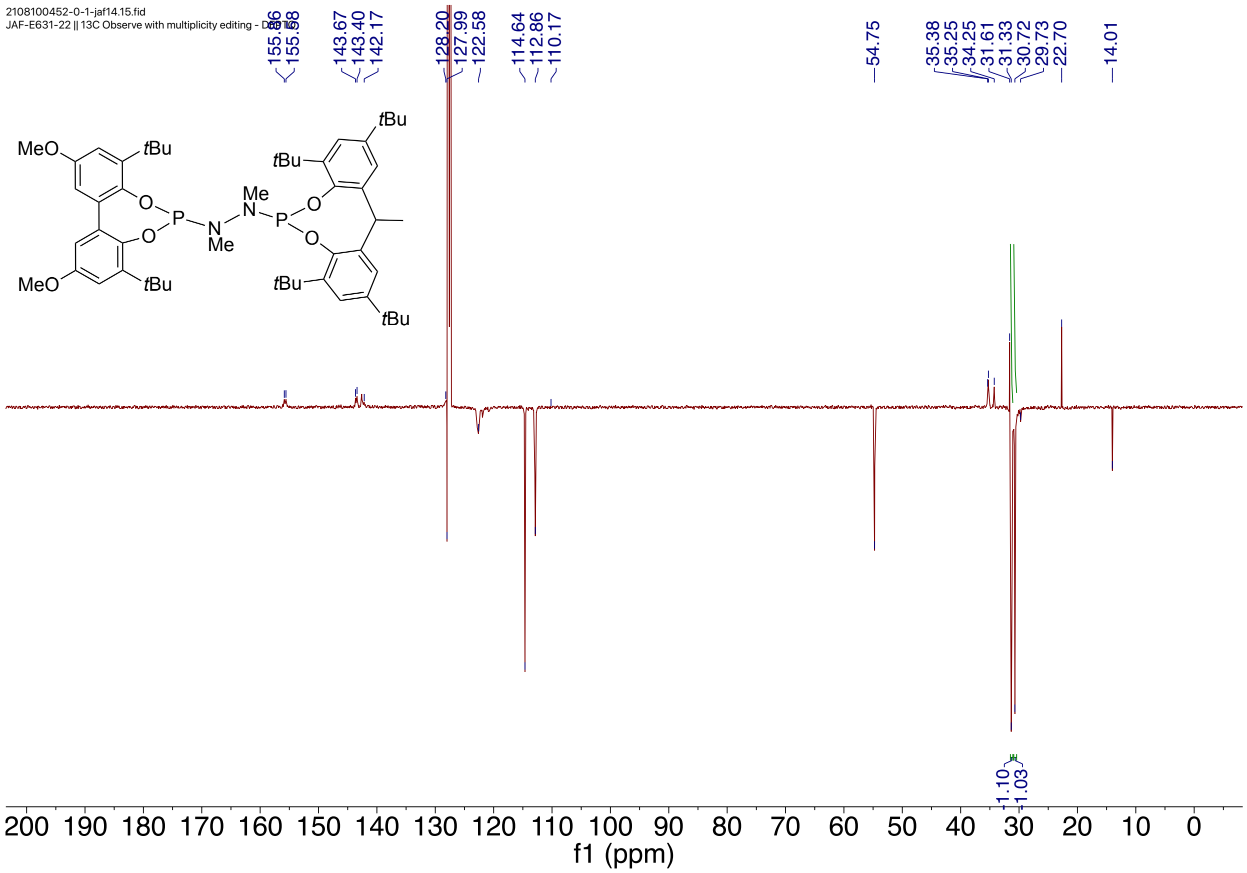
2108100452-0-1-jaf14.15.fid
JAF-E631-22 || 13C Observe with multiplicity editing - DEPTQ
155.66
155.58
143.67
143.40
142.17
128.20
127.99
122.58
114.64
112.86
110.17
54.75
35.38
35.25
34.25
31.61
31.33
30.72
29.73
22.70
14.01
MeO
tBu
Me
N
P
O
Me
1.10
1.03
f1 (ppm)

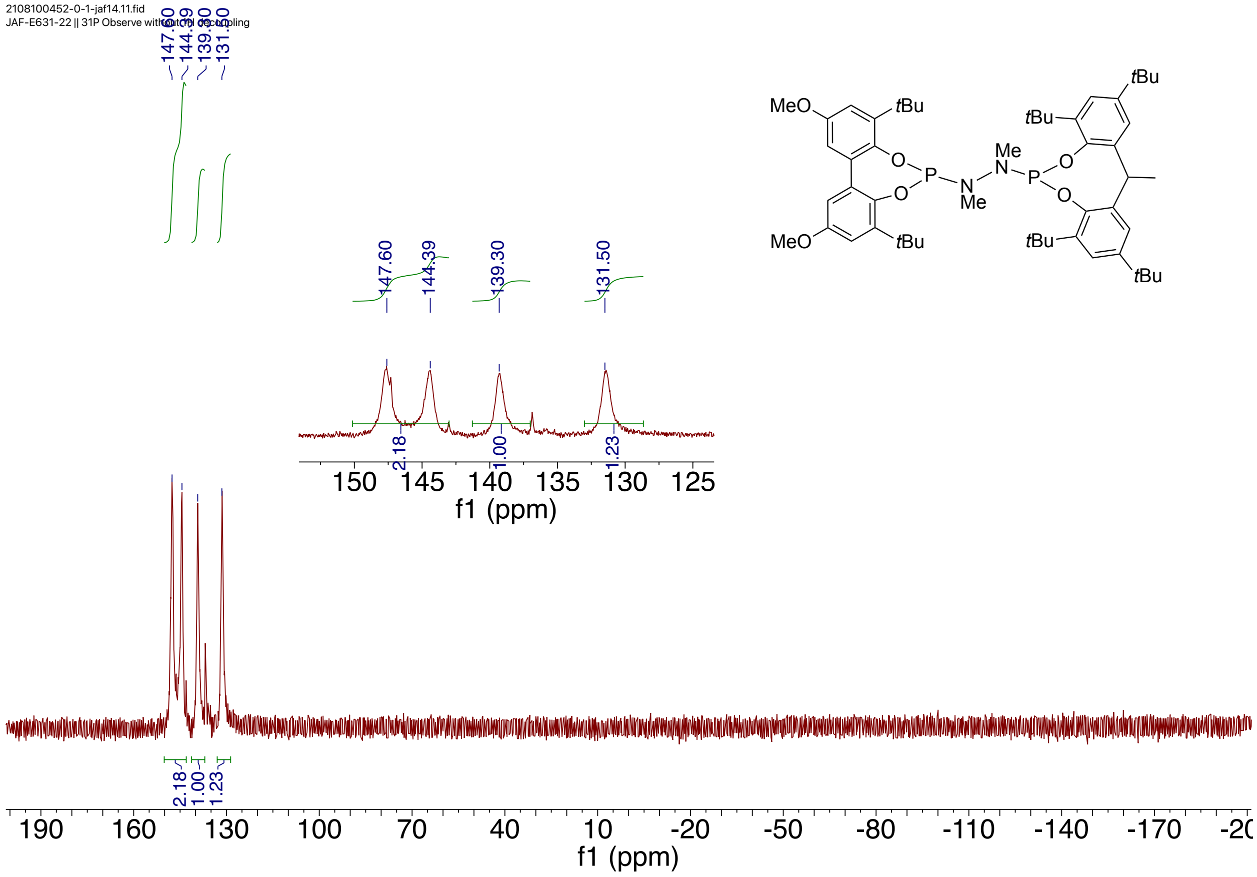
2108100452-0-1-jaf14.11.fid
JAF-E631-22 || 31P Observe with proton decoupling
147.60
144.39
139.30
131.50
2.18
1.00
1.23
f1 (ppm)
MeO
tBu
Me
N
P
O

1-(4,8-di-*tert*-butyl-2,10-dimethoxydibenzo[*d,f*][1,3,2]dioxaphosphepin-6-yl)-1,2-dimethyl-2-(2,4,8,10-tetramethyl-12*H*-dibenzo[*d,g*][1,3,2]dioxaphosphocin-6-yl)hydrazine, **4d**. $^{1}$H NMR (top), $^{13}$C NMR (bottom), $^{31}$P{$^{1}$H}NMR (top next page).

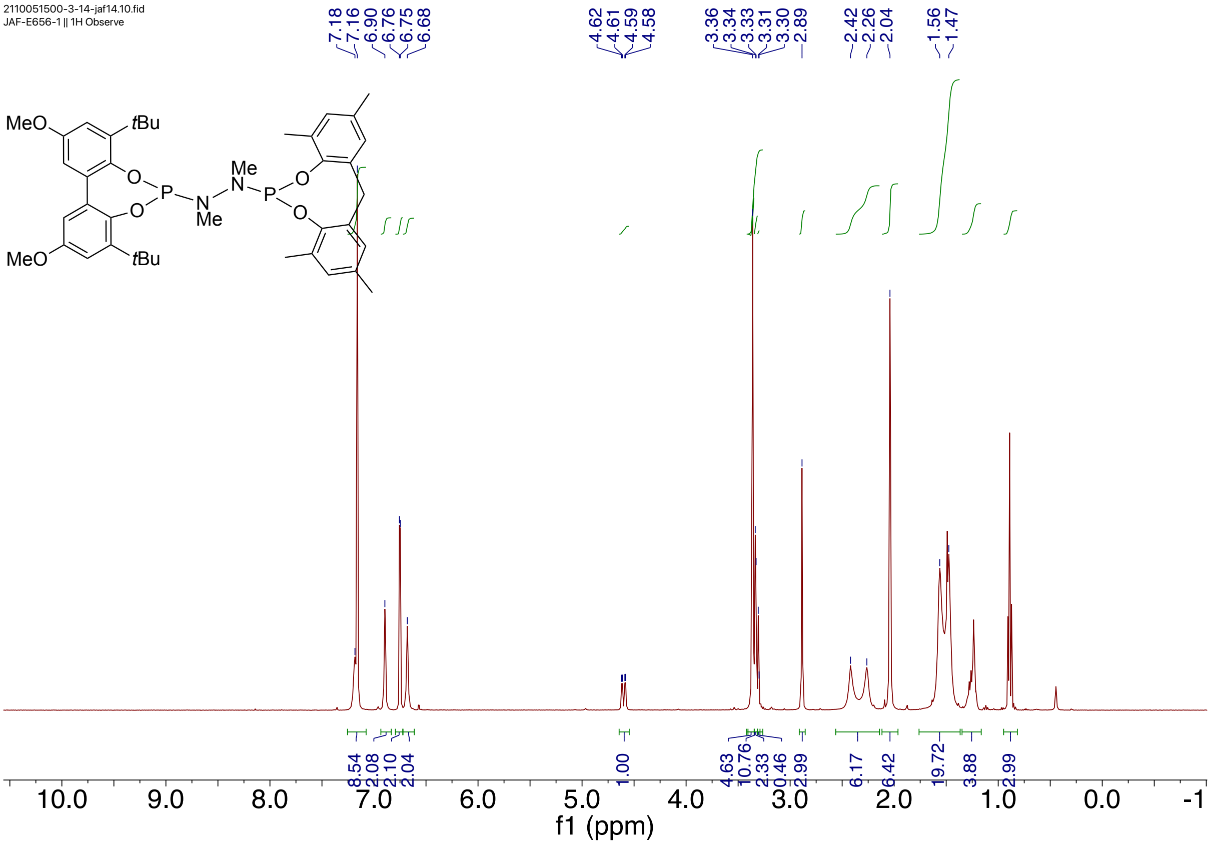


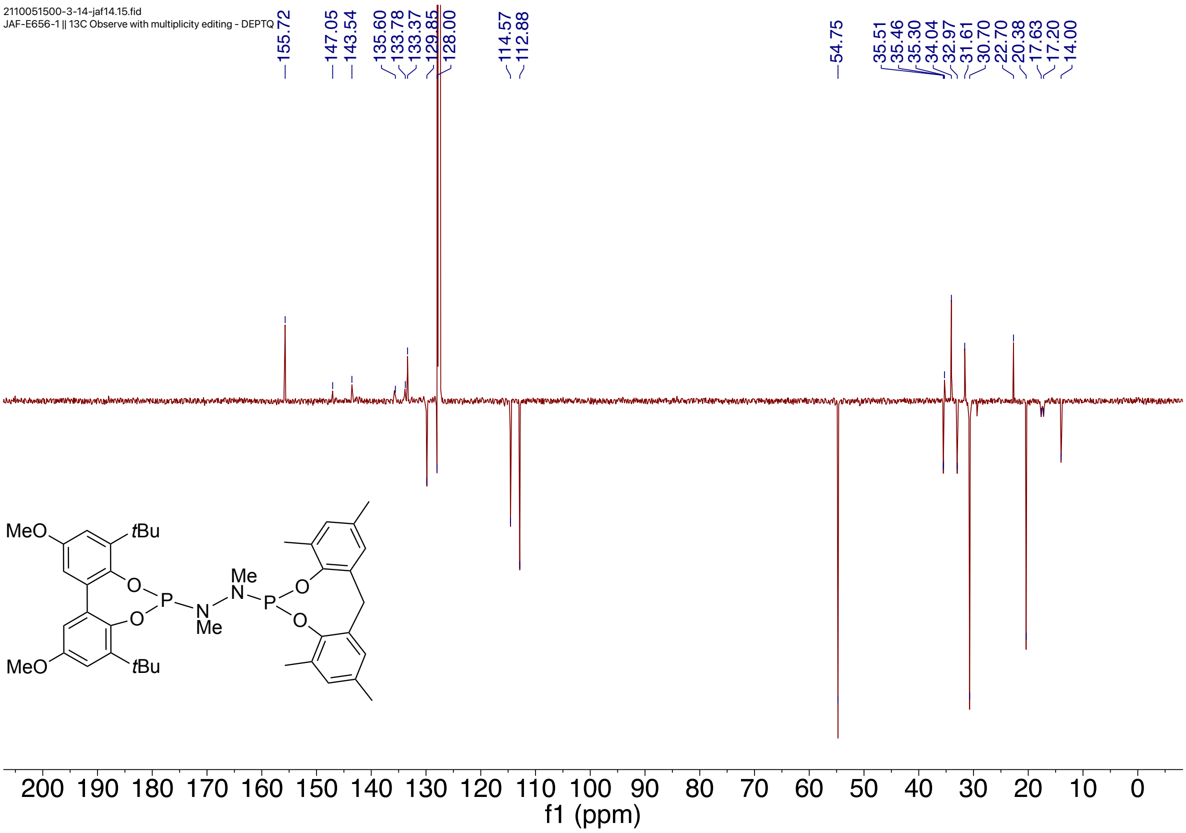

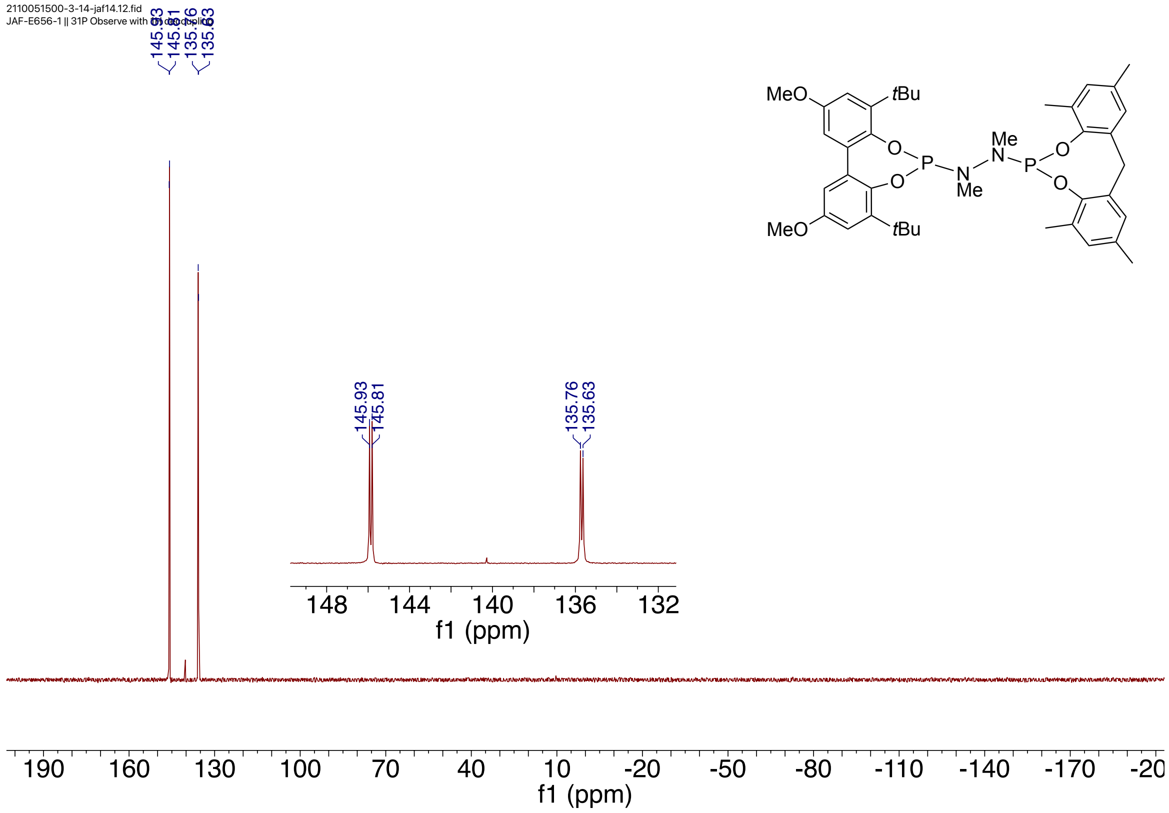


1-(4,8-di-*tert*-butyl-2,10-dimethoxydibenzo[*d*,*f*][1,3,2]dioxaphosphepin-6-yl)-1,2-dimethyl-2-(2,4,8,10,12-penta-*tert*-butyl-12*H*-dibenzo[*d*,*g*][1,3,2]dioxaphosphocin-6-yl)hydrazine, **4c**. Mix of major a minor isomers $^{31}P\{^{1}H\}$NMR (bottom). Major isomer. $^{1}H$ NMR (top next page), $^{13}C$ NMR (bottom next page), $^{31}P\{^{1}H\}$NMR (top next page).

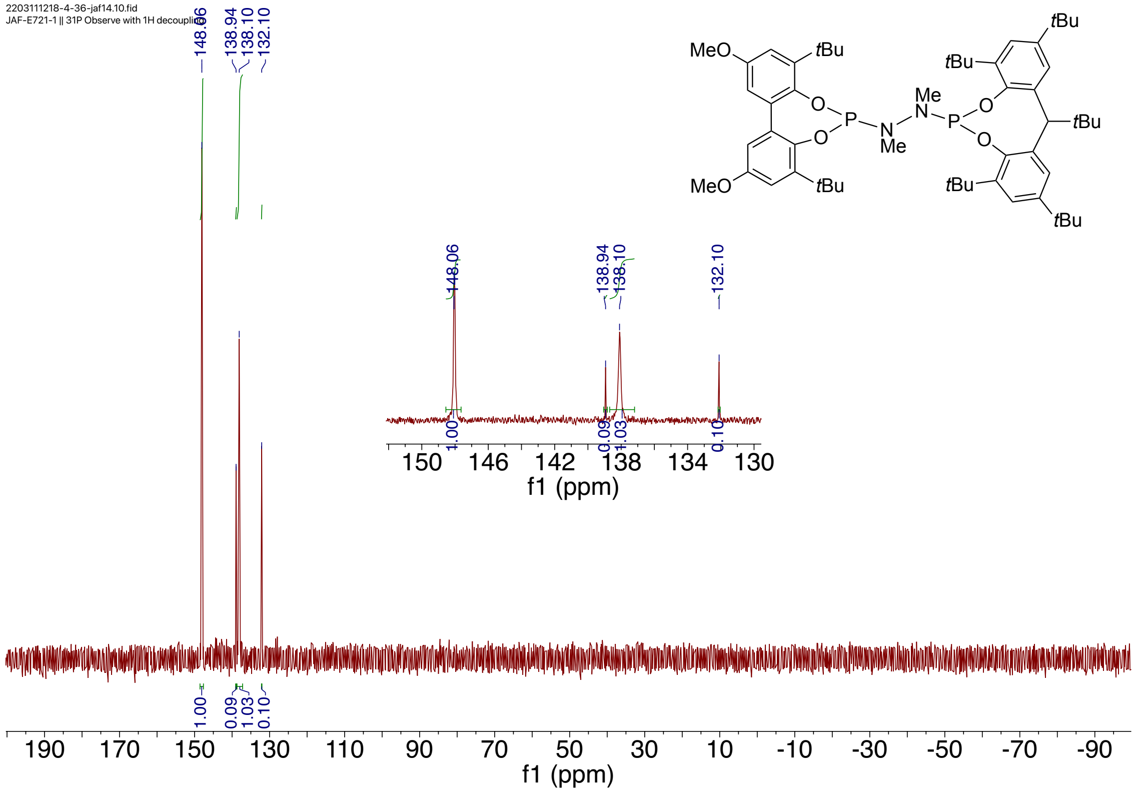

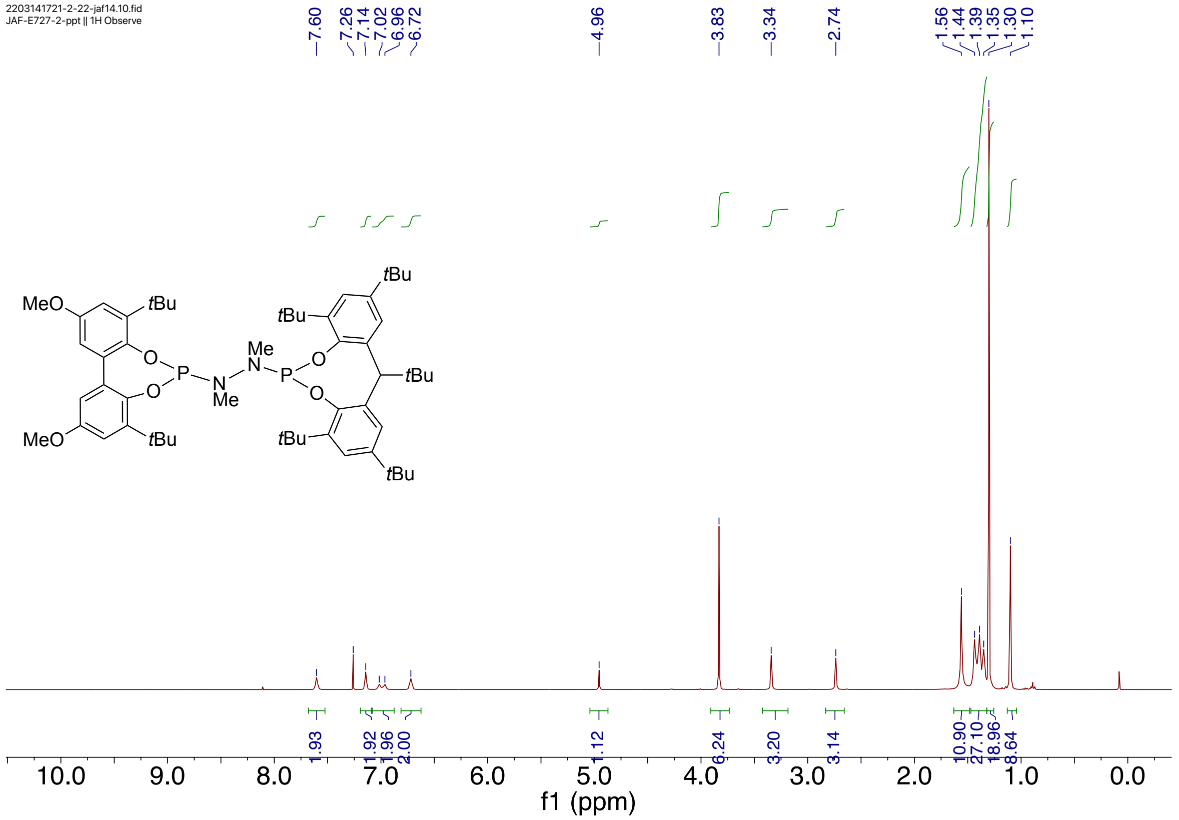
2203141721-2-22-jaf14.10.fid
JAF-E727-2-ppt || 1H Observe
7.60 7.26 7.14 7.02 6.96 6.72 4.96 3.83 3.34 2.74 1.56 1.44 1.39 1.35 1.30 1.10
MeO tBu MeO tBu O P O N Me N Me P O O tBu tBu tBu tBu
1.93 1.92 1.96 2.00 1.12 6.24 3.20 3.14 10.90 27.10 18.96 8.64
10.0 9.0 8.0 7.0 6.0 5.0 4.0 3.0 2.0 1.0 0.0
f1 (ppm)

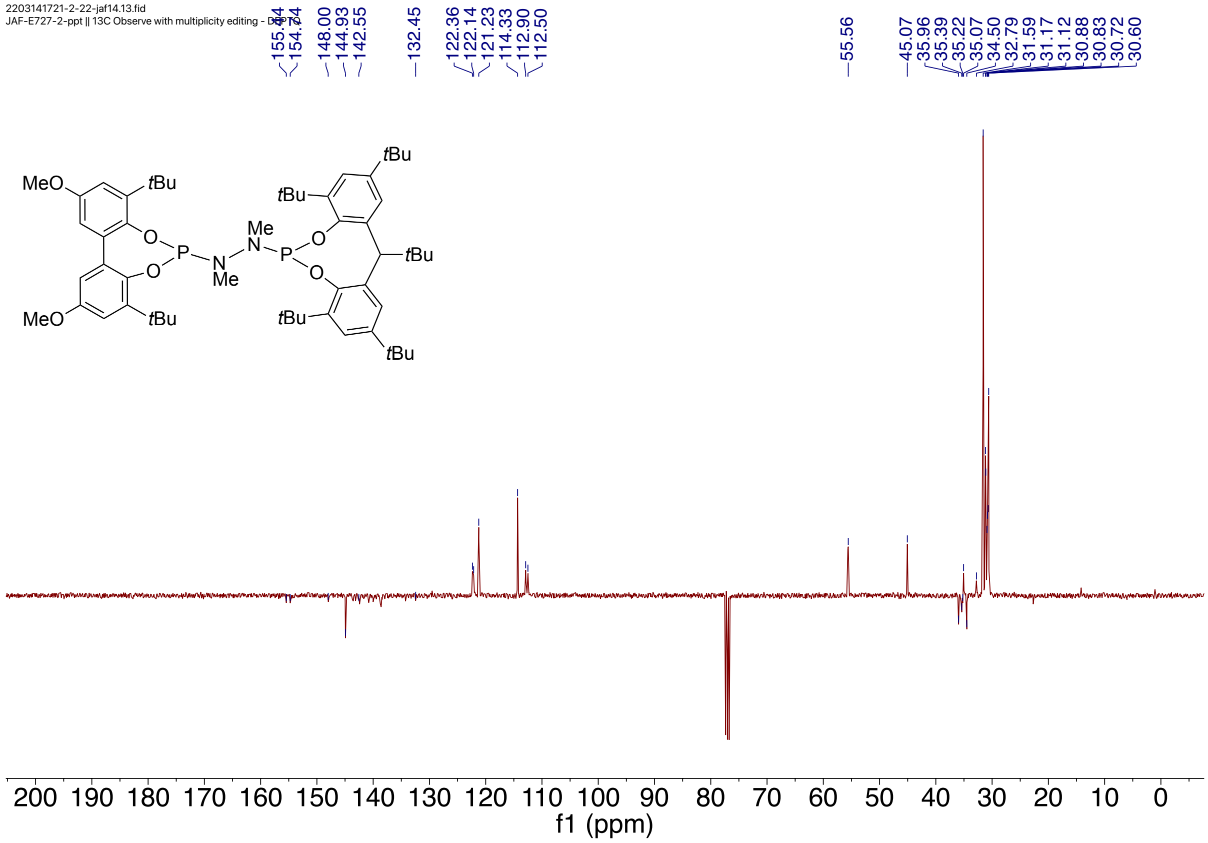
2203141721-2-22-jaf14.13.fid
JAF-E727-2-ppt || 13C Observe with multiplicity editing - DEPTQ
155.44 154.74 148.00 144.93 142.55 132.45 122.36 122.14 121.23 114.33 112.90 112.50 55.56 45.07 35.96 35.39 35.22 35.07 34.50 32.79 31.59 31.17 31.12 30.88 30.83 30.72 30.60
MeO tBu MeO tBu O P O N Me N Me P O O tBu tBu tBu tBu
200 190 180 170 160 150 140 130 120 110 100 90 80 70 60 50 40 30 20 10 0
f1 (ppm)

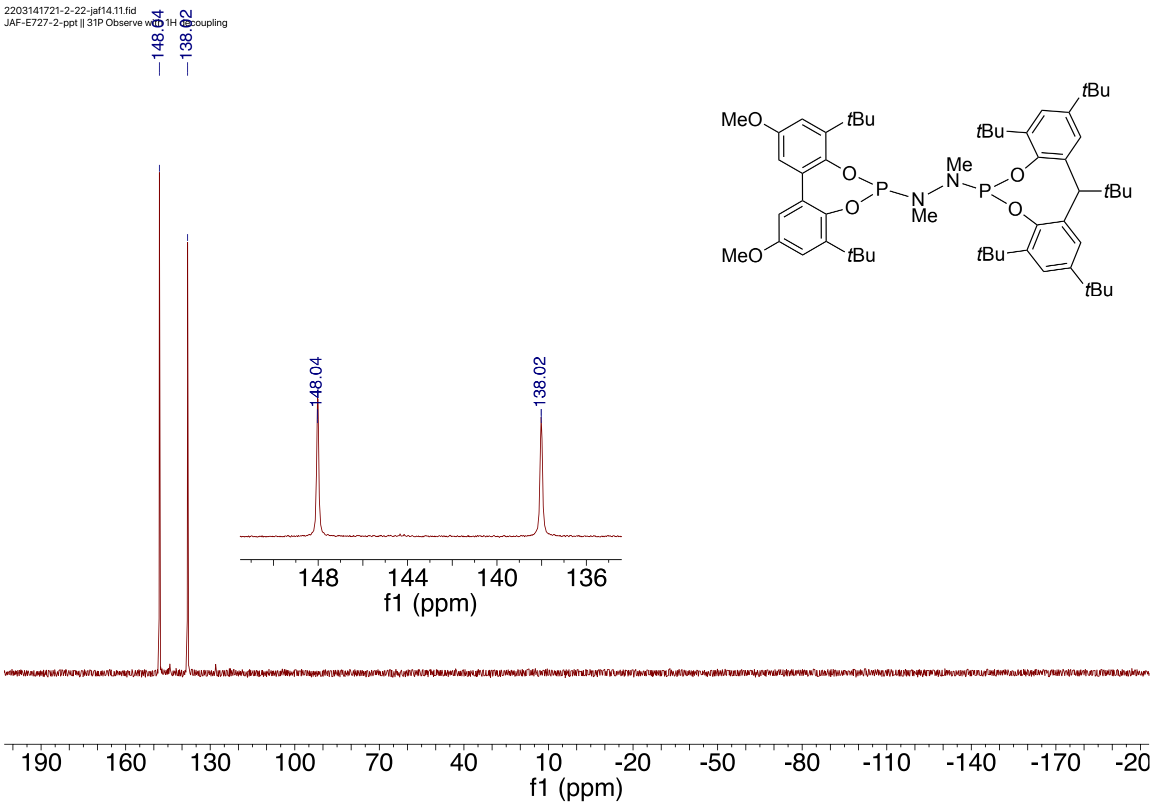
2203141721-2-22-jaf14.11.fid
JAF-E727-2-ppt || 31P Observe with 1H decoupling
148.04
138.02
148.04
138.02
148
144
140
136
f1 (ppm)
190
160
130
100
70
40
10
-20
-50
-80
-110
-140
-170
-20
f1 (ppm)
MeO
tBu
MeO
tBu
O
O
P
N
Me
N
Me
P
O
O
tBu
tBu
tBu
tBu
tBu

[Pt(**4a**)$Cl_2$] complex. $^{1}H$ NMR (top), $^{13}C$ NMR (bottom), $^{31}P\{^{1}H\}$NMR (top next page).

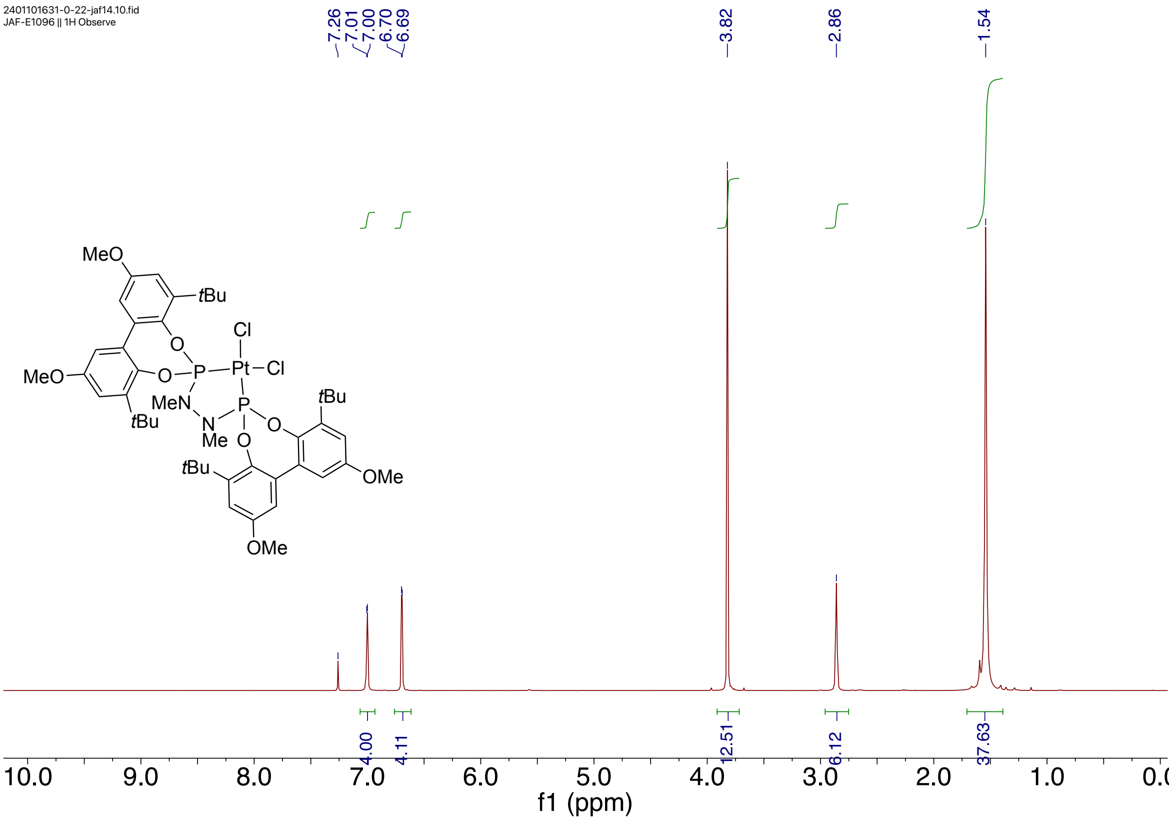


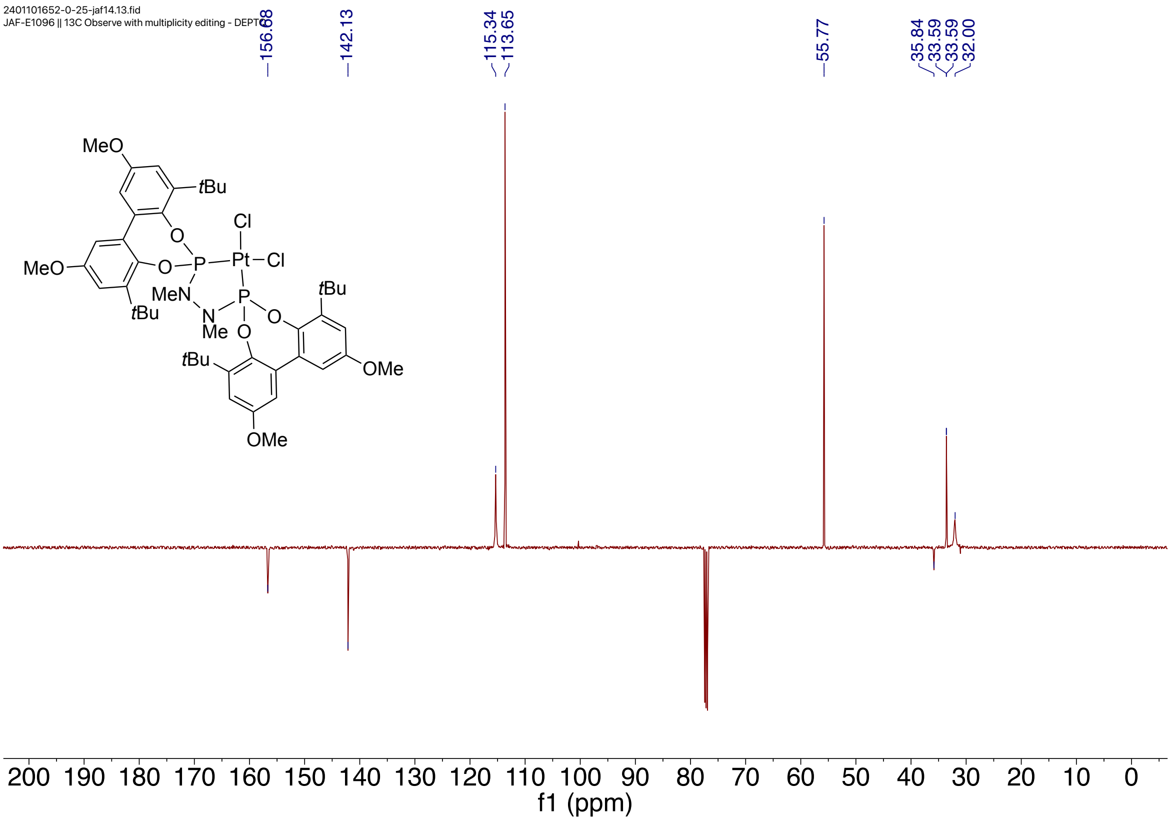

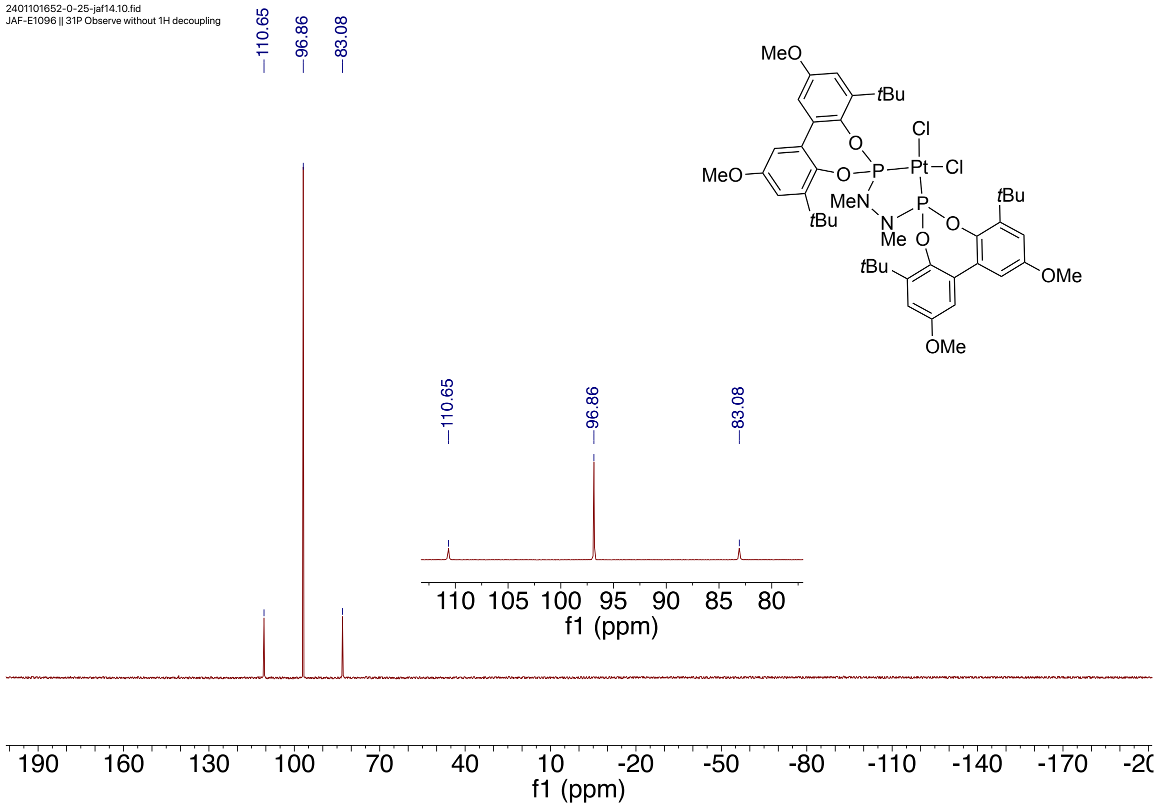


[Pt(**4b**)Cl2] complex. $^{1}$H NMR (top), $^{13}$C NMR (bottom), $^{31}$P{$^{1}$H}NMR (top next page).

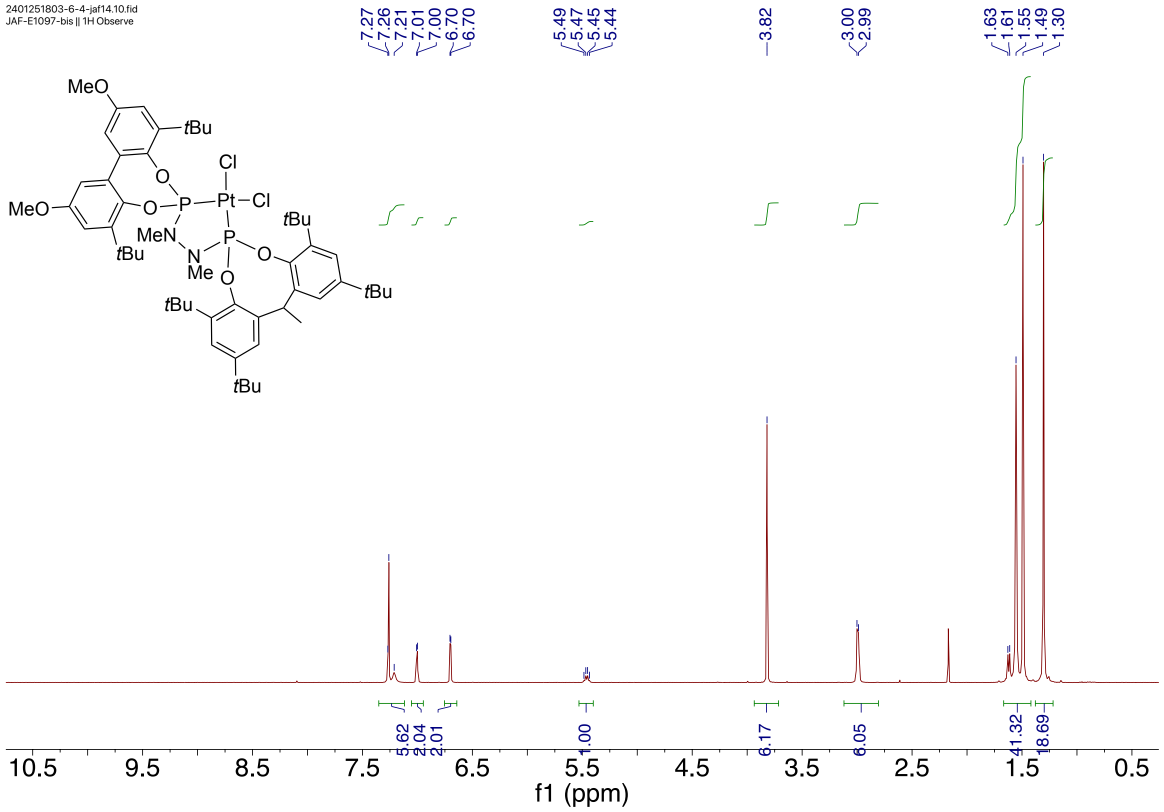

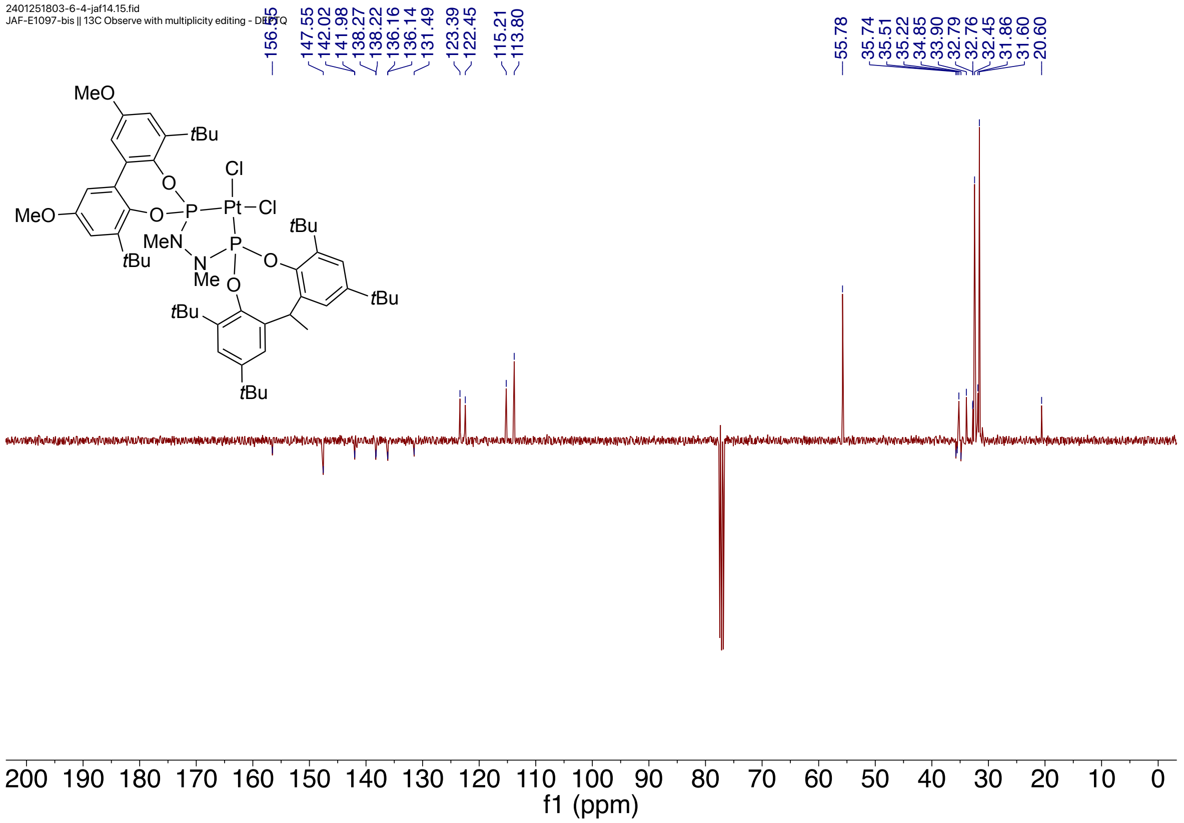
2401251803-6-4-jaf14.15.fid
JAF-E1097-bis || 13C Observe with multiplicity editing - DEPTQ
156.55
147.55
142.02
141.98
138.27
138.22
136.16
136.14
131.49
123.39
122.45
115.21
113.80
55.78
35.74
35.51
35.22
34.85
33.90
32.79
32.76
32.45
31.86
31.60
20.60
MeO
tBu
Cl
O
MeO
O
P
Pt
Cl
MeN
P
tBu
tBu
N
O
Me
O
tBu
tBu
tBu
200 190 180 170 160 150 140 130 120 110 100 90 80 70 60 50 40 30 20 10 0
f1 (ppm)

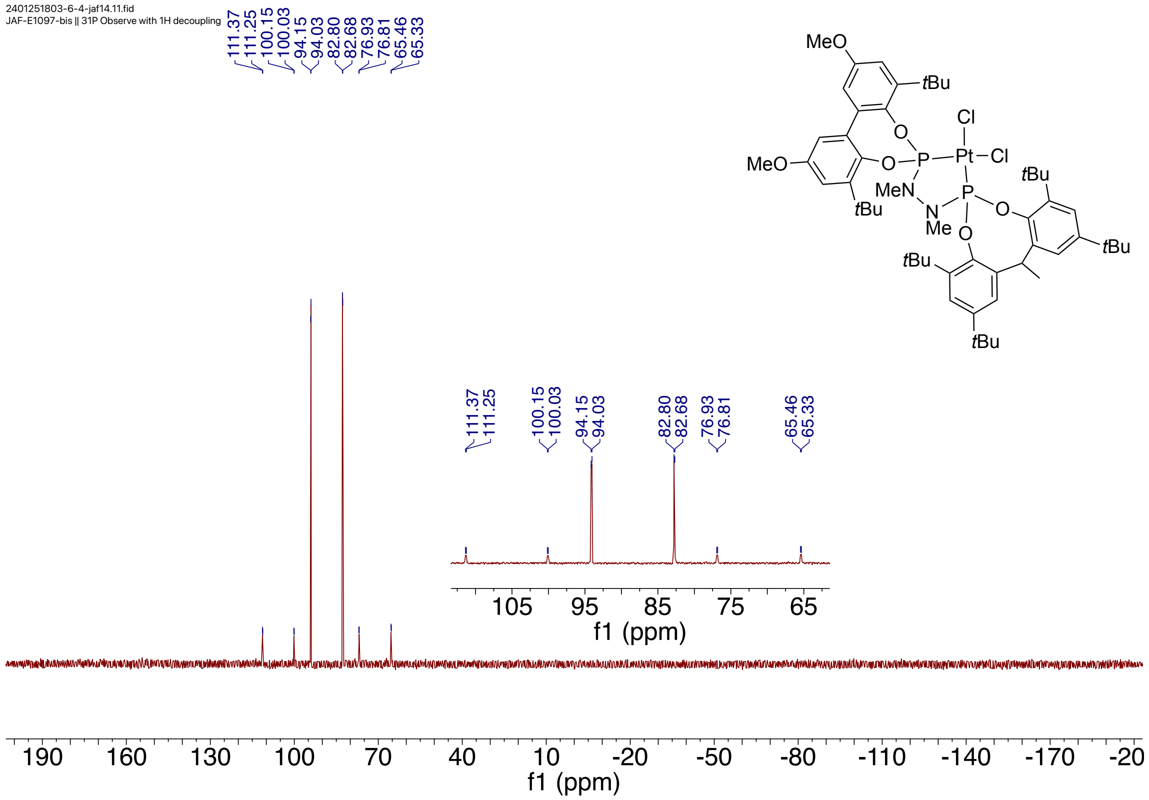
2401251803-6-4-jaf14.11.fid
JAF-E1097-bis || 31P Observe with 1H decoupling
111.37
111.25
100.15
100.03
94.15
94.03
82.80
82.68
76.93
76.81
65.46
65.33
105 95 85 75 65
f1 (ppm)
190 160 130 100 70 40 10 -20 -50 -80 -110 -140 -170 -20
f1 (ppm)

**18. References**

[10]. R. C. How, P. Dingwall, R. T. Hembre, J. A. Ponasik, G. S. Tolleson, M. L. Clarke. *Mol. Catal.* **2017**, *434*, 116-122.

[33]. D. Crozet, D. McKay, C. Bijani, A. Gual, C. Godard, C. Claver, L. Maron, M.Urrutigoïty, P. Kalck. Dalton trans. **2012**, *41*, 3369-3373.

[37]. C. J. Cobley, K. Gardner, J. Klosin, C. Praquin, C. Hill, G. T. Whiteker, A. Zanotti-Gerosa. *J. Org. Chem.* **2004**, *69*, 4031-4040.

[41]. Vlugt, J. I. V.; Hewat, A. C.; Neto, S; Sablong, R.; Mills, A. M.; Lutz, M.; Spek, A. L.; Muller, C.; Vogt, D. *Adv. Synth. Catal.* **2004**, *346*, 993-1003.

[42]. R. Tan, X. Zheng, B. Qu, C. A. Sader, K. R. Fandrick, C. H. Senanayake, X. Zhang. Organic Letters, **2016**, *18*, 3346-3349.

[43]. A. D. Allian, M. Garland. *Dalton. Trans.* **2005**, 1957-1965.

[44]. L. Théveau, R. Bellini, P. Dydio, Z. Szabo, A. van der Werf, R. A. Sander, J. N. H. Reek, C. Moberg. *Organometallics* **2016**, 35, 1956-1963.

[45]. S. Oi, M. Moro, H. Ito, Y. Honma, S. Miyano, Y. Inoue. *Tetrahedron*, **2002**, *58*, 91-97.

[46]. A. T. Straub, M. Otto, I. Usui, B. Breit. *Adv. Synth. Catal.* **2013,** *355*, 2071-2075.

[47]. I. Fleischer, L. Wu, I. Profir, R. Jackstell, R. Franke, M. Beller. *Chem. Eur. J.* **2013**, *19*, 10589-10594.

[48]. V. Bernat, C. André -Barrès, M. Baltas, N. Saffon, H. Vial. *Tetrahedron.* **2008**, *64,* 9216-9224.

[49]. J. Egger, S. Fischer, P. Bretscher, S. Freigang, M. Kopf, E. M. Carreira. *Org. Lett.* **2015**, *17*, 4340-4343.

[50]. Y. Zhang, S. Torker, M.Sigrist, N. Bregović, P. Dydio. *J. Am. Chem. Soc.* **2020**, *142*, 18251-18265.

[51]. *CrysAlisPro* v1.171.42.74 & 96a Rigaku Oxford Diffraction, Rigaku Corporation, *Tokyo,* Japan, 2023.

[52]. G. M. Sheldrick. SHELXT – Integrated space-group and crystal structure determination. *Acta Crystallogr., Sect. A: Found. Adv.* **2015**, *71*, 3-8. doi: 10.1107/S2053273314026370.

[53]. G. M. Sheldrick. Crystal structure refinement with SHELXL. *Acta Crystallogr., Sect. C: Struct. Chem.* **2015**, *71*, 3-8. Doi: 10.1107/S2053229614024218.

[54]. O. V. Dolomanov, L. J. Bourhis, R. J. Gildea, J. A. K. Howard, H. Puschmann. OLEX2: a complete structure solution, refinement and analysis program. *J. Appl. Crystallogr.* **2009**, *42*, 339-341. doi: 10.1107/S0021889808042726.
